# Supplementary material for: Genetically encoded Nδ-vinyl histidine for the evolution of enzyme catalytic center
Source: Nat Commun. 2024 Jul 8;15:5714. doi: 10.1038/s41467-024-50005-9 (PMC11231154; doi:10.1038/s41467-024-50005-9)
Supplement: Supplementary file 1 — Supplementary Information [file 41467_2024_50005_MOESM1_ESM.pdf]

Supplementary Information

Genetically Encoded Nδ-Vinyl Histidine for the Evolution of Enzyme Catalytic Center

Haoran Huang[†, #], Tao Yan[†, #], Chang Liu[†, #], Yuxiang Lu[†], Zhigang Wu[†], Xingchu Wang[†], Jie Wang[†, *]

[†]Department of Chemistry, Research Center for Chemical Biology and Omics Analysis, College of Science, Guangdong Provincial Key Laboratory of Catalysis, Southern University of Science and Technology, Shenzhen 518055, China

[#]These authors contributed equally to this work.

[*]Correspondence to: wangjie@sustech.edu.cn (J.W.)

Supplementary Methods **1**

1. Chemical synthesis of Nδ-vinyl histidine (δVin-H).. 1

2. Chemical synthesis of resorufin acetyl methoxymethyl ether (A-Me-Res).. 2

3. Chemical synthesis of ethyl 2-(dimethyl(phenyl)silyl) acetate by whole-cell catalysis.. 2

Supplementary Figures (1-58) **3**

Supplementary Tables (1-6) **66**

Supplementary References **80**

Supplementary Methods

1. Chemical synthesis of Nδ-vinyl histidine (δVin-H)

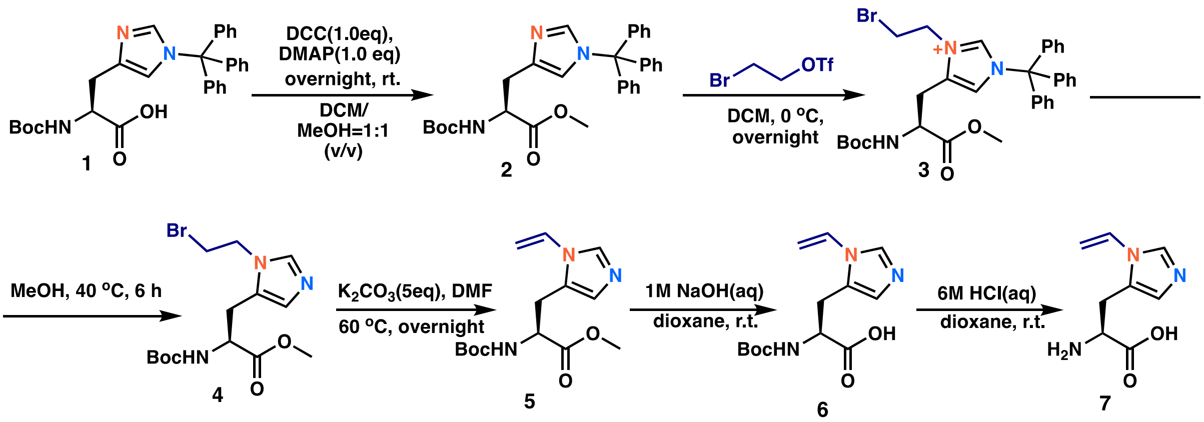

MeOH (100 mL) was added to a stirred solution of protected histidine **1** (50 mmol), DCC (55 mmol), and DMAP (50 mmol) in DCM (50 mL) in an ice bath. The reaction mixture was gradually warmed to room temperature and stirred for 12 hours. The completion of the reaction was monitored by TLC. After completion, the mixture was concentrated under reduced pressure, dissolved in EtOAc, and filtered through a Celite pad. The filtrate was evaporated and purified by flash column chromatography to give product **2** (yield: 90%). ^{1}H NMR (400 MHz, $CDCl_3$) δ 7.43 – 7.28 (m, 10H), 7.15 – 7.05 (m, 6H), 6.53 (s, 1H), 5.99 (d, J = 8.4 Hz, 1H), 4.52 (m, 1H), 3.59 (s, 3H), 3.04 (dd, J = 14.6, 5.2 Hz, 1H), 2.97 (dd, J = 14.4, 4.8 Hz, 1H), 1.41 (s, 9H). ^{13}C NMR (101 MHz, $CDCl_3$) δ 155.67, 142.33, 138.76, 136.48, 129.84, 128.17, 128.15, 119.69, 79.61, 75.40, 53.83, 52.11, 30.34, 28.44. HRMS: m/z calculated for $[M+H]^+$: 512.2549; Found: 512.2533.

Freshly prepared 2-bromoethyl trifuoromethanesulfonate (44 mmol) was added to a stirred solution of protected histidine **2** (40 mmol) in dry DCM (160 mL) [1]under a nitrogen atmosphere in an ice bath. The reaction mixture was gradually warmed to room temperature and stirred for 12 hours. The completion of the reaction was monitored by TLC. After completion, the mixture was concentrated under reduced pressure to give compound **3** without purification and the solid obtained after concentration was further dissolved in 40 mL of MeOH at 40 °C for another 6 hours. The mixture was evaporated and purified by flash column chromatography to give product **4** (yield: 54%). ^{1}H NMR (400 MHz, $CDCl_3$) δ 7.99 (s, 1H), 6.89 (s, 1H), 5.29 (d, J = 7.6 Hz, 1H), 4.53 (m, 1H), 4.47 – 4.28 (m, 1H), 3.76 (s, 2H), 3.62 (t, J = 6.5 Hz, 2H), 3.18 (dd, J = 15.7, 5.7 Hz, 1H), 3.11 (dd, J = 15.6, 5.9 Hz, 1H), 1.42 (s, 9H). ^{13}C NMR (101 MHz, $CDCl_3$) δ 171.42, 155.35, 137.82, 127.70, 125.67, 80.64, 53.09, 52.92, 46.64, 30.05, 28.35, 26.78. HRMS: m/z calculated for $[M+H]^+$: 376.0872; Found: 376.0862.

K_2CO_3 (100 mmol) was added to a mixture of resulting compound **4** (20 mmol) and dry DMF (80 mL) under a nitrogen atmosphere. The mixture was stirred at 60 °C for 12 hours. The completion of the reaction was monitored by TLC. Then, the reaction mixture was cooled to room temperature, dissolved in EtOAc, and filtered through a Celite pad. The mixture was then extracted with EtOAc and dried over $MgSO_4$. The filtrate was evaporated and purified by flash column chromatography to give product **5** (47% yield). ^{1}H NMR (400 MHz, $CDCl_3$) δ 7.57 (s, 1H), 6.72 – 6.61 (m, 2H), 5.66 (d, J = 7.9 Hz, 1H), 5.21 (d, J = 15.6 Hz, 1H), 4.84 (d, J = 8.8 Hz, 1H), 4.33 (m, 1H), 3.54 (s, 3H), 2.99 (dd, J = 15.5, 5.7 Hz, 1H), 2.92 (dd, J = 15.4, 6.3 Hz, 1H), 1.24 (s, 9H). ^{13}C NMR (101 MHz, $CDCl_3$) δ 171.28, 154.89, 134.53, 128.40, 127.78, 125.70, 104.63, 79.53, 52.82, 52.10, 27.94, 26.29. HRMS: m/z calculated for $[M+H]^+$: 296.1610; Found: 296.1601.

NaOH solution (2 N, 5 mL) was added to a stirred solution of protected Nδ-vinylhistidine **5** (9 mmol) in dioxane (5 mL), and the mixture was stirred at room temperature for 2 hours. The completion of the reaction was monitored by TLC. The pH of the reaction mixture was adjusted to 7. After that, the mixture was evaporated and dissolved in DCM. Then, the mixture was filtered through a Celite pad. After that, the DCM was evaporated in vacuo to give product **6**.

The resulting compound **6** was dissolved in HCl (6 M, 10 mL), and the reaction was allowed to stir for 6 h at room temperature. The volatiles were subsequently evaporated, and the residue was redissolved in MeOH (5 mL) and precipitated into Et_2O (250 mL), giving product **7** as a white solid in 69% yield. ^1H NMR (400 MHz, MeOD) δ 9.35 (s, 1H), 7.68 (s, 1H), 7.28 (dd, *J* = 15.2, 8.4 Hz, 1H), 6.00 (dd, *J* = 15.2, 2.1 Hz, 1H), 5.69 (dd, *J* = 8.4, 2.2 Hz, 1H), 4.38 (t, *J* = 7.1 Hz, 1H), 3.62 – 3.45 (m, 2H). ^{13}C NMR (101 MHz, MeOD) δ 168.61, 134.40, 128.12, 126.85, 119.58, 115.05, 50.95, 23.96. HRMS: m/z calculated for $[M+H]^+$: 182.0925; Found: 182.0923.

2. Chemical synthesis of resorufin acetyl methoxymethyl ether (A-Me-Res)

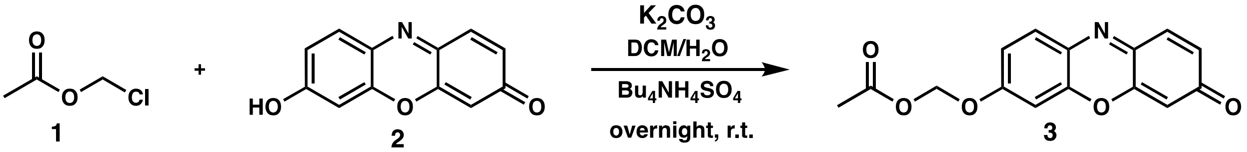

Following the literature procedure[2], resorufin **2** (50 mg, 0.235 mmol) and anhydrous potassium carbonate (K_2CO_3) were dissolved in H_2O (2.4 mL) to obtain a dark red solution. *N,N,N*-Tributyl-1-butanaminium sulfate 50 wt. % in H_2O (150 mg, 0.286 mmol) was added, followed by chloromethyl acetate **1** (94 µL, 1.304 mmol) in DCM. The reaction mixture was stirred at room temperature overnight. The reaction mixture was then diluted with H_2O and DCM. The aqueous layer was extracted with DCM, and the combined organics were washed with H_2O and saturated brine three times. The organic layer was dried over anhydrous Na_2SO_4 and concentrated under reduced pressure to give a purple oil. Purification via column chromatography afforded the compound resorufin acetyl methoxymethyl ether as a red–orange solid in 37% yield. ^1H NMR (600 MHz, $CDCl_3$) δ 7.74 (d, J = 8.8 Hz, 1H), 7.42 (d, J = 9.7 Hz, 1H), 7.04 (dd, J = 8.9, 2.7 Hz, 1H), 6.99 (d, J = 2.7 Hz, 1H), 6.84 (dd, J = 9.7, 1.9 Hz, 1H), 6.33 (d, J = 1.9 Hz, 1H), 5.84 (s, 2H), 2.16 (s, 2H). HRMS: m/z calculated for $[M+H]^+$: 286.0715; Found: 286.0707.

3. Chemical synthesis of ethyl 2-(dimethyl(phenyl)silyl) acetate by whole-cell catalysis

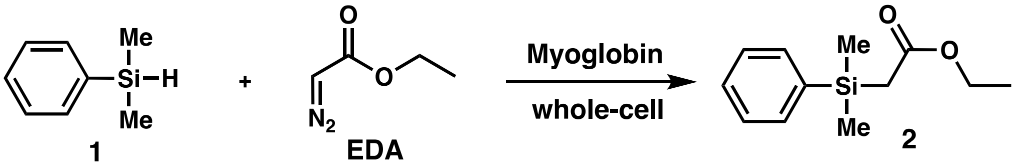

E. coli BL21(DE3) cells expressing wild-type myoglobin (H64V, V68A) were prepared according to the protocol described above. After harvesting, the cells were suspended in PBS buffer and diluted to an OD600 of 40. The cell suspension was transferred to an open Erlenmeyer flask equipped with a stir bar and supplemented with 50 mM D-glucose solution (from a 2 M stock solution). Reactions were initiated by the dropwise addition of compound **1** (from a 2 M stock solution in ethanol, final concentration: 20 mM), followed by the dropwise addition of EDA (from a 2 M stock solution in ethanol, final concentration: 20 mM). The reaction mixtures were stirred at room temperature for 20 hours and extracted twice with ethyl acetate. The combined organic layers were dried over sodium sulfate and evaporated under reduced pressure. The crude product was purified by flash column chromatography to give product **2** in 22% yield. ^1H NMR (600 MHz, $CDCl_3$) δ 7.56 – 7.51 (m, 2H), 7.42 – 7.34 (m, 3H), 4.04 (q, *J* = 7.2 Hz, 2H), 2.11 (s, 2H), 1.16 (t, *J* = 7.1 Hz, 2H), 0.41 (s, 6H).

Supplementary Figures (1-58)

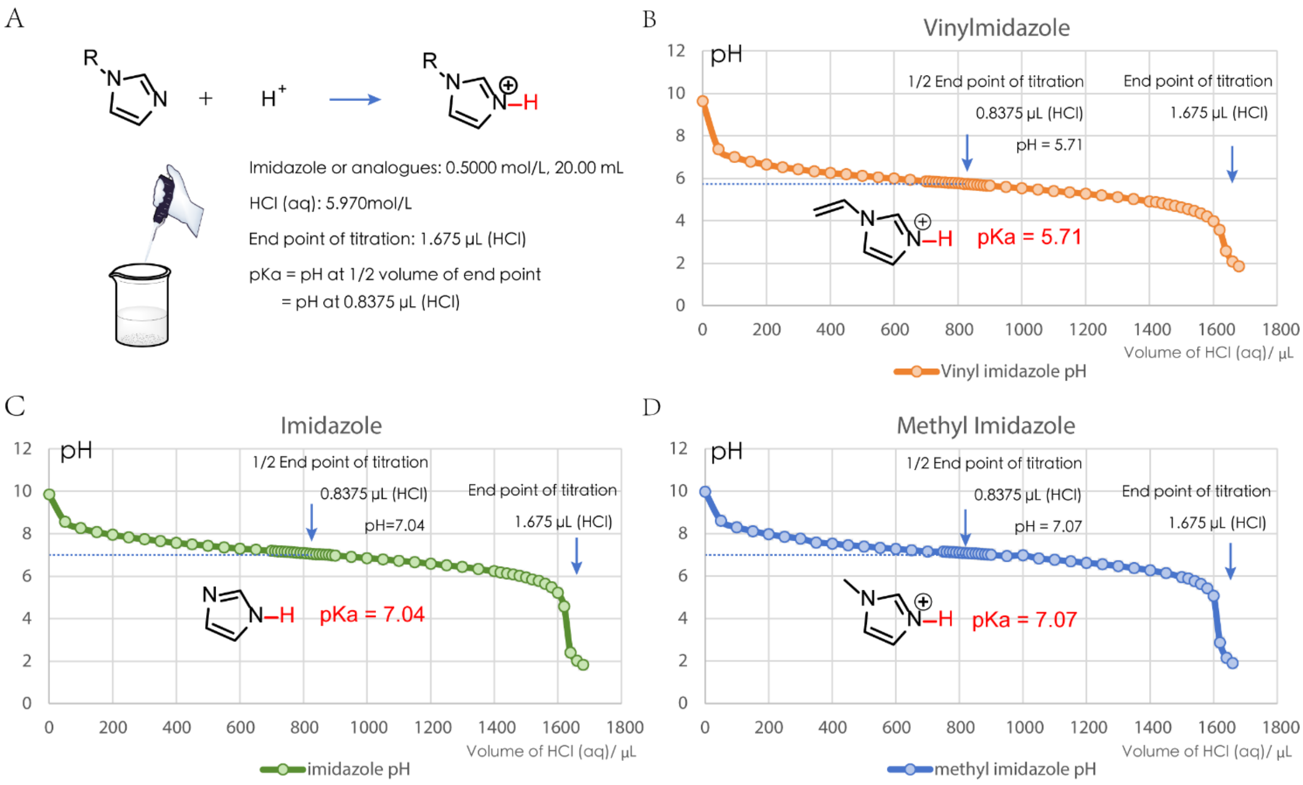

Supplementary Figure 1: Measurement of pka of imidazole analogs. (A) Principle of weak-base pKa determination. (B) Titration curve of vinyl imidazole. (C) Titration curve of imidazole. (D) Titration curve of methyl imidazole. The data is the representative data from similar results after three independent experiments.

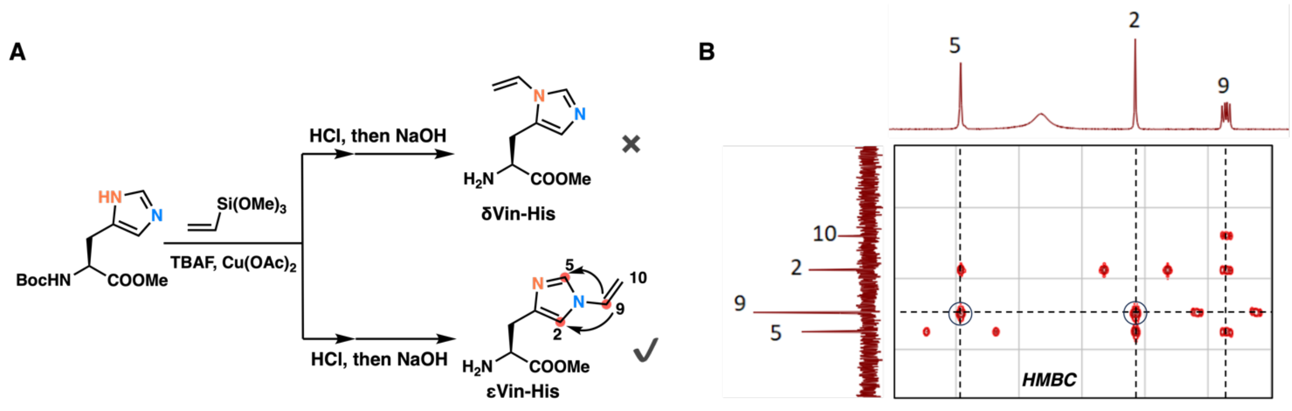

Supplementary Figure 2：Direct synthesis of Nδ-vinyl histidine by a metal-catalyzed C–N bond formation reaction. (A) Synthetic routes for direct vinylation of histidine. (B) HMBC NMR verification of the structure obtained by direct histidine vinylation.

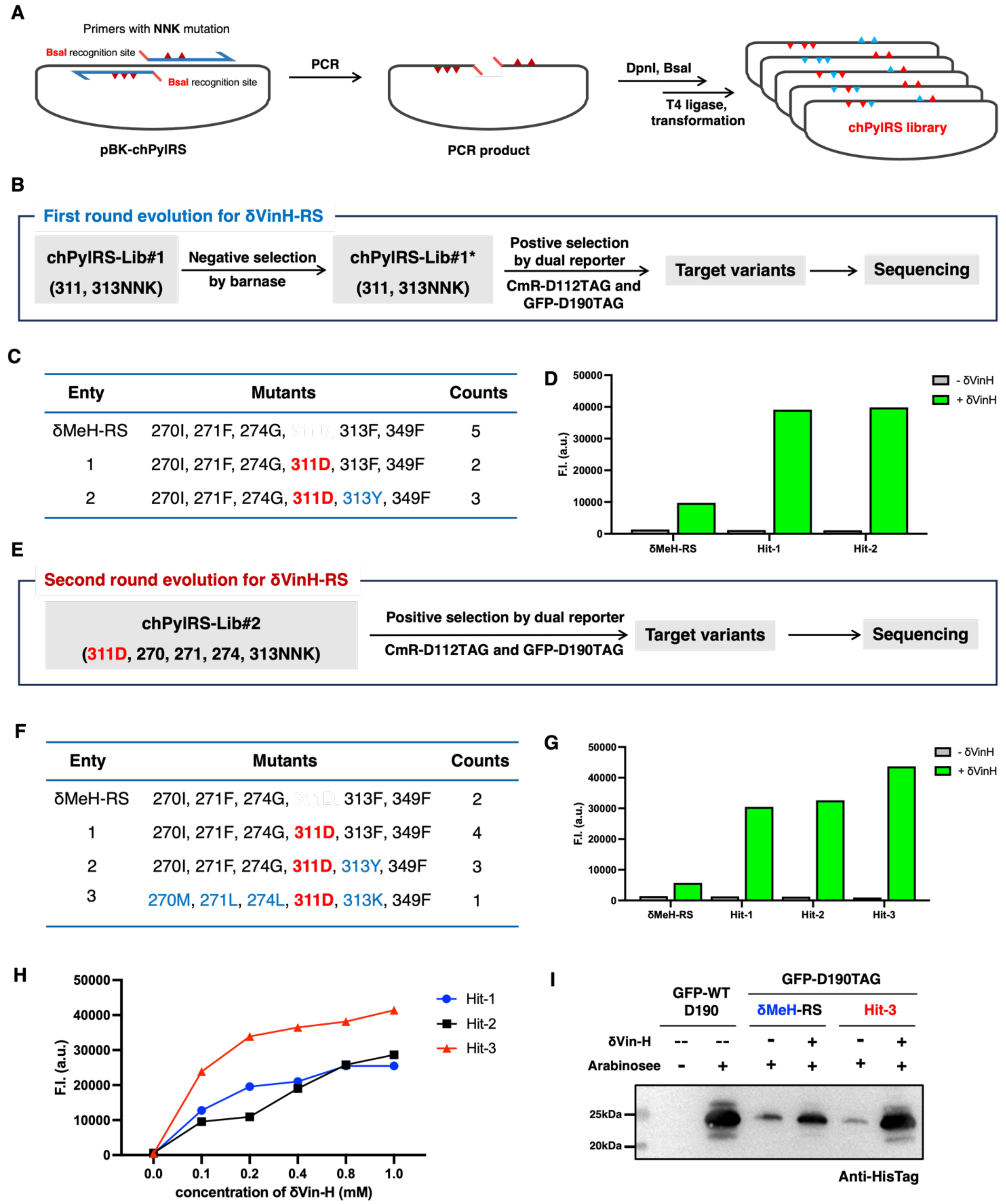

Supplementary Figure 3: The workflow of the direct evolution of δVinH-RS. (A) Construction workflow of the PylRS gene library. (B) First-round evolution of δVinH-RS by negative and positive selection. (C) PylRS sequence results of positive clones in the first round of evolution. (D) Selection results of the positive clones in the presence of δVin-H by the GFP reporter in the first round of evolution. (E) Second round of evolution for δVinH-RS by positive selection. (F) PylRS sequence results of positive clones in the second round of evolution. (G) Selection results of the positive clones in the presence of δVin-H by the GFP reporter in the second round of evolution. (H-I) Validation of the different δVinH-RS variants through amber codon suppression of GFP-N190TAG in DH10B *E. coli*. The data in Figure D, G, H and I is the representative data from similar results after three independent experiments.

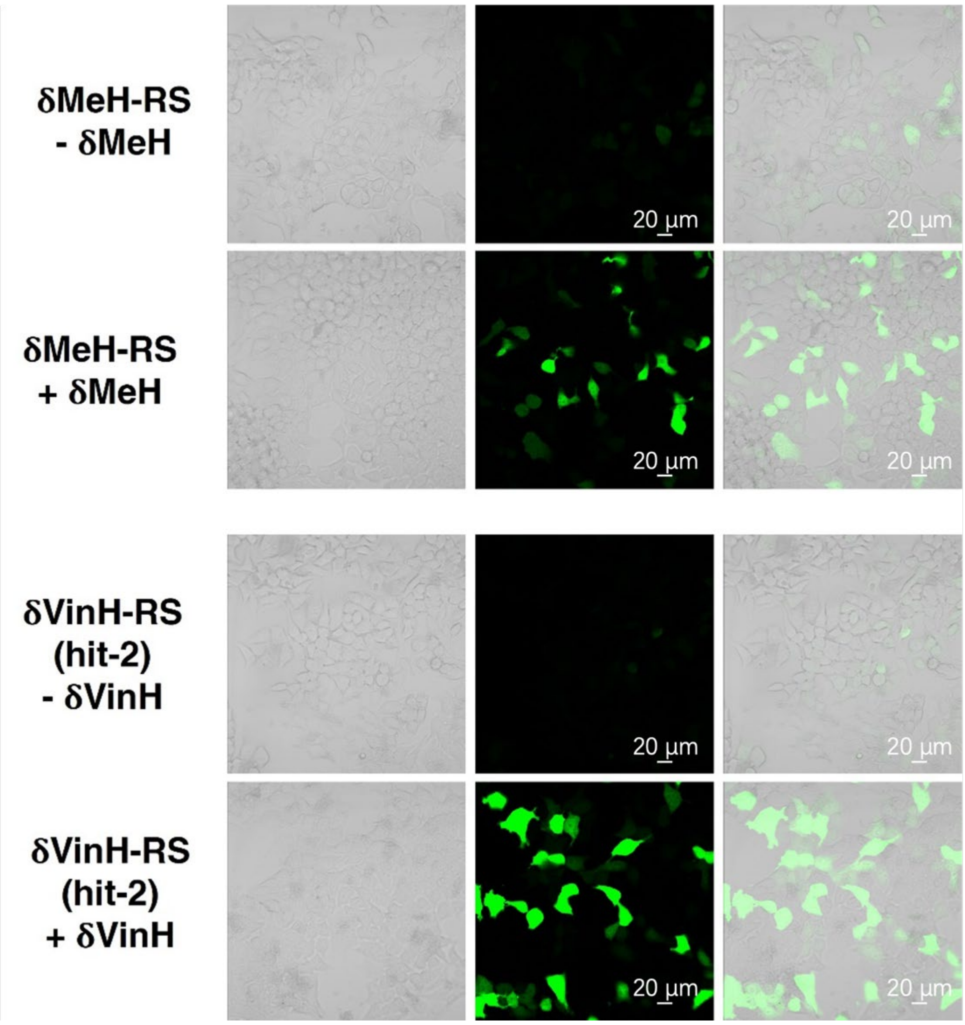

Supplementary Figure 4: Fluorescence images of the incorporation of δVin-H into GFP-Y40TAG in HEK 293T cells. The data is the representative data from similar results after three independent experiments.

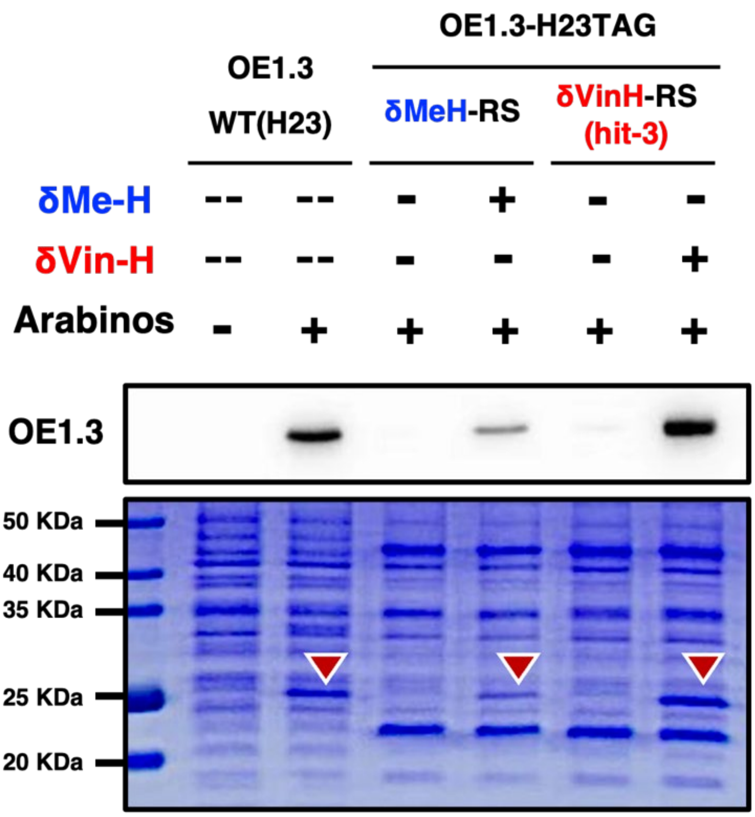

Supplementary Figure 5: SDS–PAGE analysis of the incorporation efficiency of δVin-H into OE1.3-H23TAG. The data is the representative data from similar results after three independent experiments.

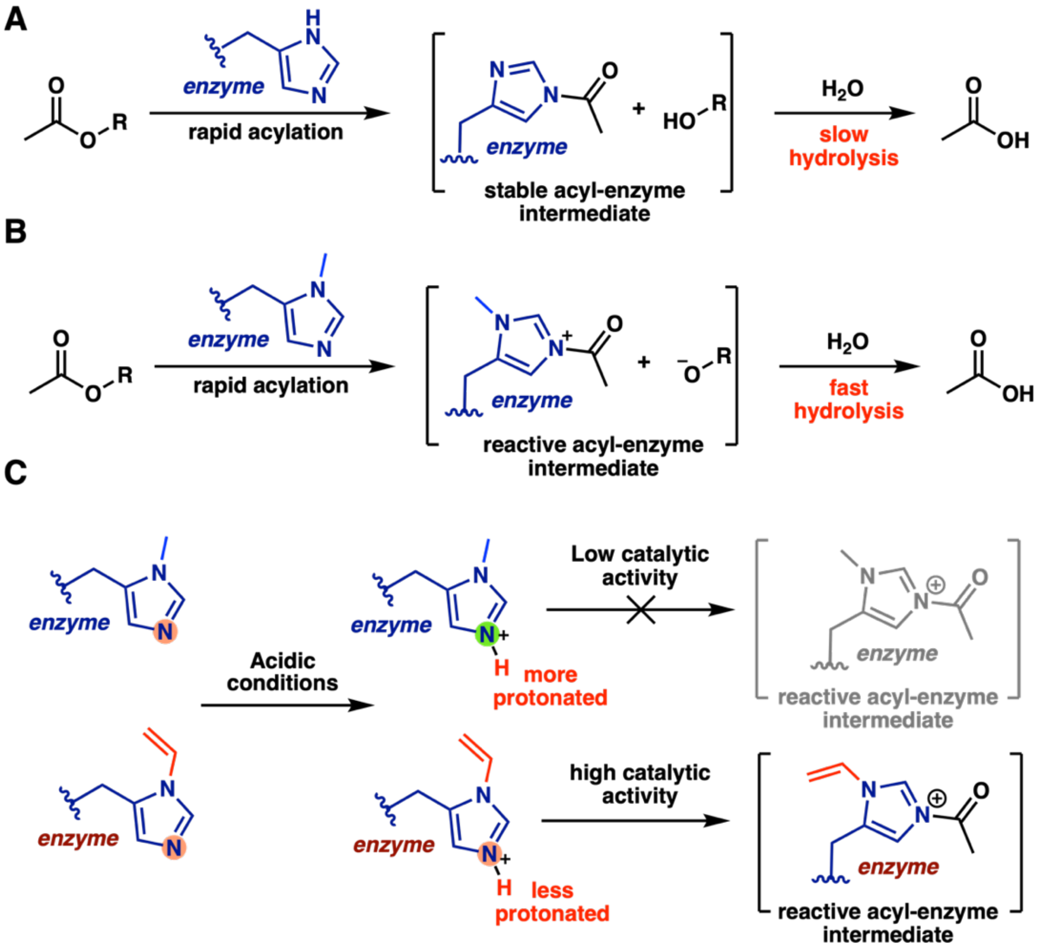

Supplementary Figure 6: Proposed mechanism of the ester hydrolysis reaction in the catalytic center of the histidine analogs. (A) Histidine ester hydrolysis catalyzed by histidine under neutral conditions. (B) Ester hydrolysis catalyzed by Nδ-methyl histidine under neutral conditions. (C) Ester hydrolysis catalyzed by Nδ-vinyl histidine under acidic conditions.

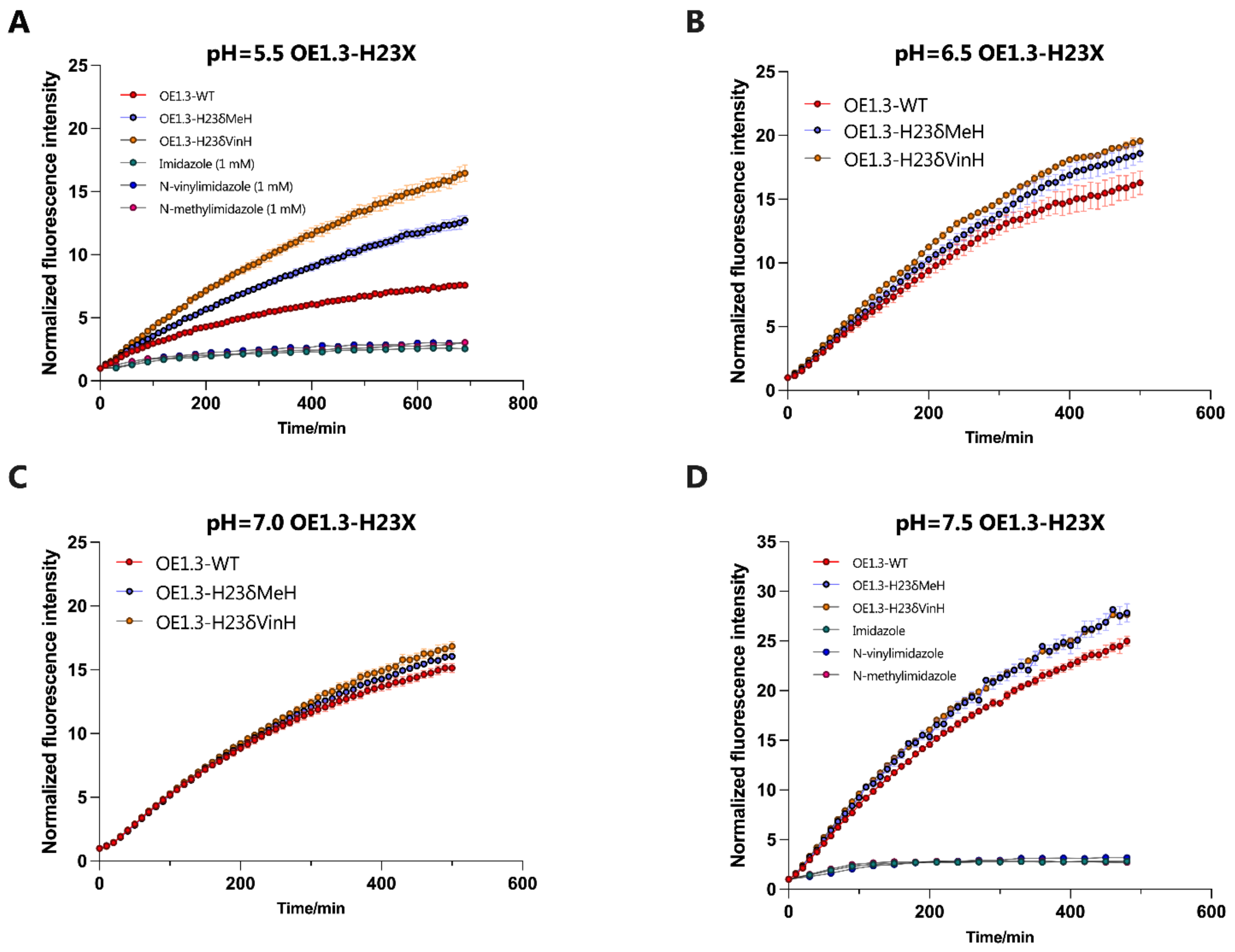

Supplementary Figure 7: Comparison of the hydrolysis activity of resorufin acetyl methoxymethyl ether (A-Me-Res) catalyzed by OE1.3-His, δMeH and δVinH at different pH values. (A) pH=5.5. (B) pH=6.5. (C) pH=7.0. (D) pH=7.5. The data in Figure A-D are presented as mean values ± SEM (n=3 independent experiments).

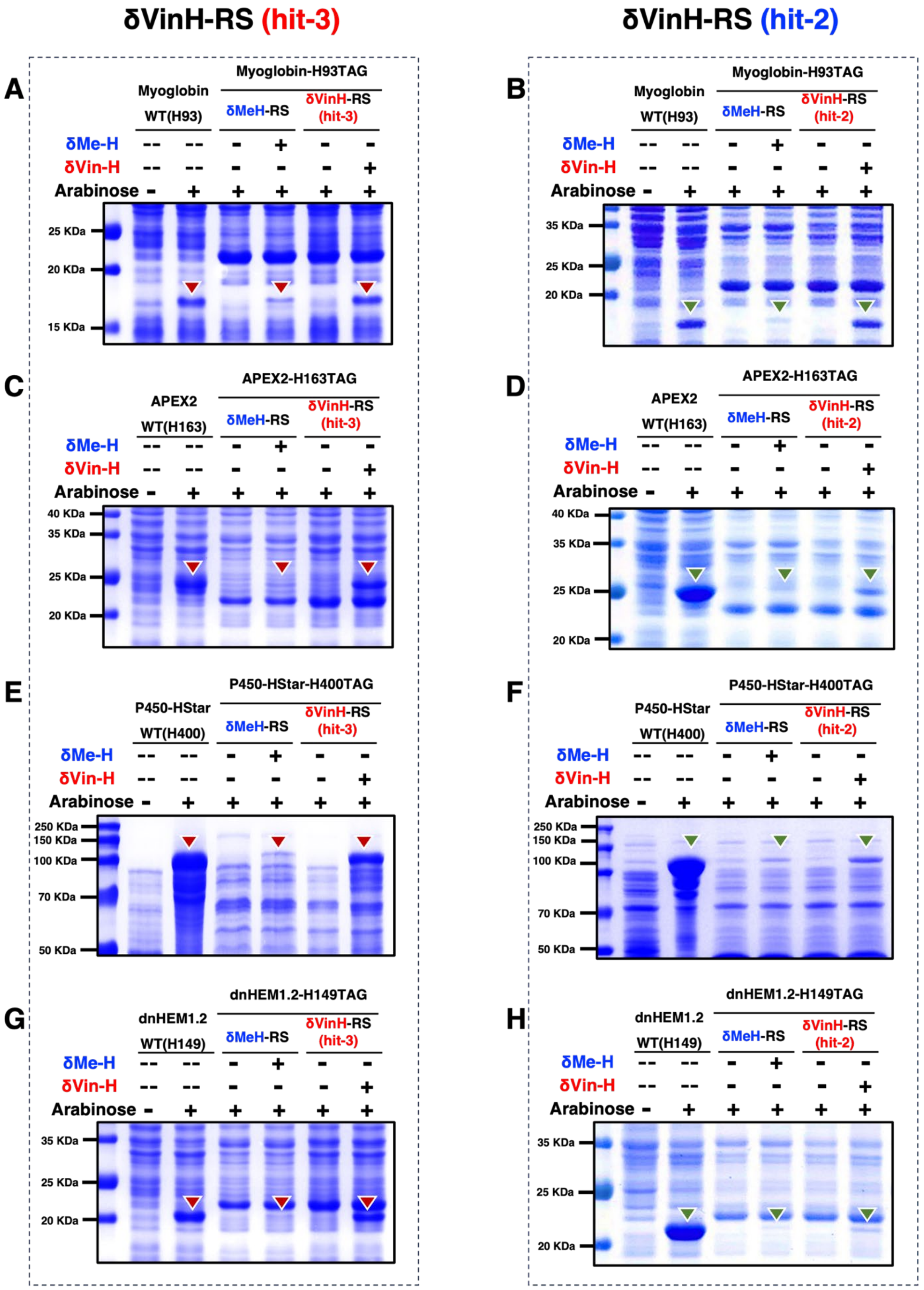

Supplementary Figure 8: SDS–PAGE analysis of the incorporation efficiency of δVin-H into four different heme-dependent proteins by different PylRS variants. (A-B) Myoglobin-H93TAG. (C-D) APEX2-H163TAG. (E-F) P450-BM3-HStar-H400TAG. (G-H) dnHEM1.2-H149TAG. The data is the representative data from similar results after three independent experiments.

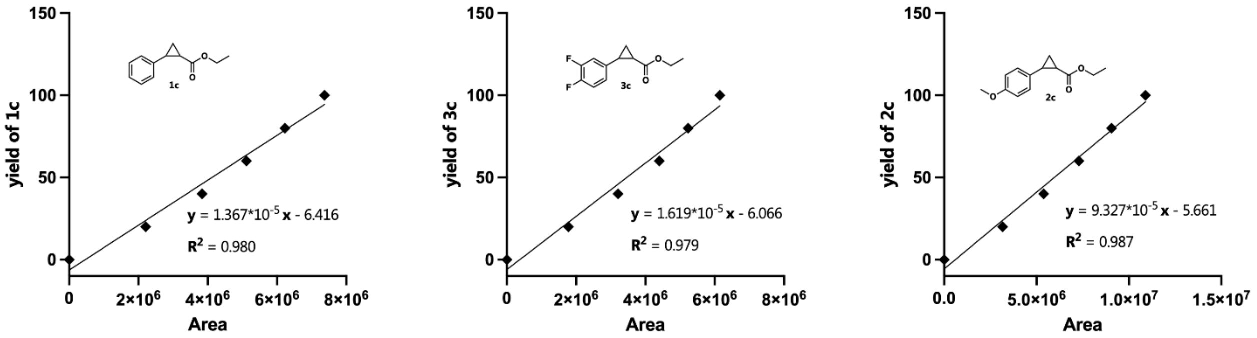

Supplementary Figure 9: Standard curve for determination of cyclopropane product concentrations. The data is the representative data from similar results after three independent experiments.

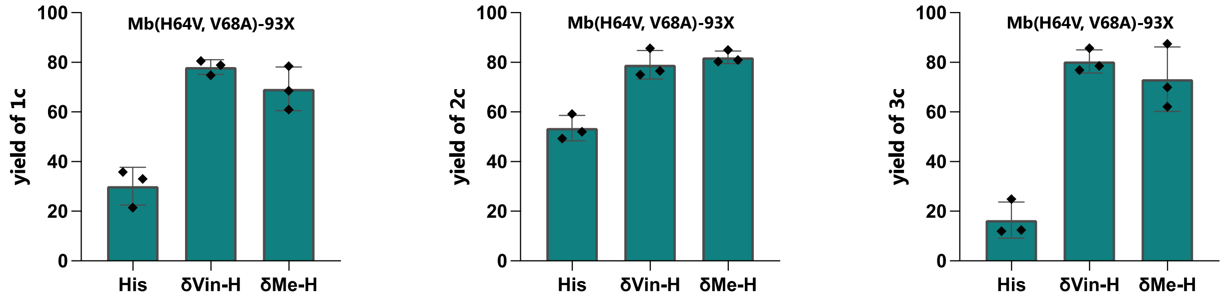

Supplementary Figure 10: Mb-catalyzed cyclopropanation in air. Conversions for myoglobin-catalyzed cyclopropanation reactions by using different Mb(H64V, V68A) variants under aerobic conditions. Reaction conditions: 10 μM enzyme, 10 mM styrene, 20 mM EDA and 10 mM dithionite. The data are presented as mean values ± SD (n=3 independent experiments).

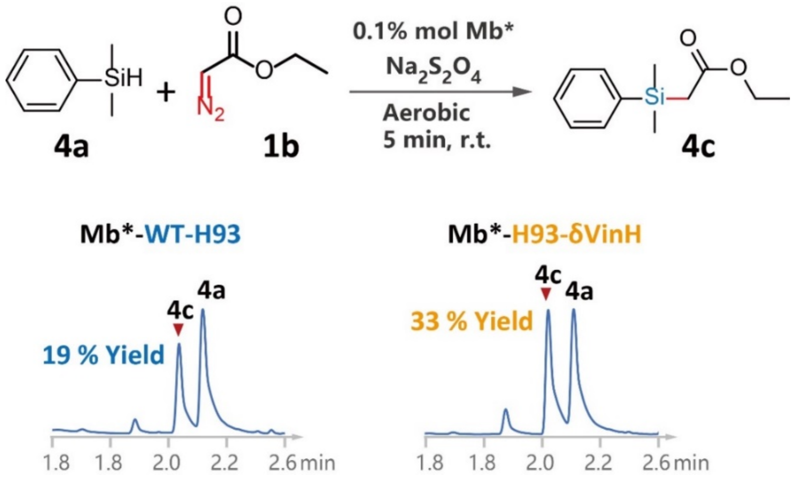

Supplementary Figure 11: Mb-catalyzed Si–H insertion reaction. The data is the representative data from similar results after three independent experiments.

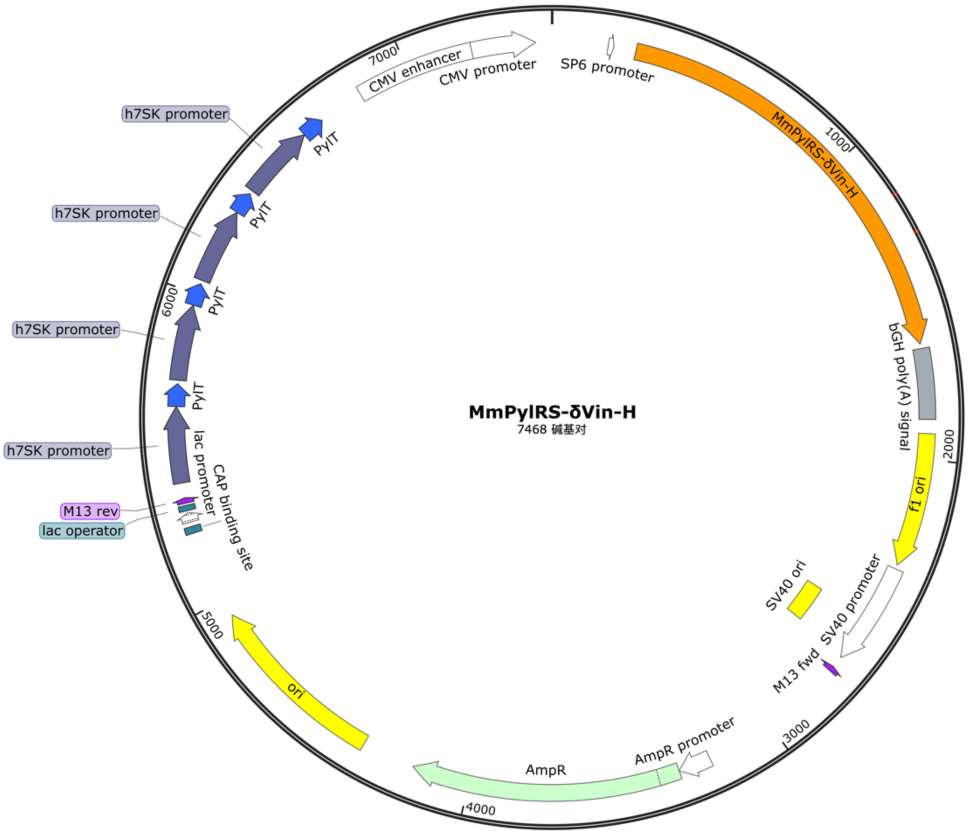

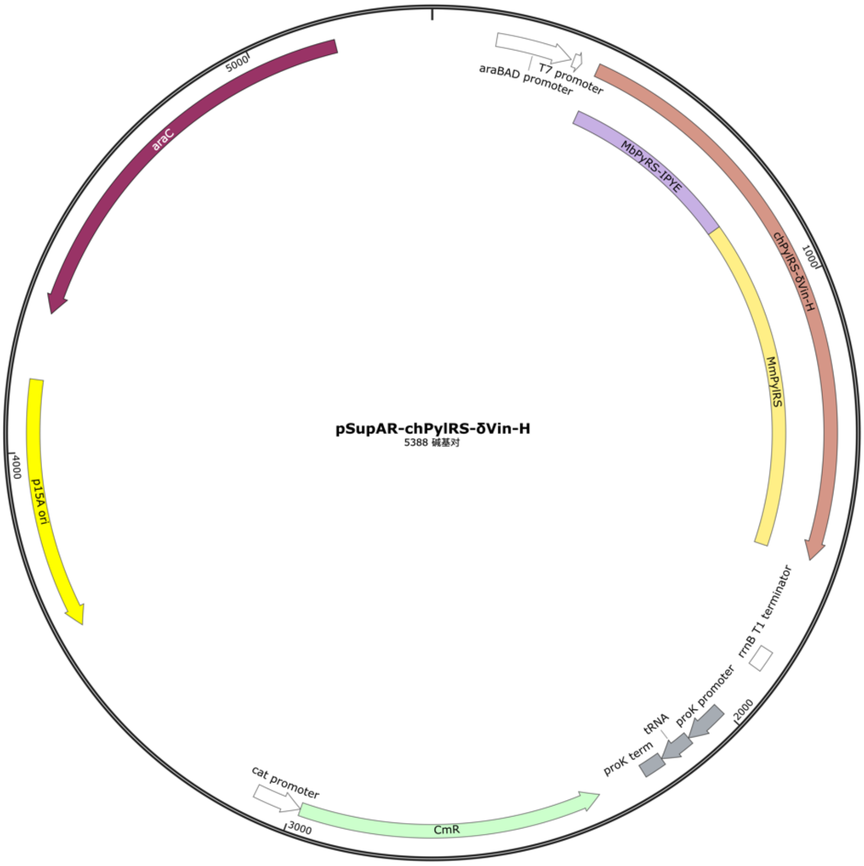

Supplementary Figure 12: Plasmid map of PylRS in the eukaryotic expression vector (top) and prokaryotic expression vector (bottom).

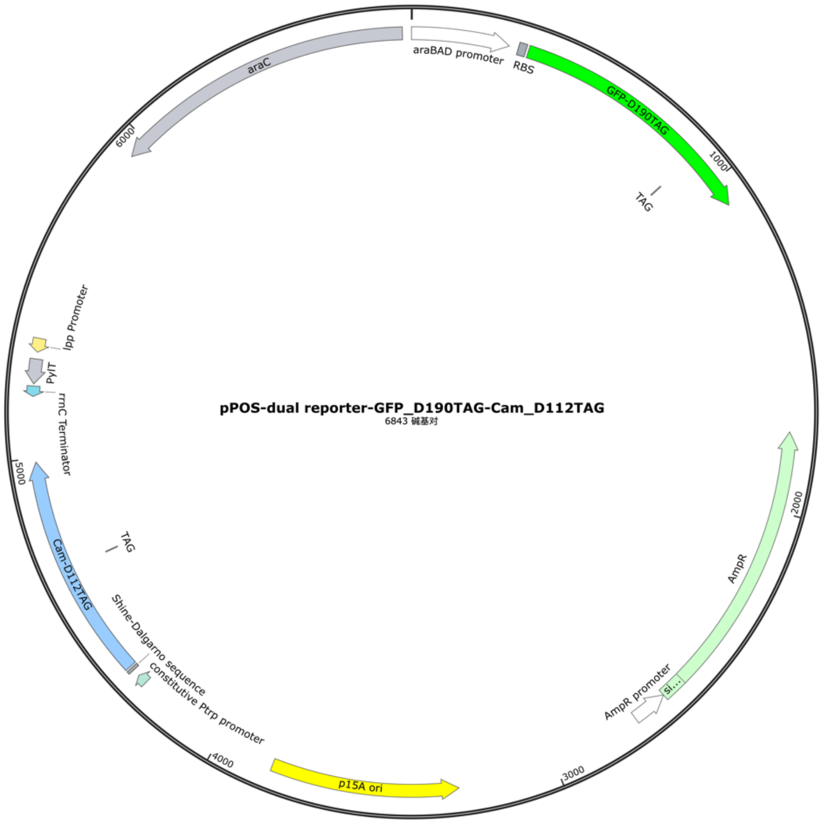

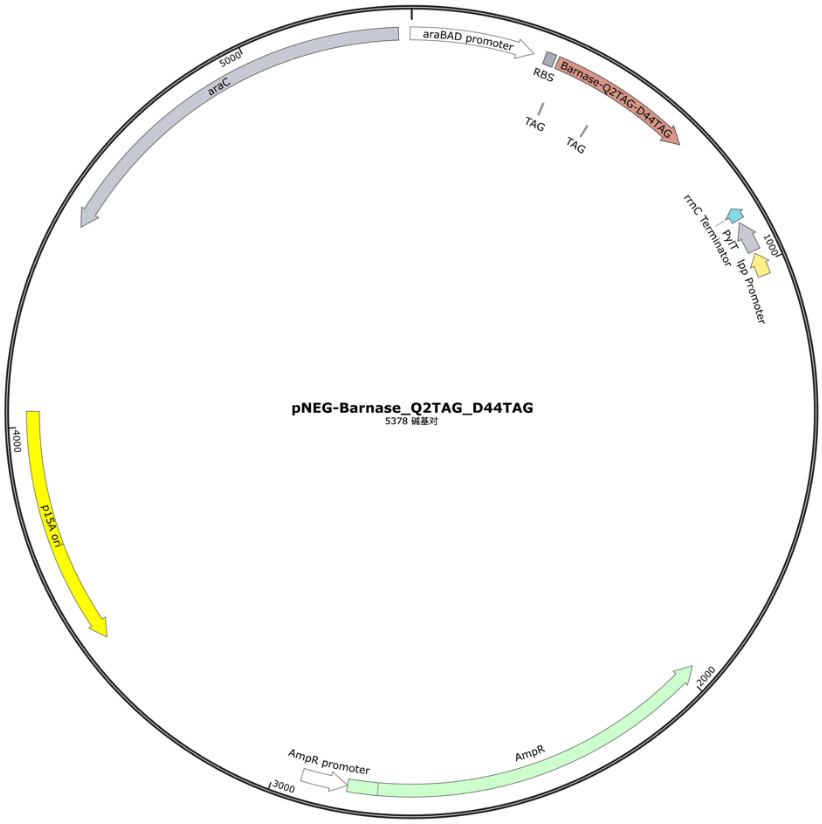

Supplementary Figure 13: Plasmid map of the positive selection plasmid (top) and negative selection plasmid (bottom).

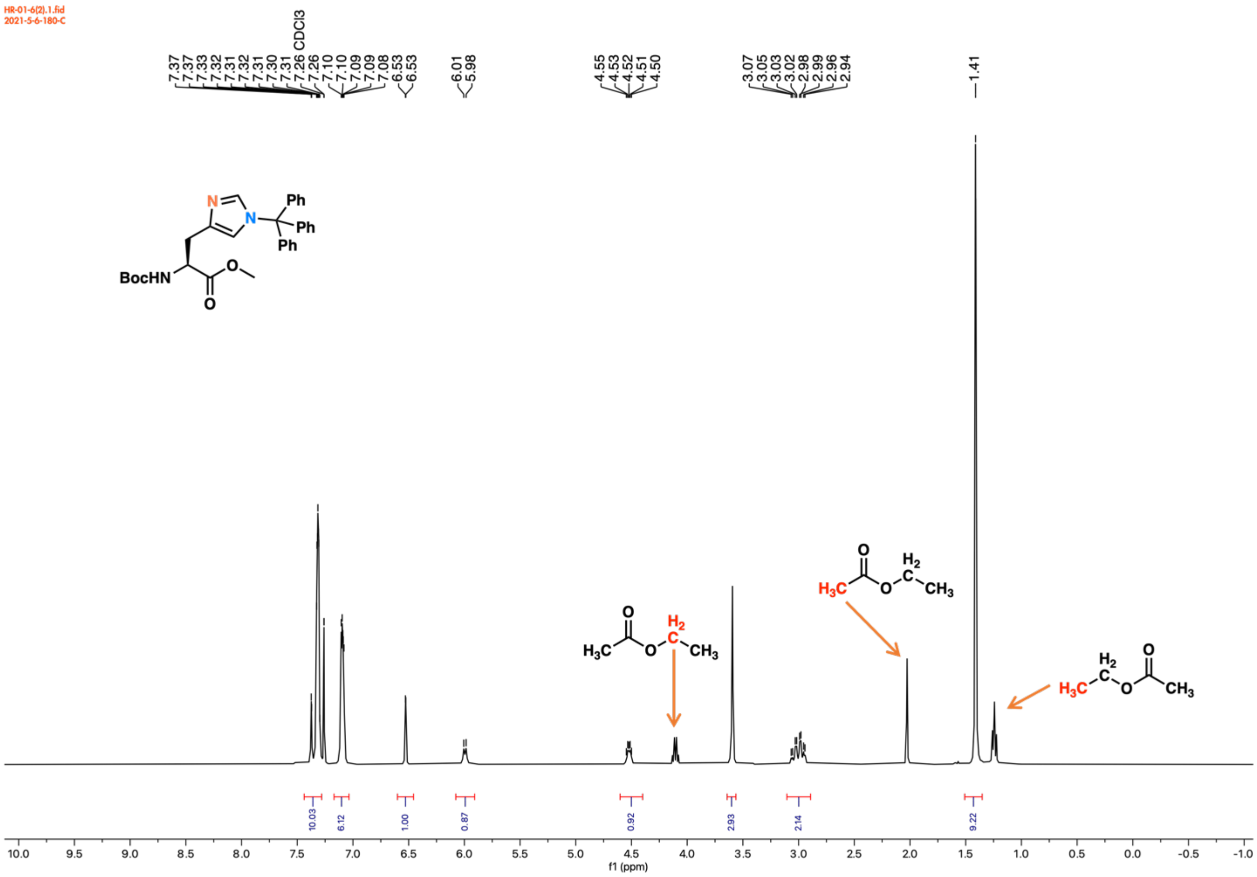

Supplementary Figure 14. ^{1}H NMR spectrum for compound **2**.

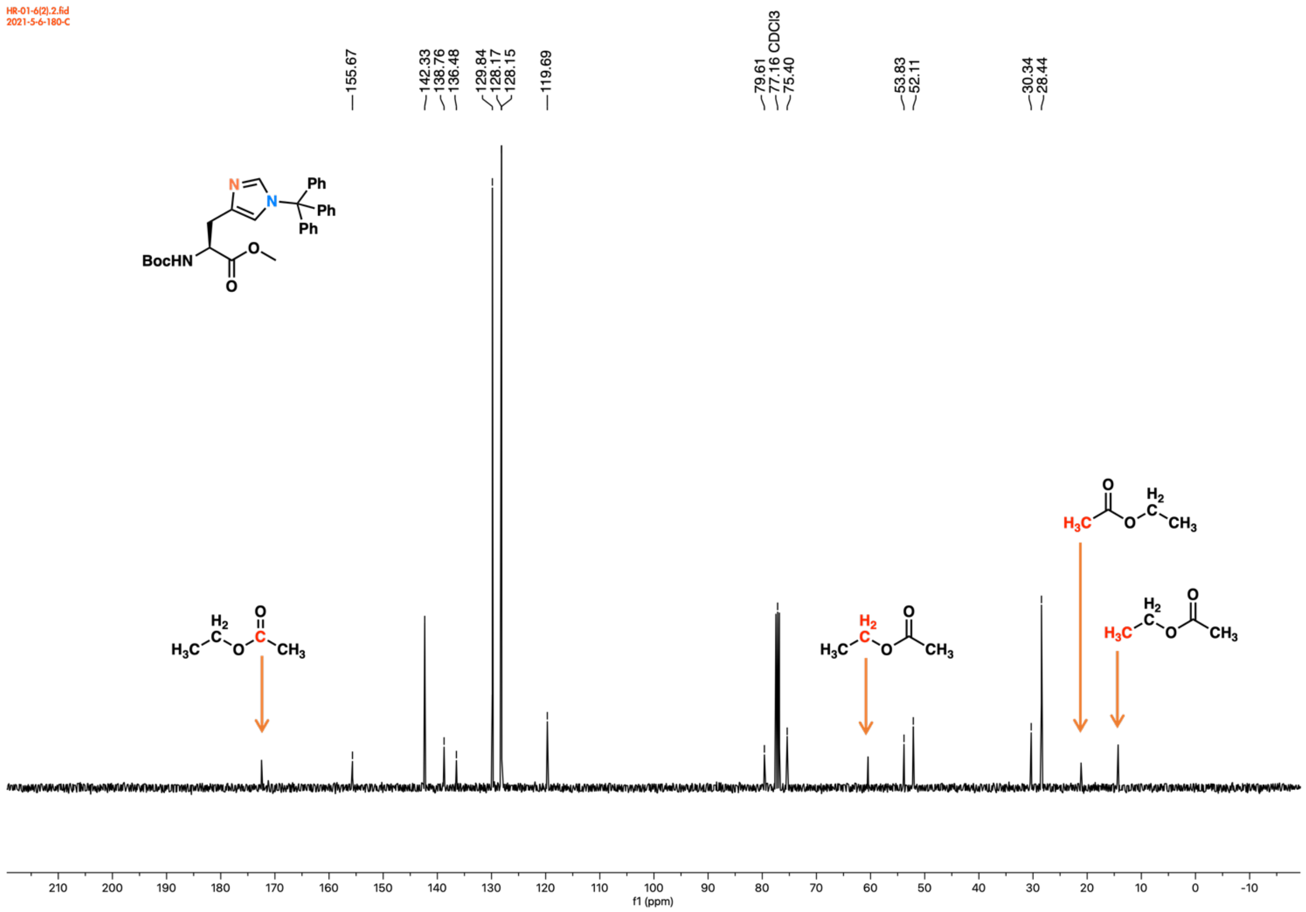

Supplementary Figure 15. ^{13}C NMR spectrum for compound **2**.

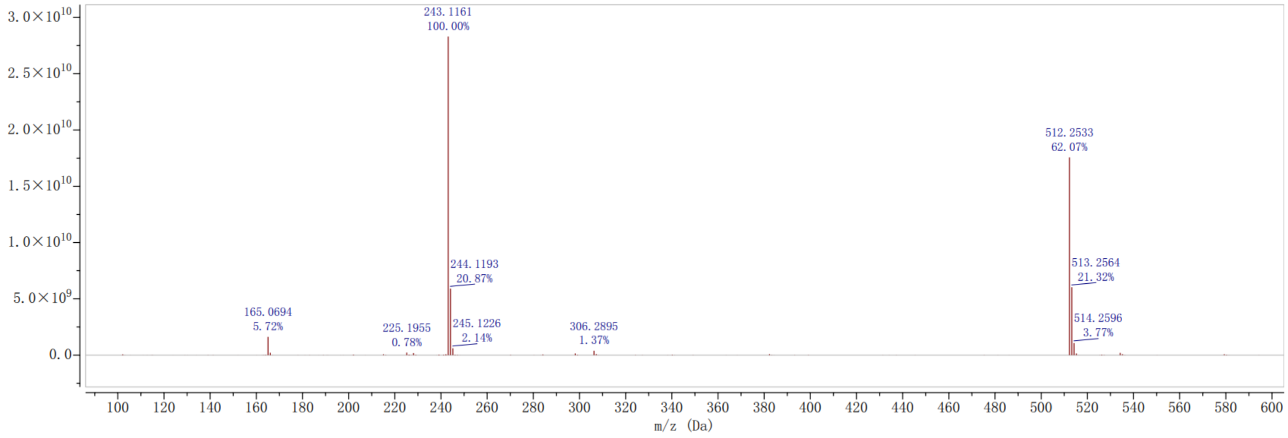

Supplementary Figure 16. High-resolution mass spectral of for compound **2**.

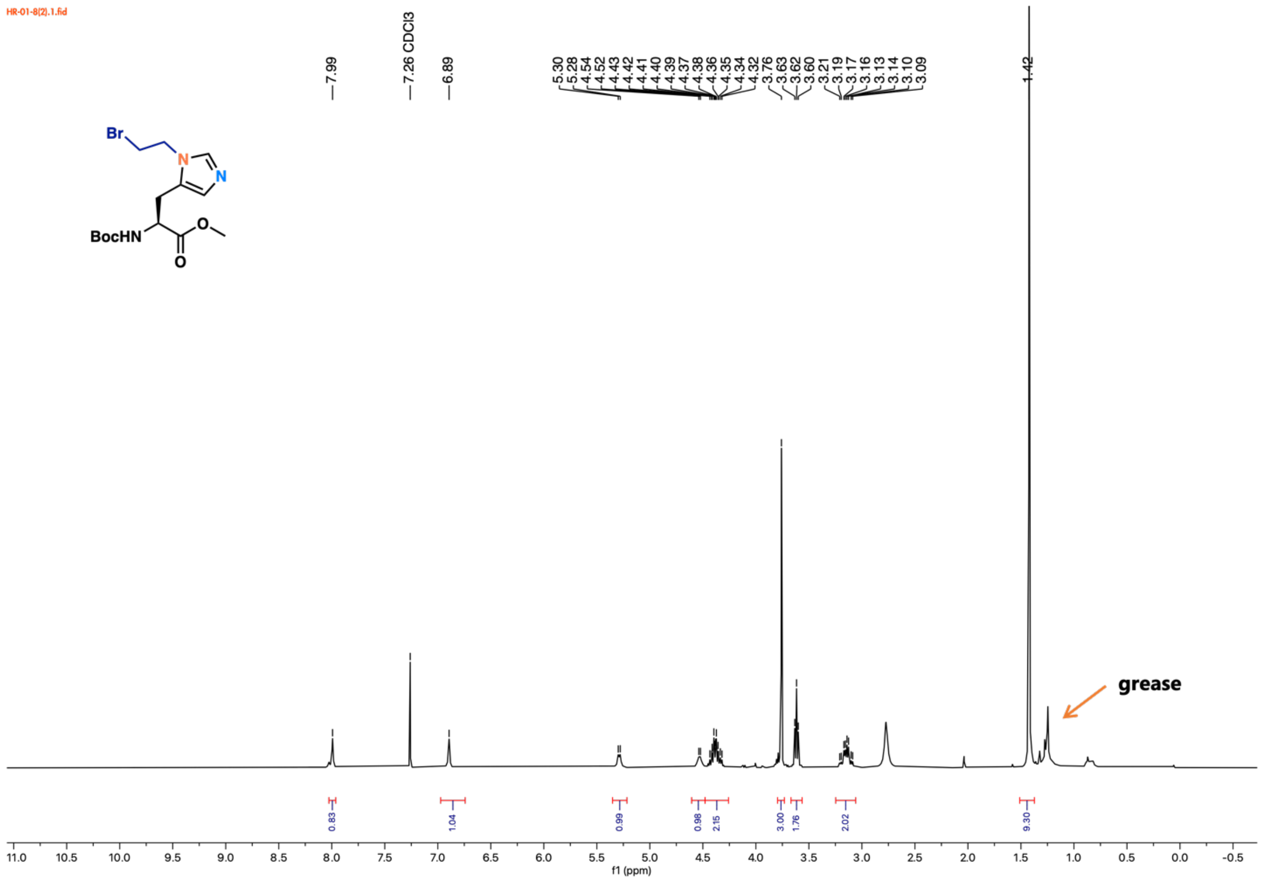

Supplementary Figure 17. ^{1}H NMR spectrum for compound **4**.

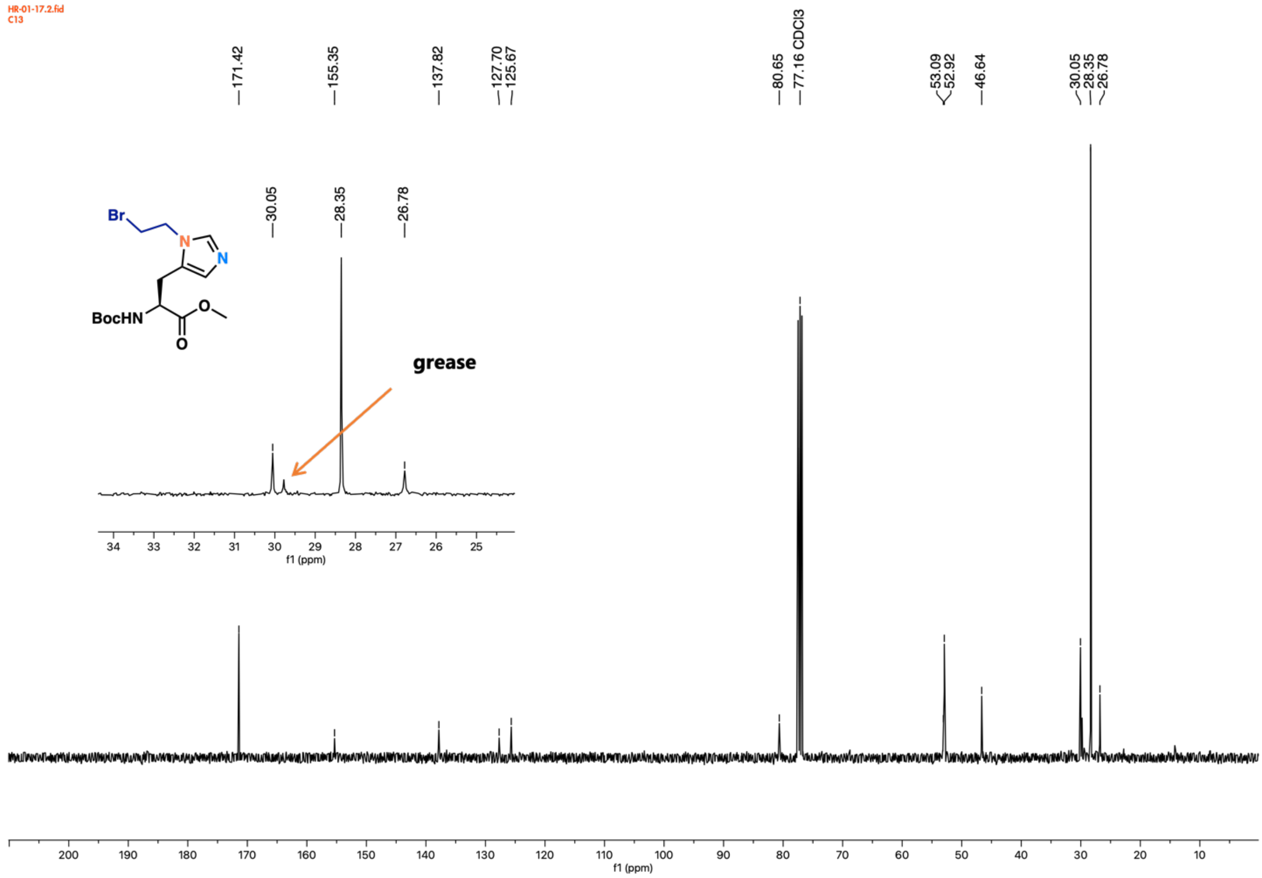

Supplementary Figure 18. ^{13}C NMR spectrum for compound **4**.

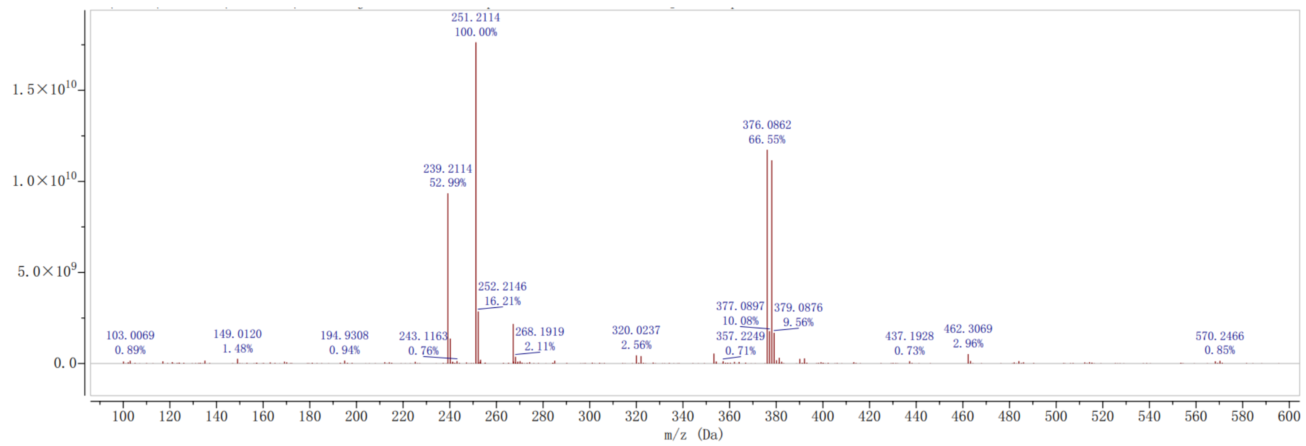

Supplementary Figure 19. High-resolution mass spectral of for compound **4**.

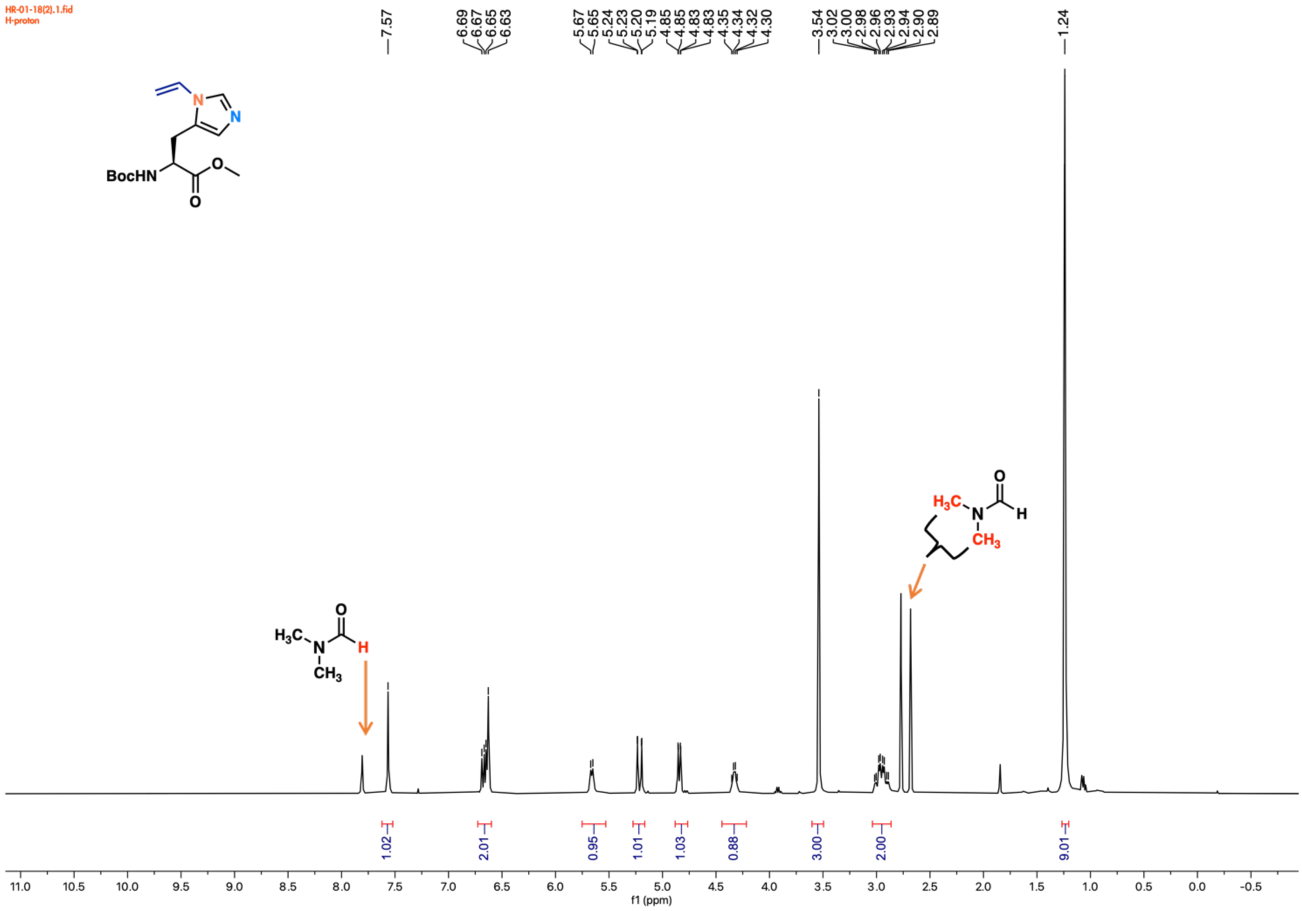

Supplementary Figure 20. ^{1}H NMR spectrum for compound **5**.

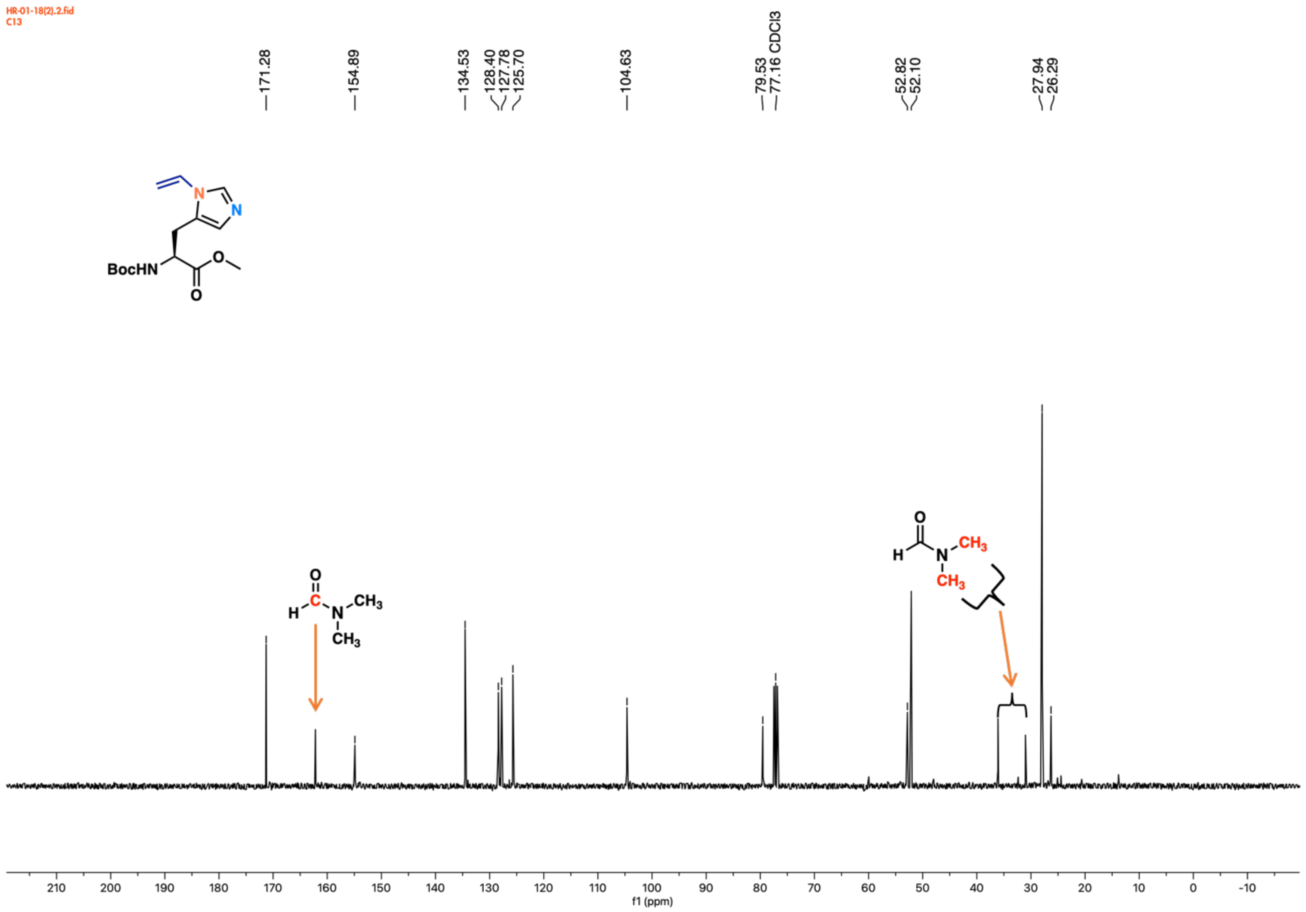

Supplementary Figure 21. ^{13}C NMR spectrum for compound **5**.

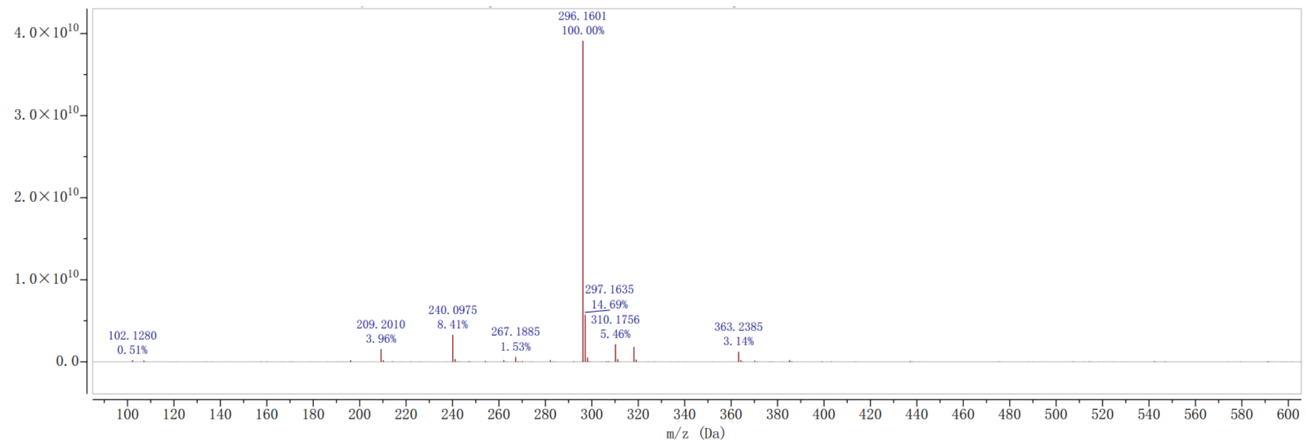

Supplementary Figure 22. High-resolution mass spectral of for compound **5**.

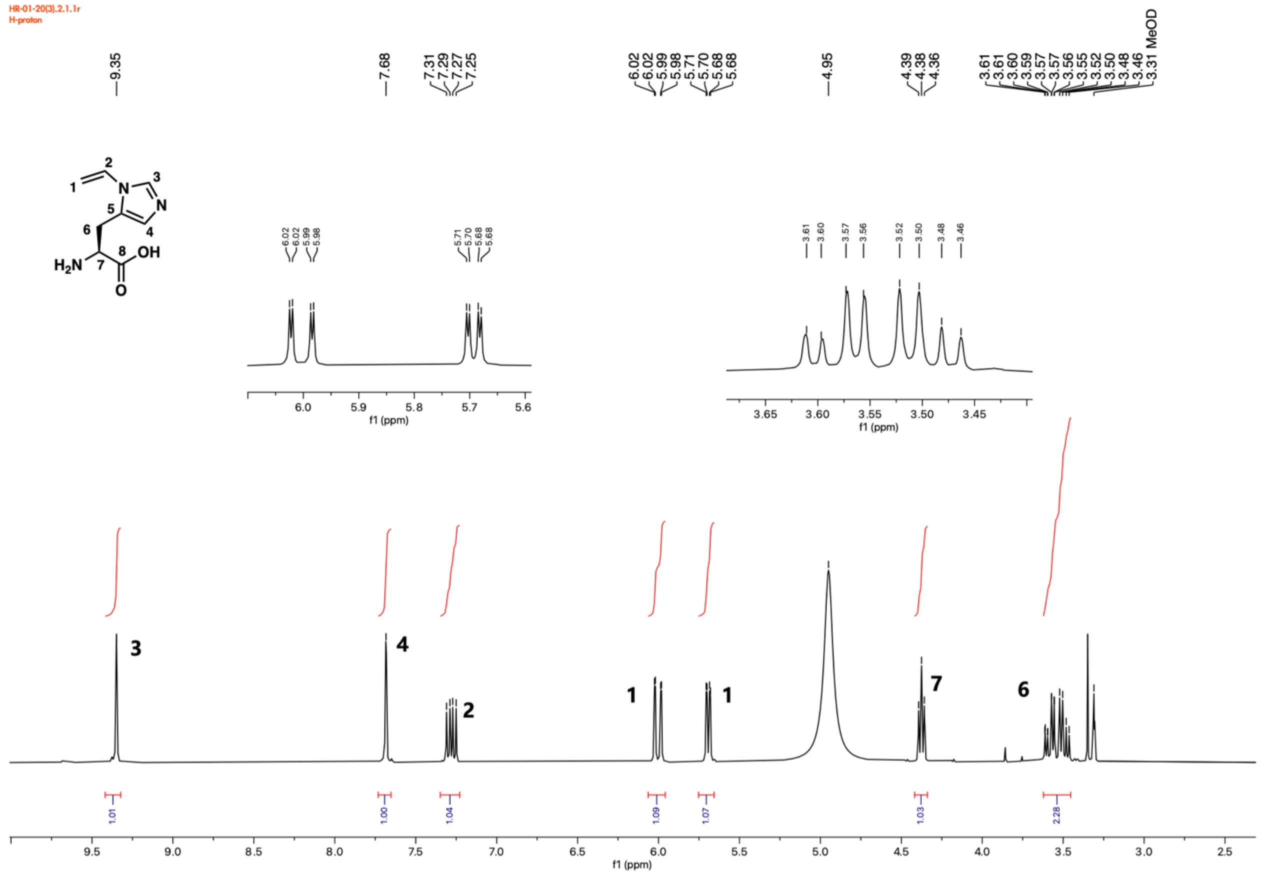

Supplementary Figure 23. ^{1}H NMR spectrum for compound **7**.

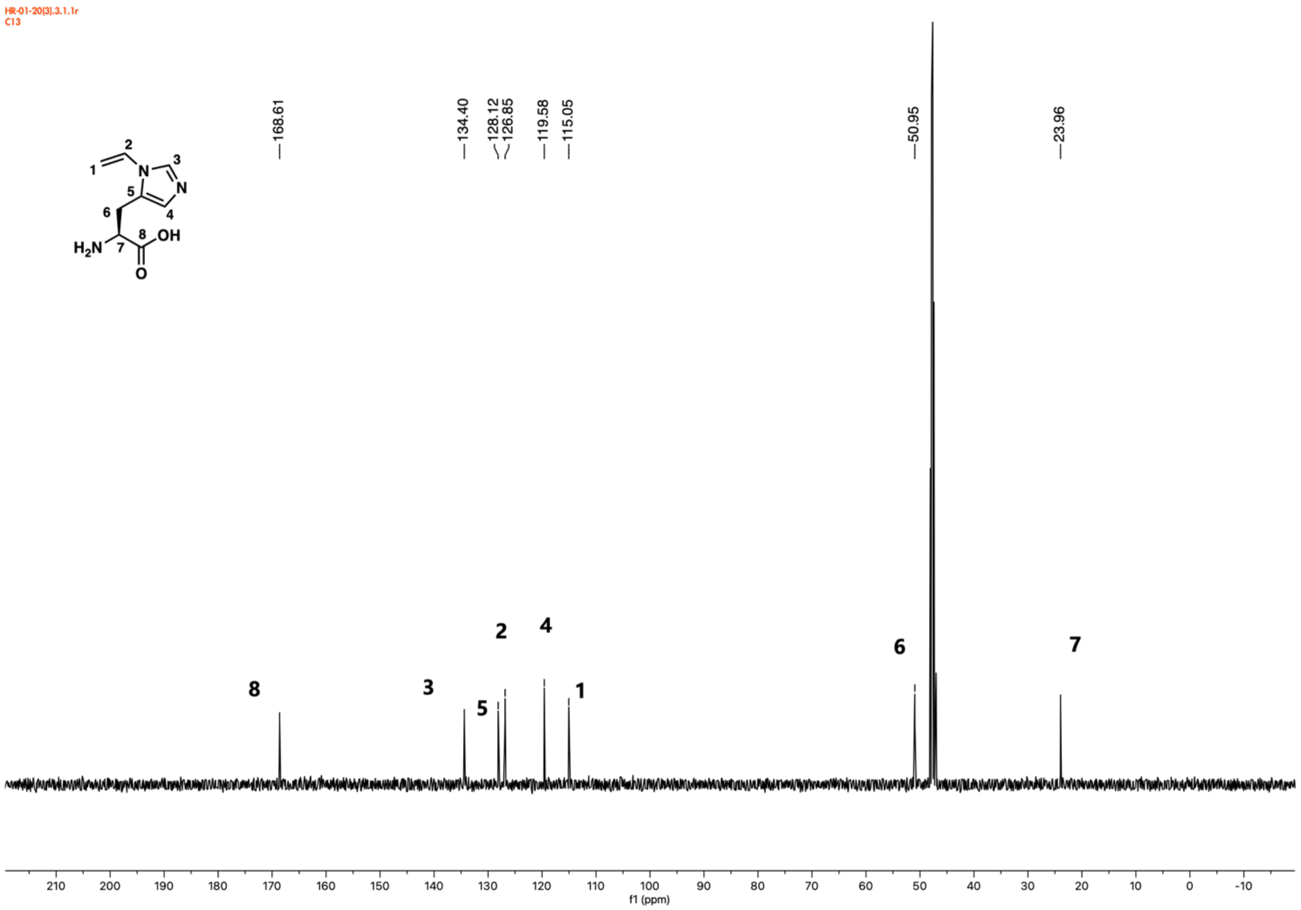

Supplementary Figure 24. ^{13}C NMR spectrum for compound **7.**

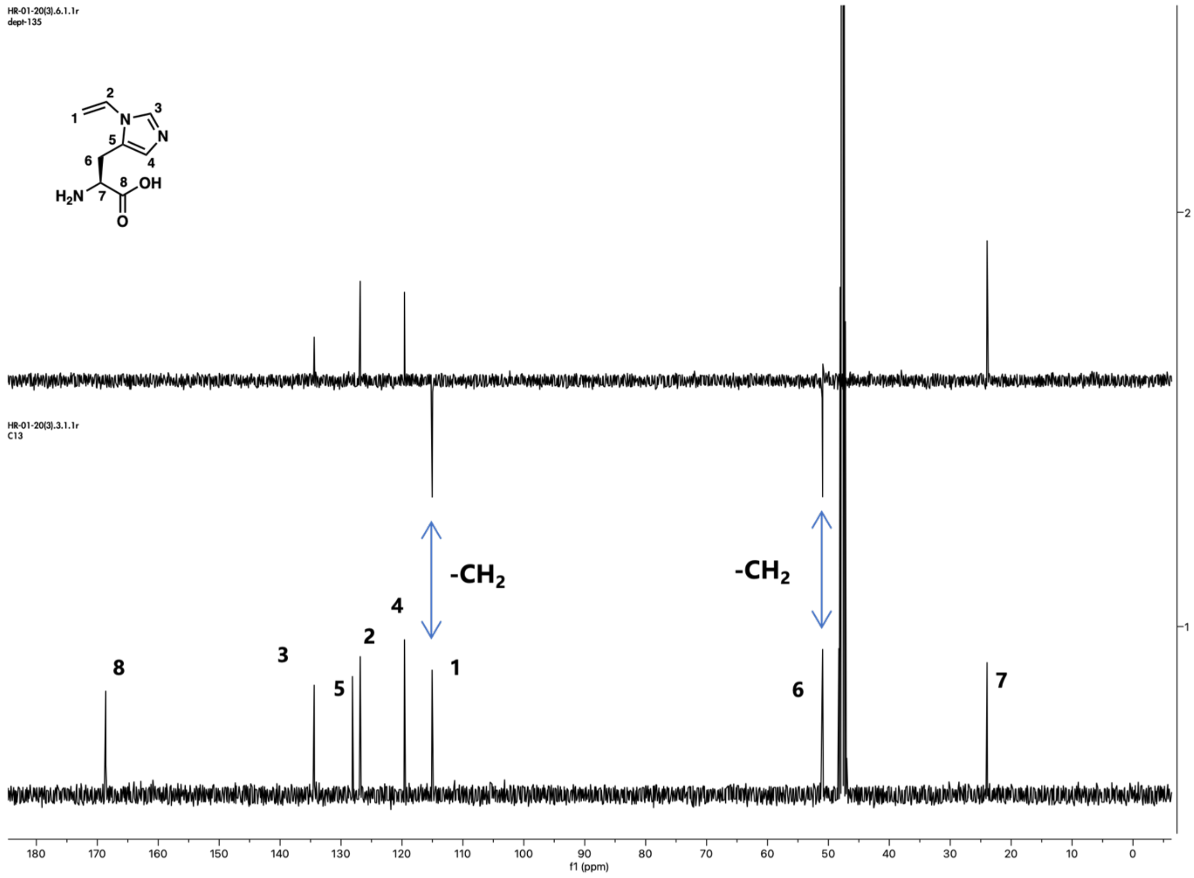

Supplementary Figure 25. ^{13}C NMR spectrum(top) and DEPT135 NMR spectrum(down) for compound **7**

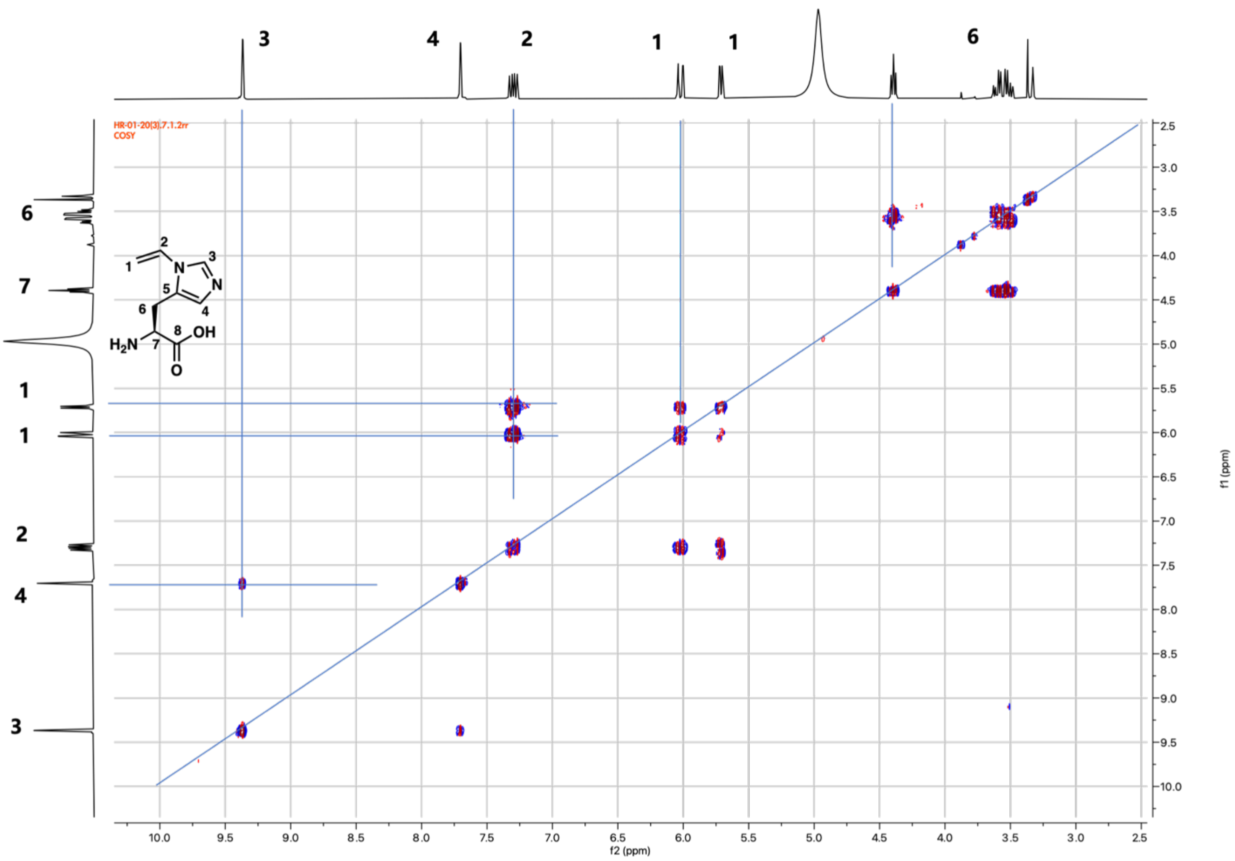

Supplementary Figure 26. HH COSY spectrum for compound **7.**

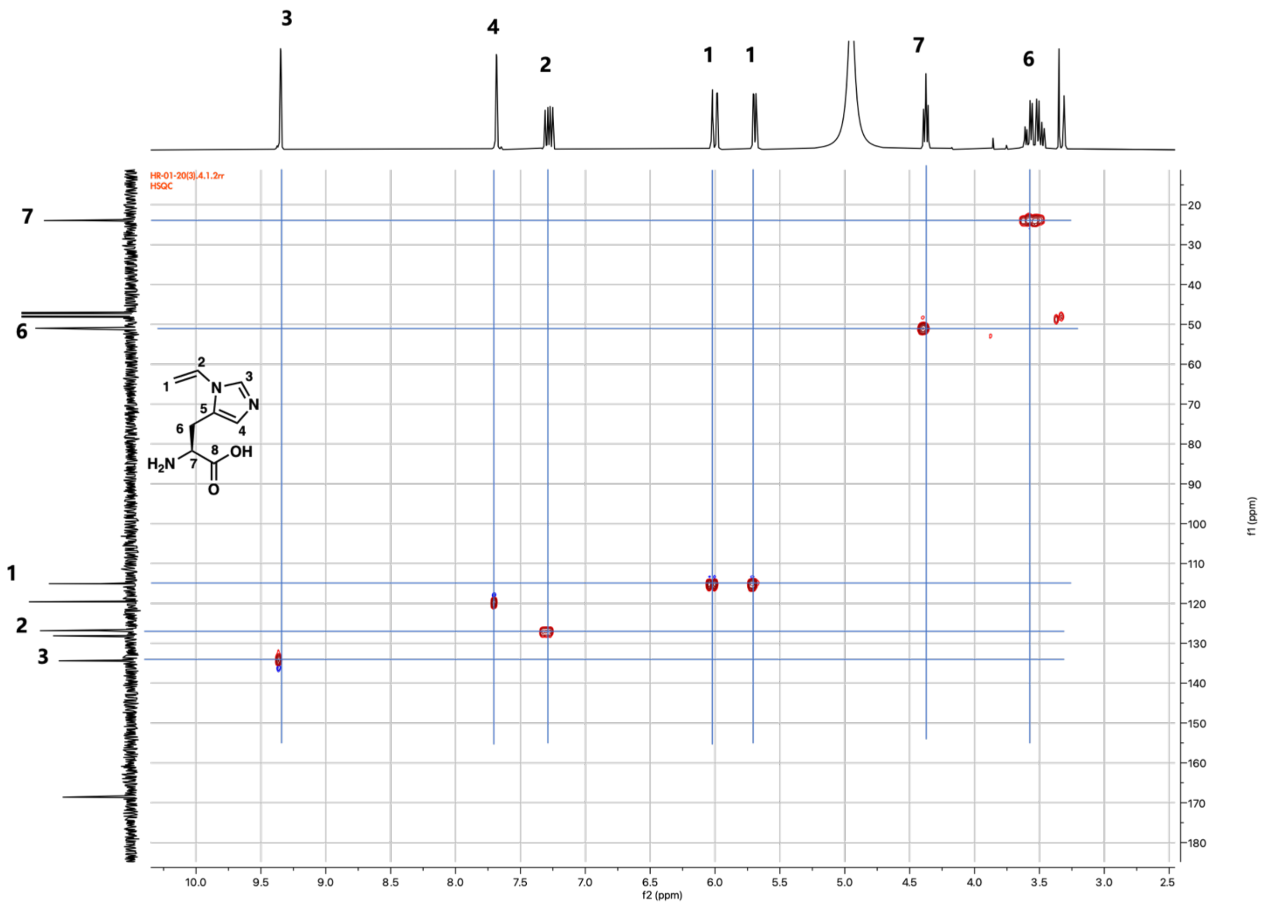

Supplementary Figure 27. HSQC spectrum for compound **7**

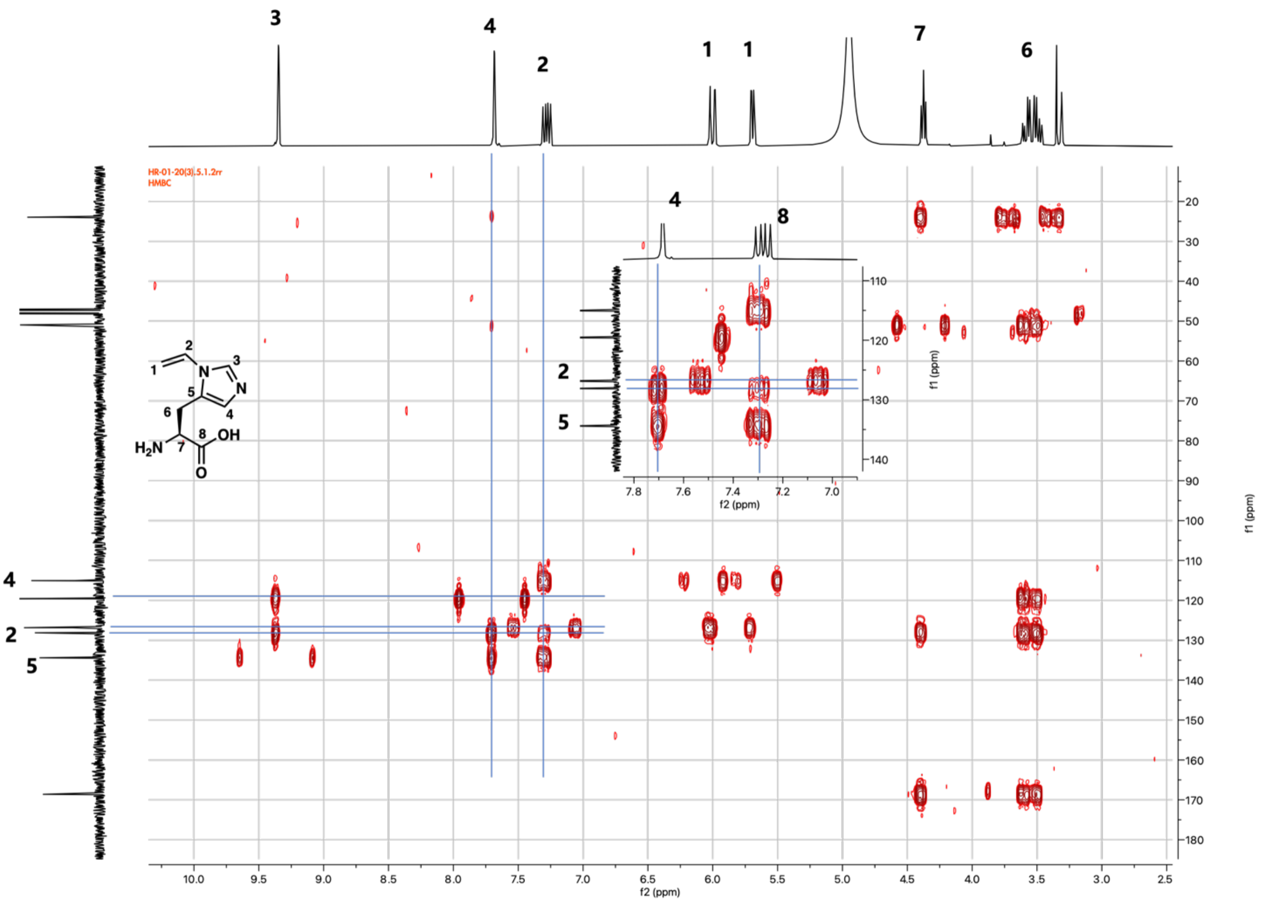

Supplementary Figure 28: HMBC NMR spectrum for compound **7**

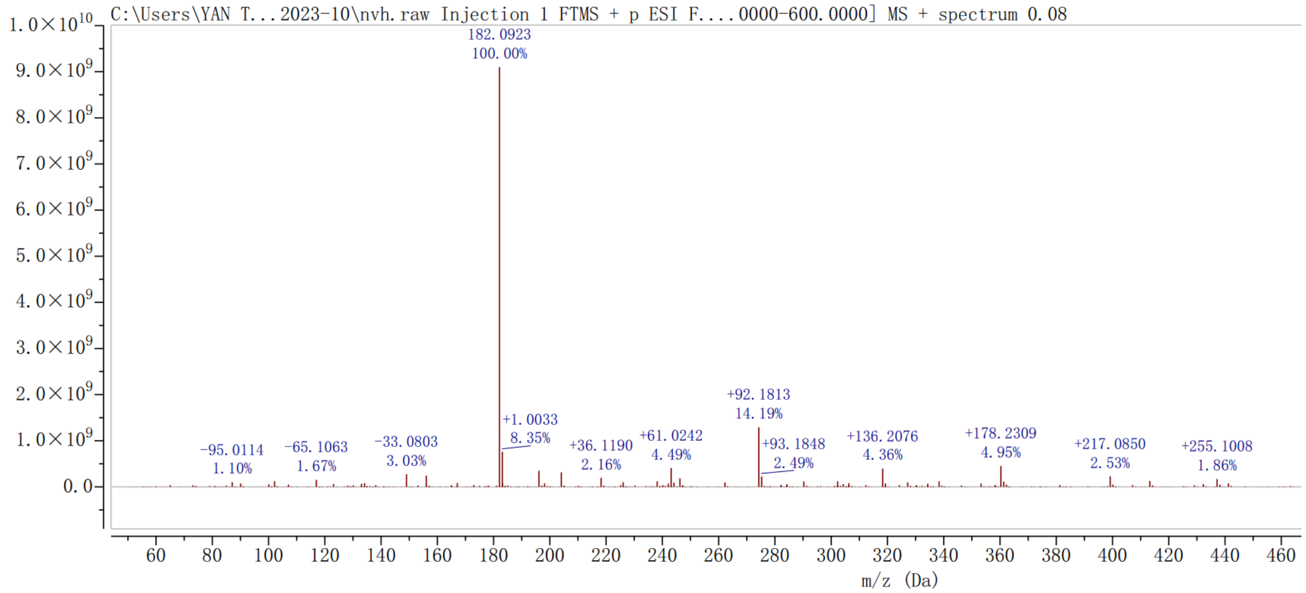

Supplementary Figure 29: High-resolution mass spectrum for **7.**

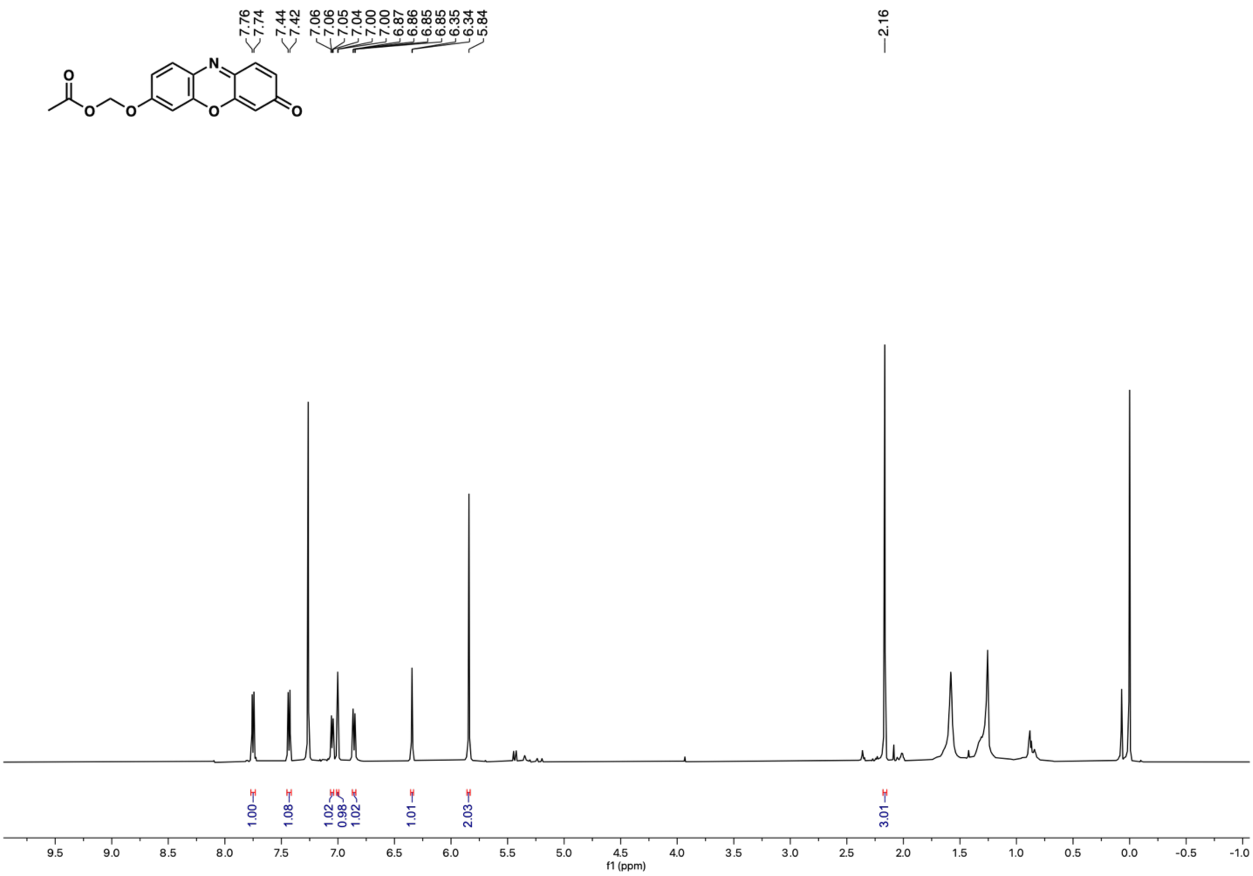

Supplementary Figure 30: ^{1}H NMR spectrum for A-Me-Res.

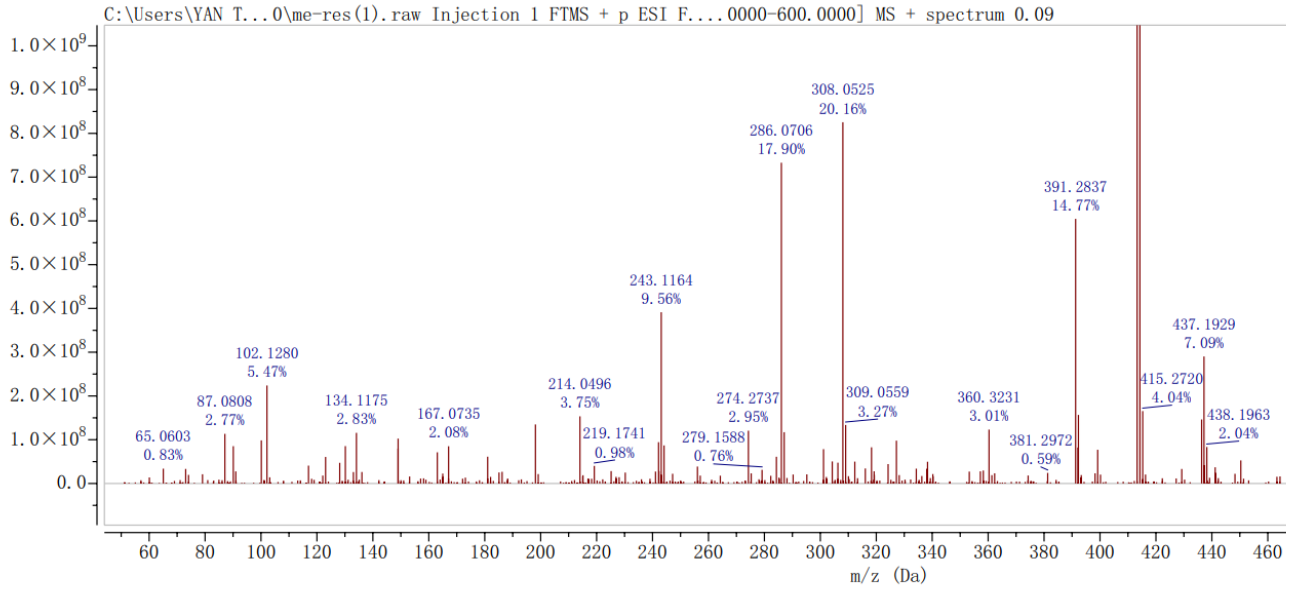

Supplementary Figure 31: High-resolution mass spectrum for A-Me-Res..

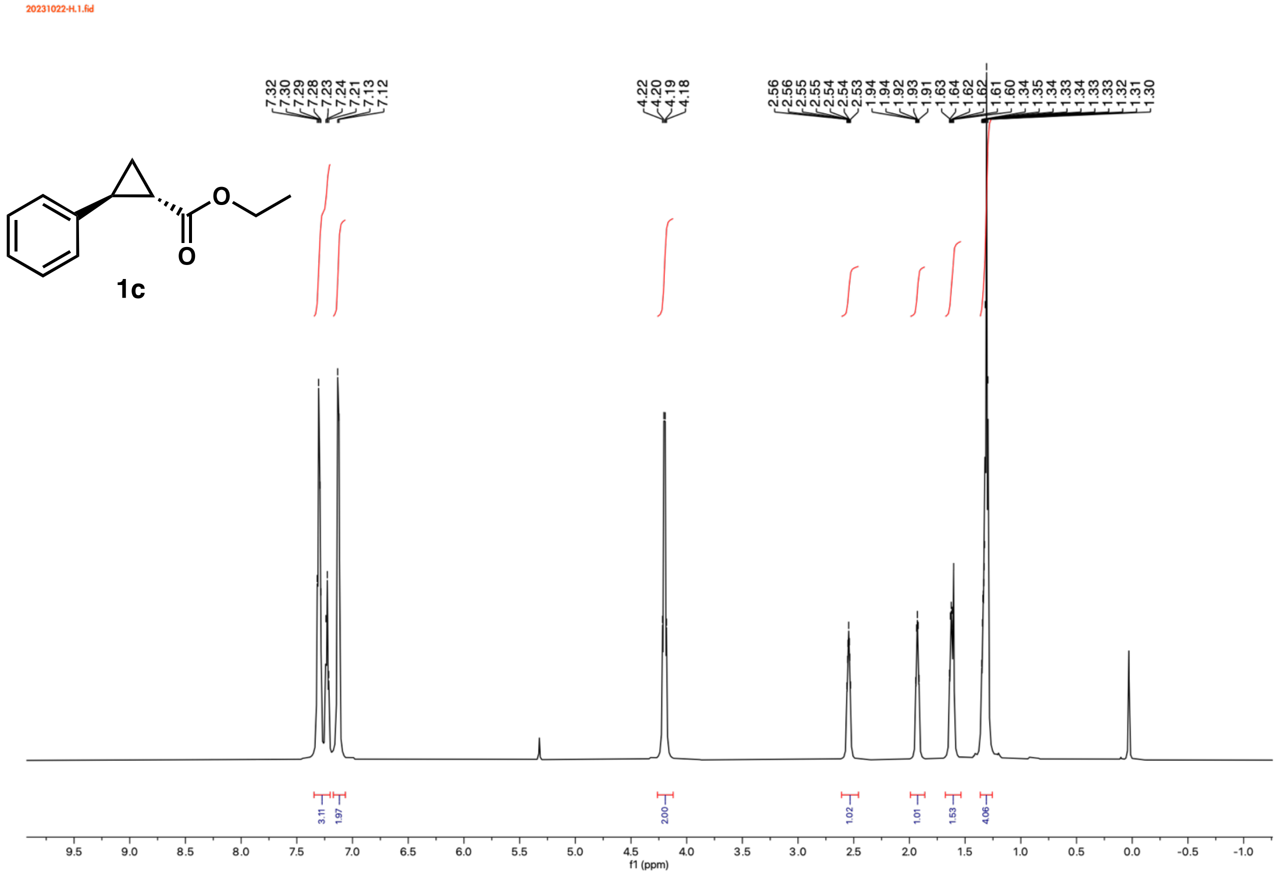

Supplementary Figure 32: ^{1}H NMR spectrum for Mb*-H93δVinH catalyzed product **1c**.

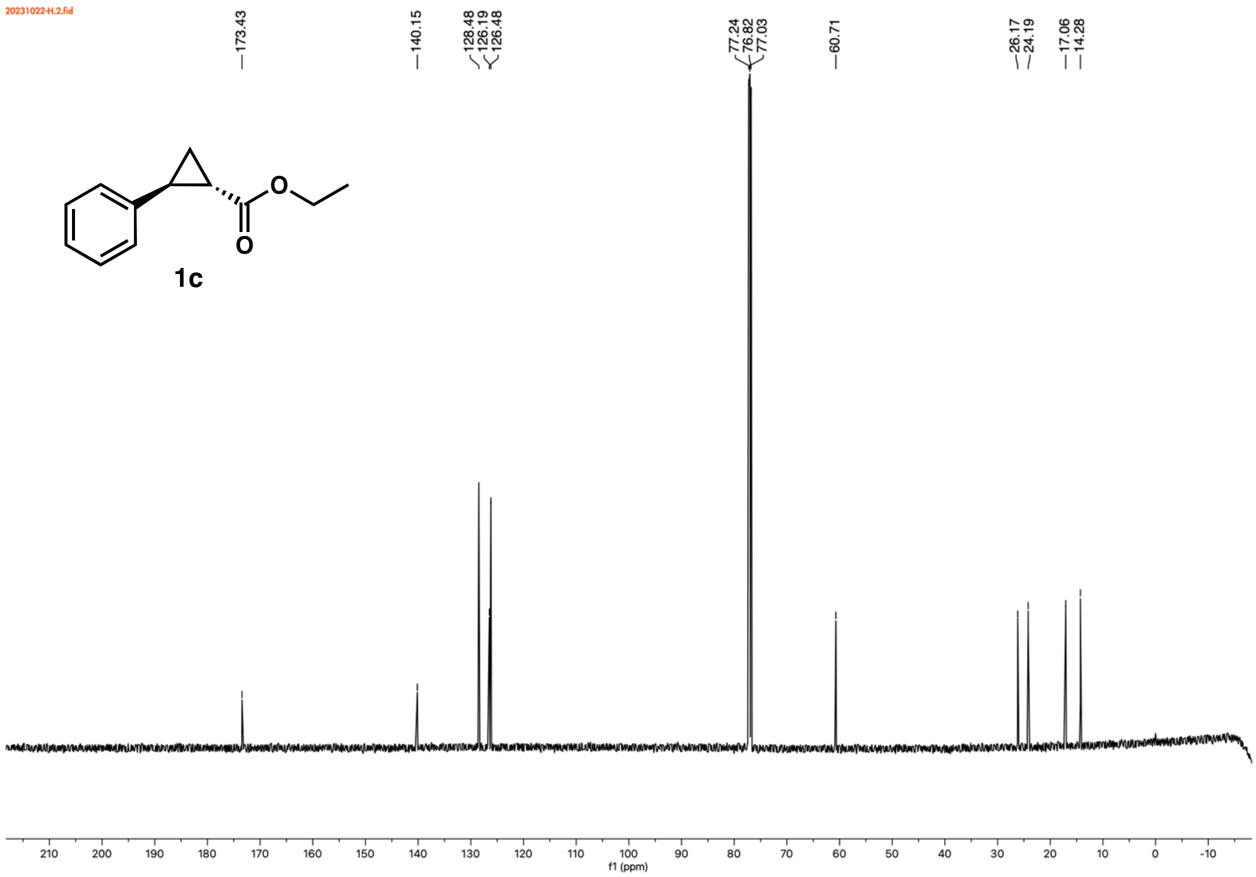

Supplementary Figure 33: ^{13}C NMR spectrum for Mb*-H93δVinH catalyzed product **1c**.

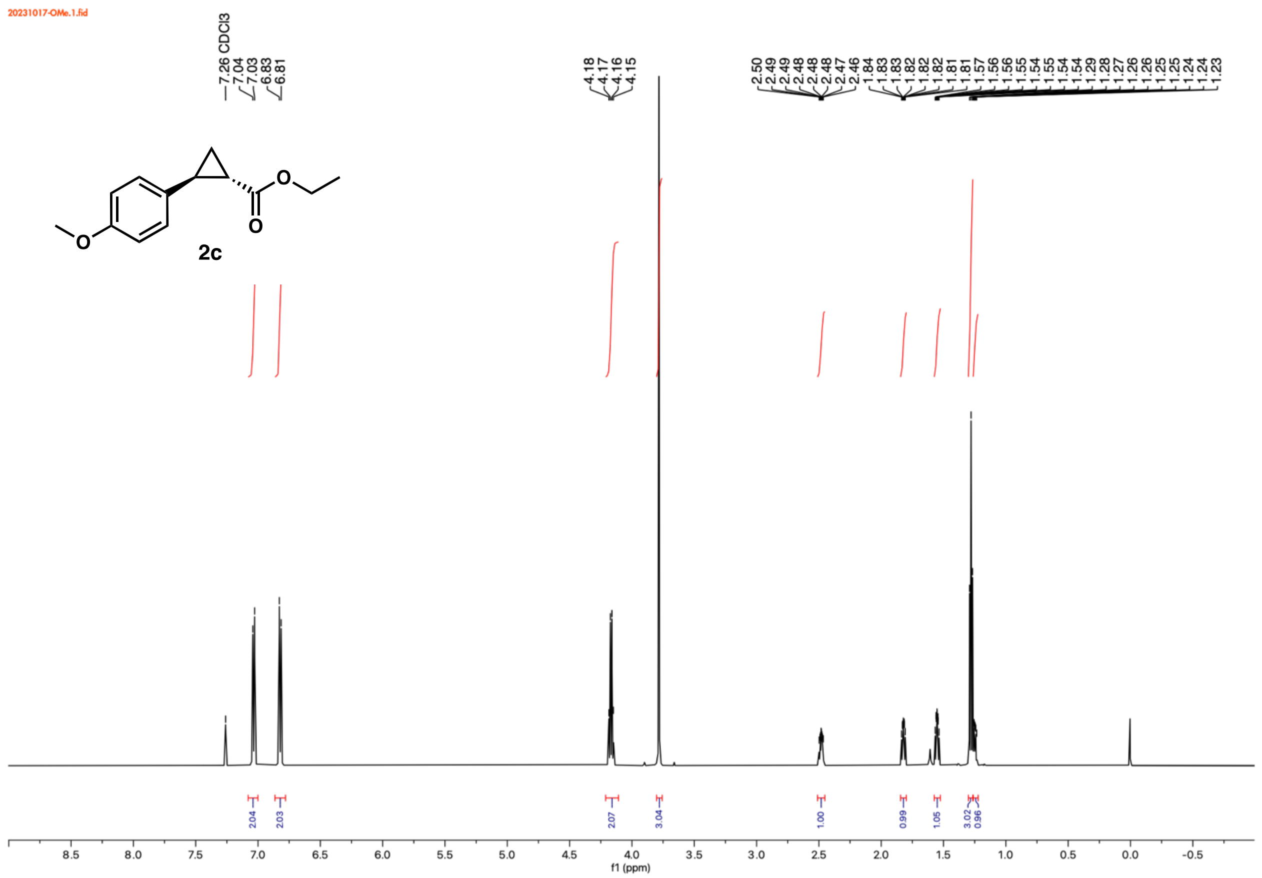

Supplementary Figure 34: ^{1}H NMR spectrum for Mb*-H93δVinH catalyzed product **2c**.

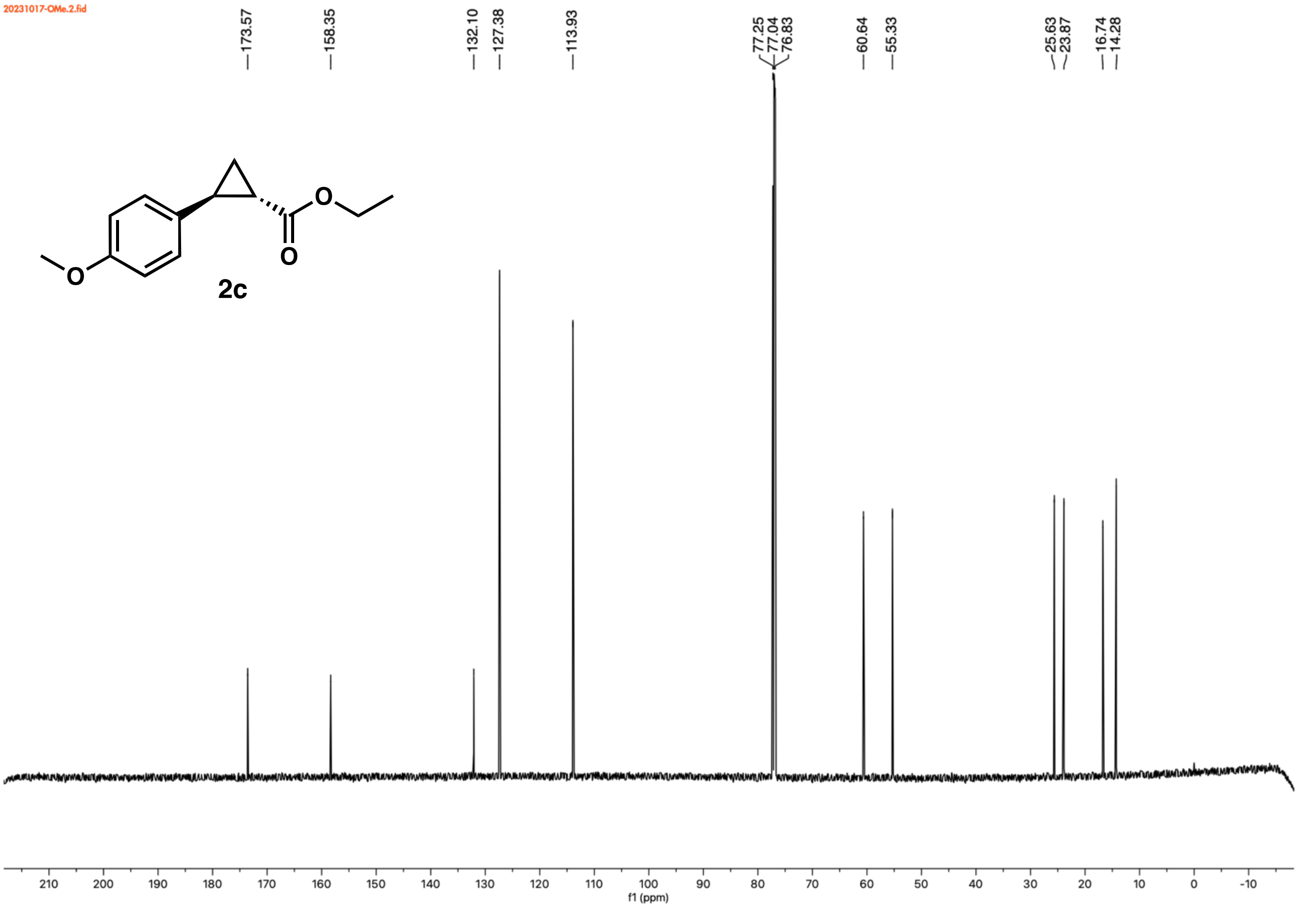

Supplementary Figure 35: ^{13}C NMR spectrum for Mb*-H93δVinH catalyzed product **2c**.

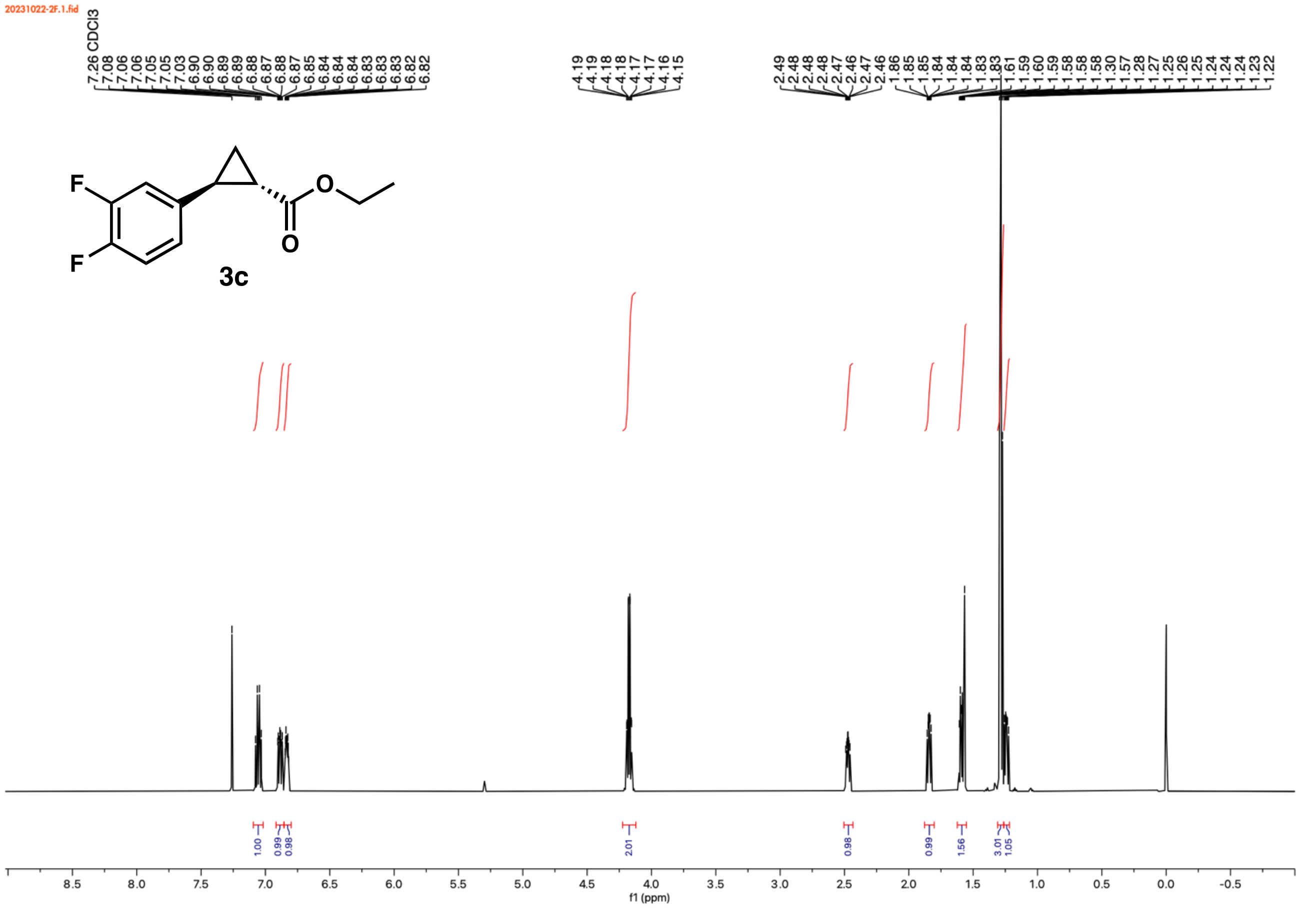

Supplementary Figure 36: ^{1}H NMR spectrum for Mb*-H93δVinH catalyzed product **3c**.

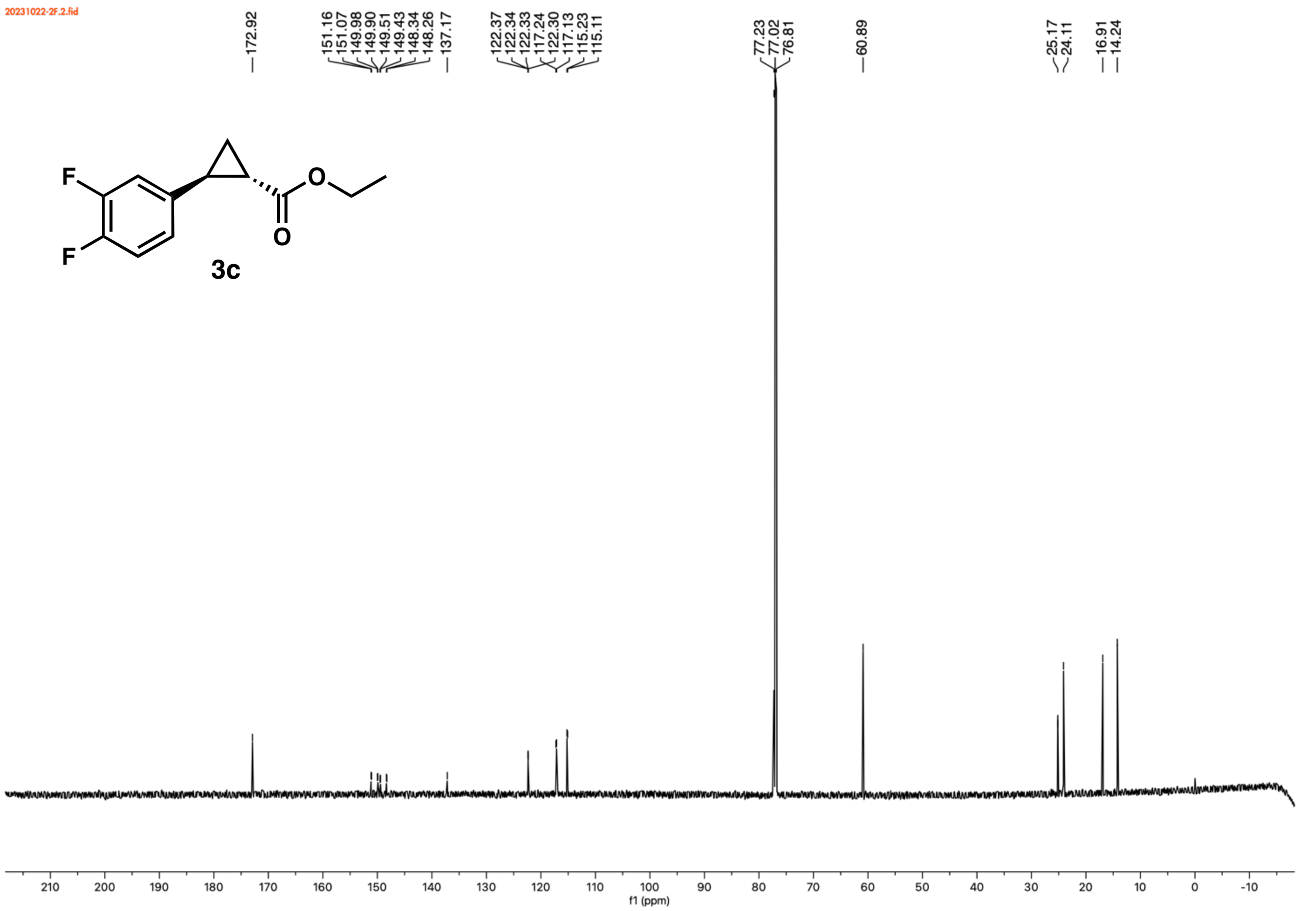

Supplementary Figure 37: ^{13}C NMR spectrum for Mb*-H93δVinH catalyzed product **3c**.

20231105-2F.1.fid

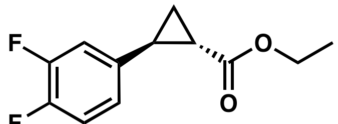

3c

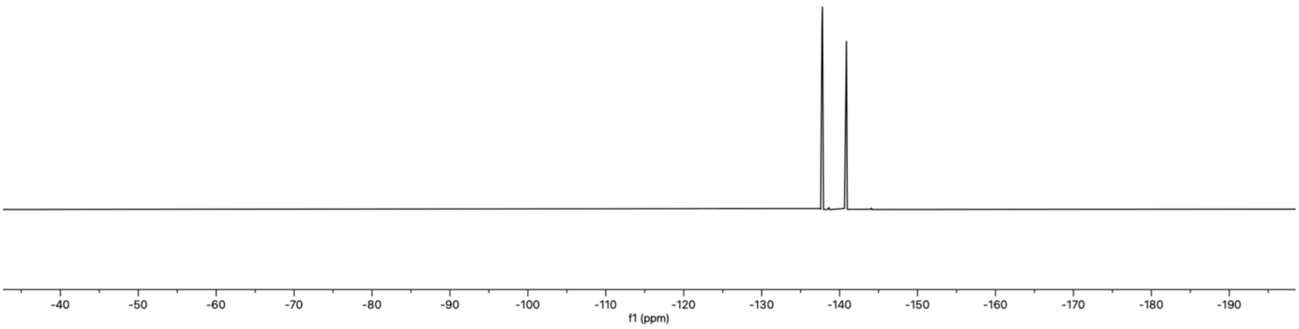

Supplementary Figure 38: ^{19}F NMR spectrum for Mb*-H93δVinH catalyzed product **3c**.

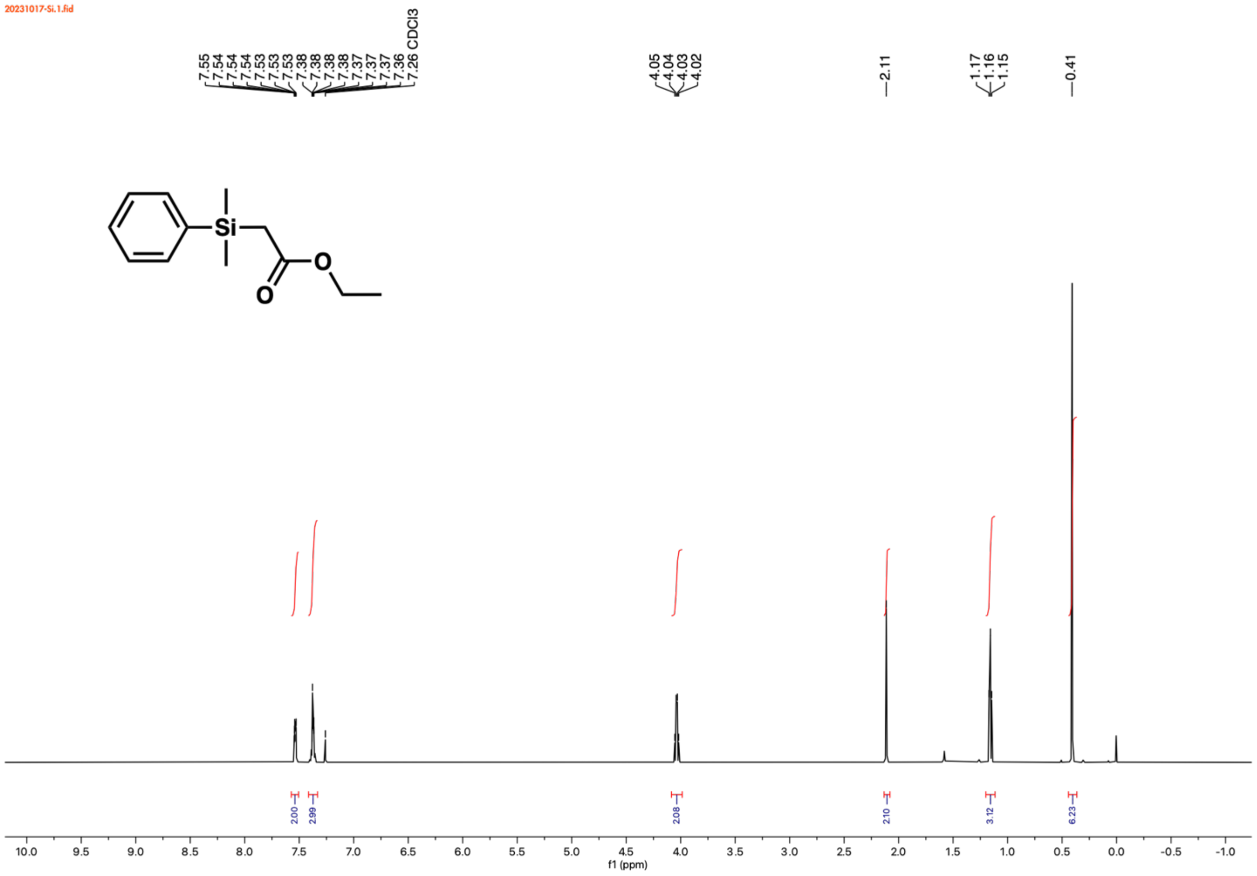

Supplementary Figure 39: ^{1}H NMR spectrum for Mb*-H93δVinH catalyzed product **4c**.

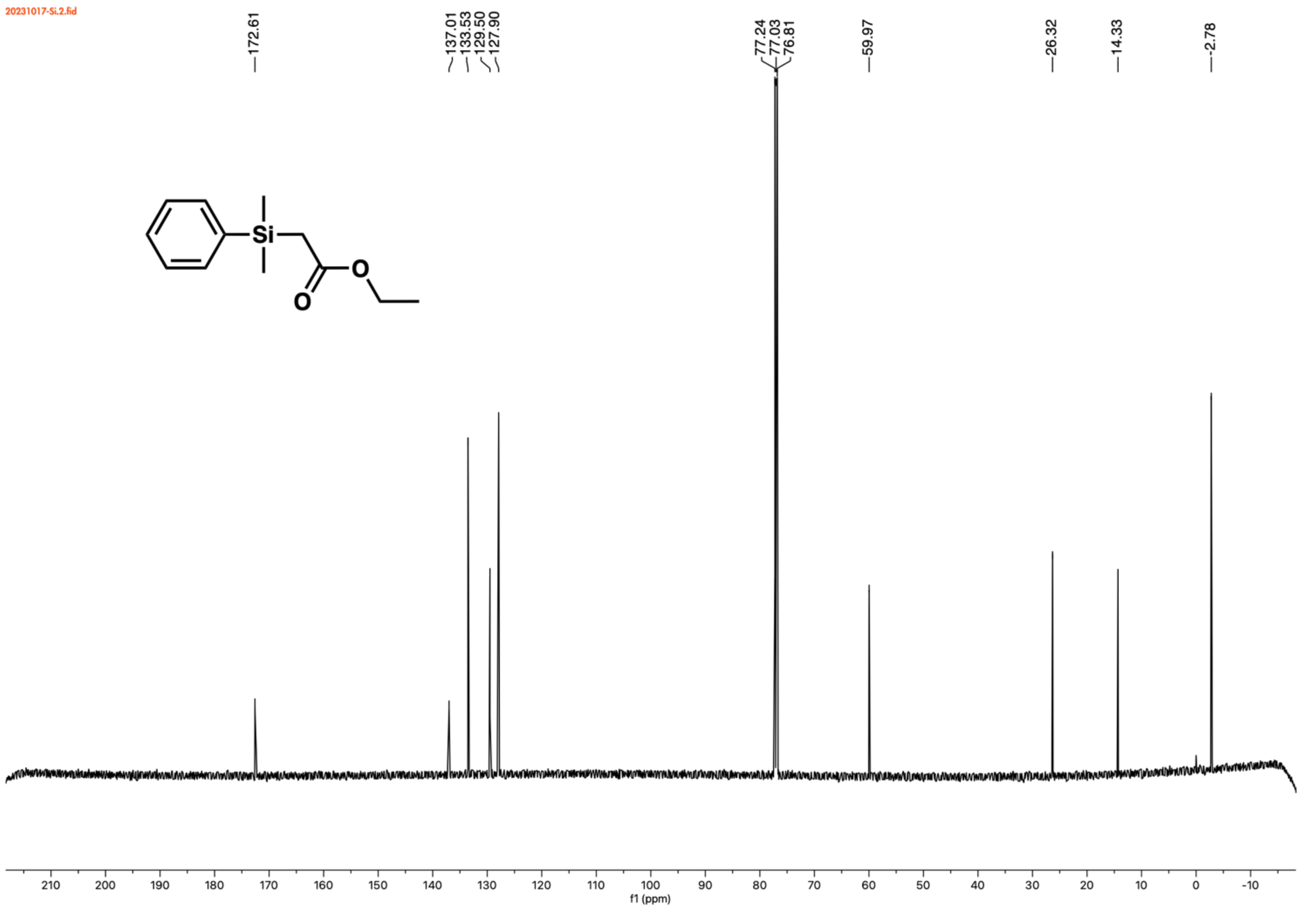

Supplementary Figure 40: ^{13}C NMR spectrum for Mb*-H93δVinH catalyzed product **4c**.

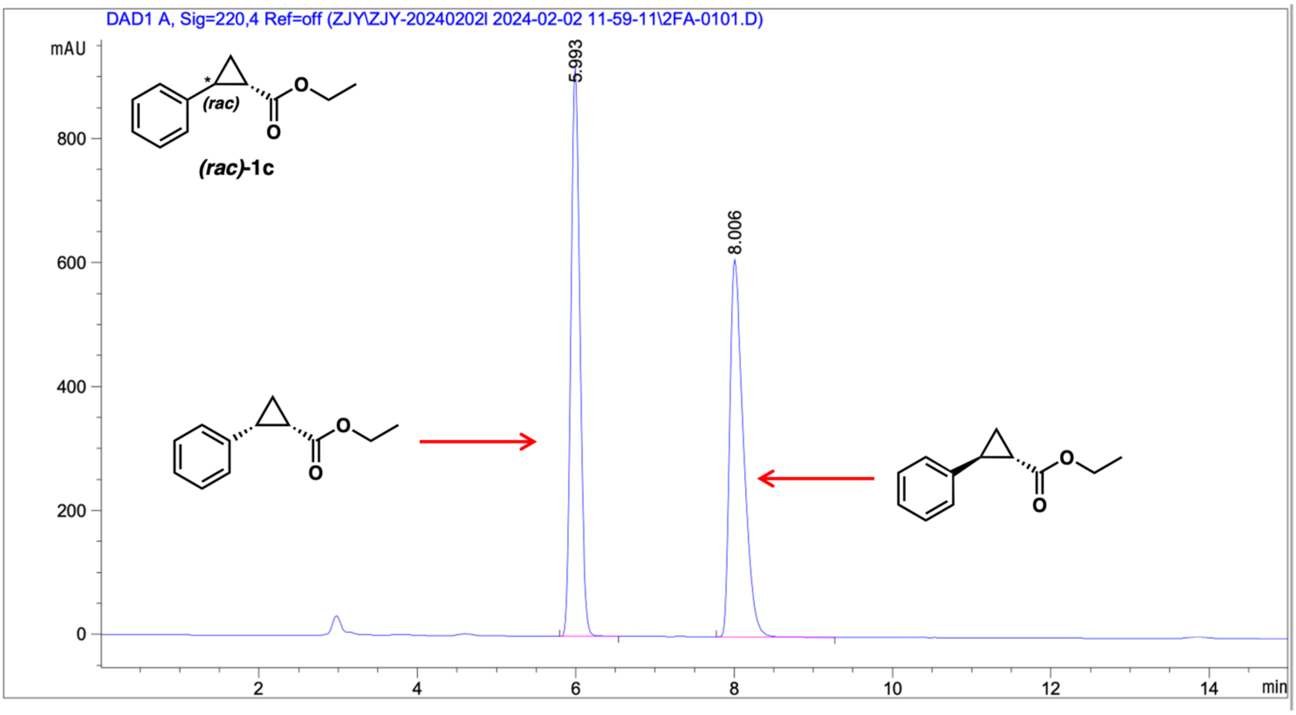

Peak #	RetTime [min]	Type	Width [min]	Area [mAU*s]	Height [mAU]	Area %
1	5.993	BB	0.1191	7157.60986	917.05573	49.3937
2	8.006	BB	0.1806	7333.32031	608.40643	50.6063

Supplementary Figure 41: chiral HPLC spectrum for ***(rac)*-1c**.

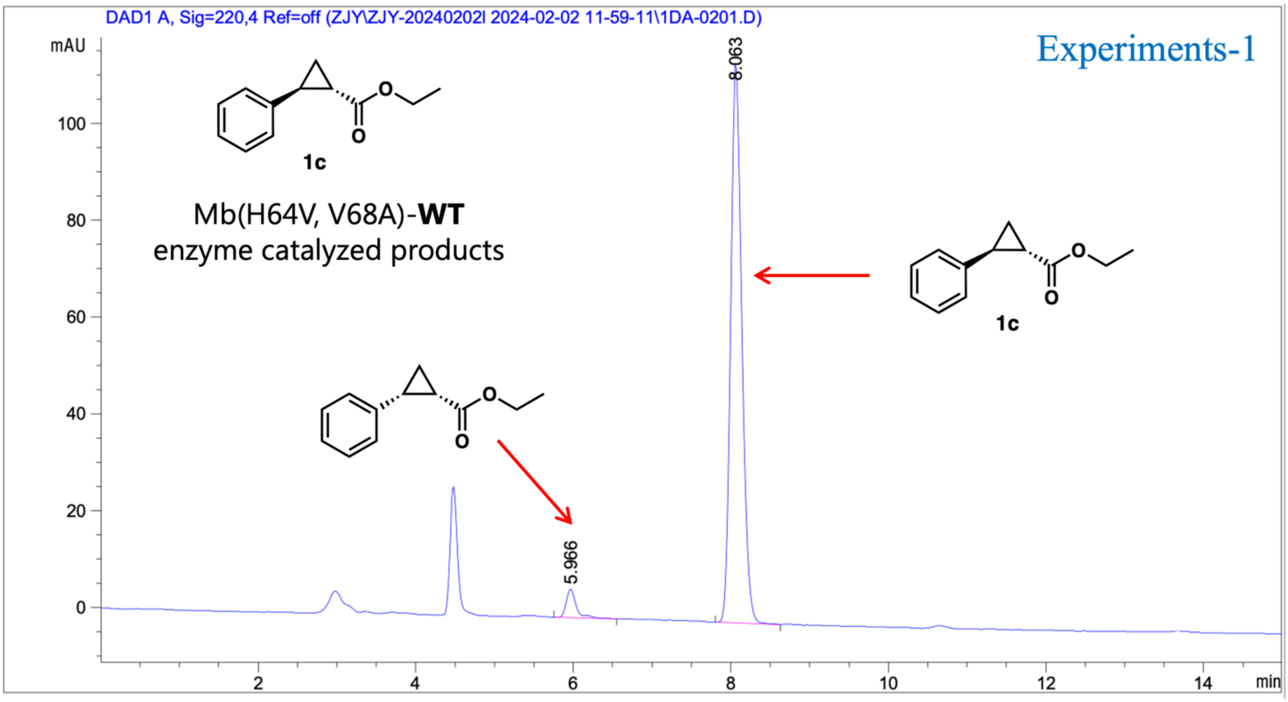

Peak #	RetTime [min]	Type	Width [min]	Area [mAU*s]	Height [mAU]	Area %
1	5.966	BB	0.1274	50.36583	5.90937	4.1989
2	8.063	BB	0.1561	1149.14246	115.49952	95.8011

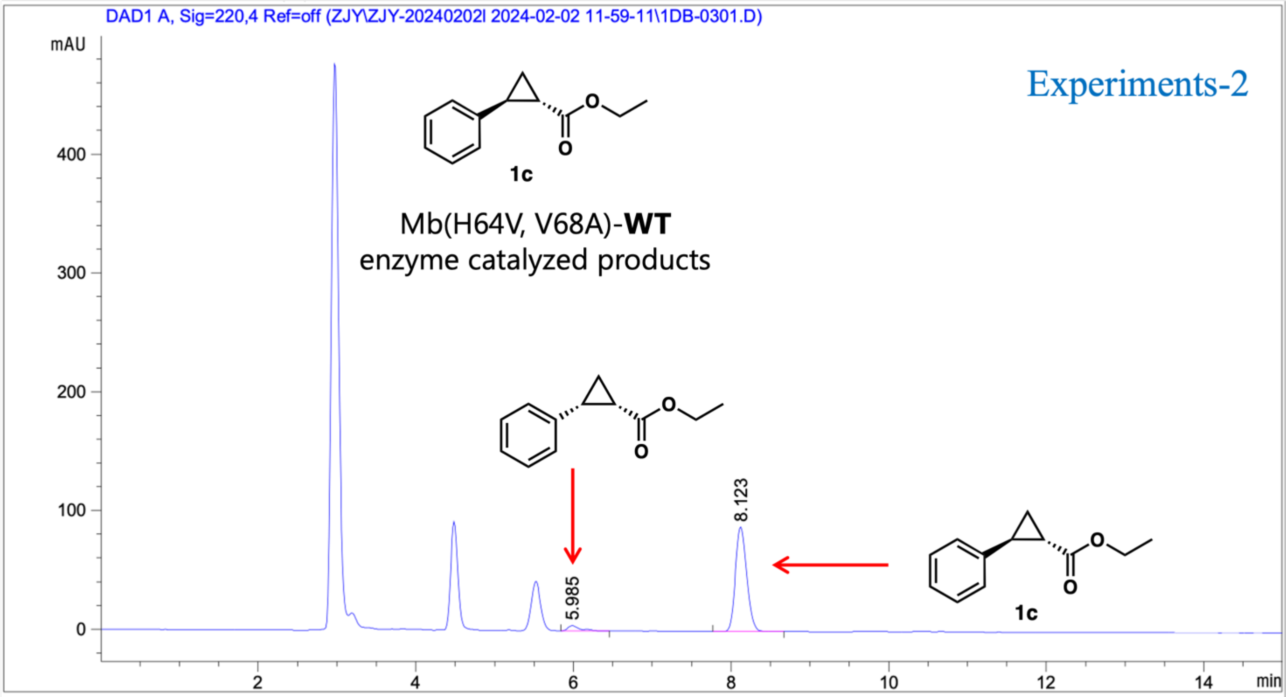

Peak #	RetTime [min]	Type	Width [min]	Area [mAU*s]	Height [mAU]	Area %
1	5.985	VB	0.1507	48.96679	4.65615	5.2980
2	8.123	BB	0.1559	875.27502	88.07709	94.7020

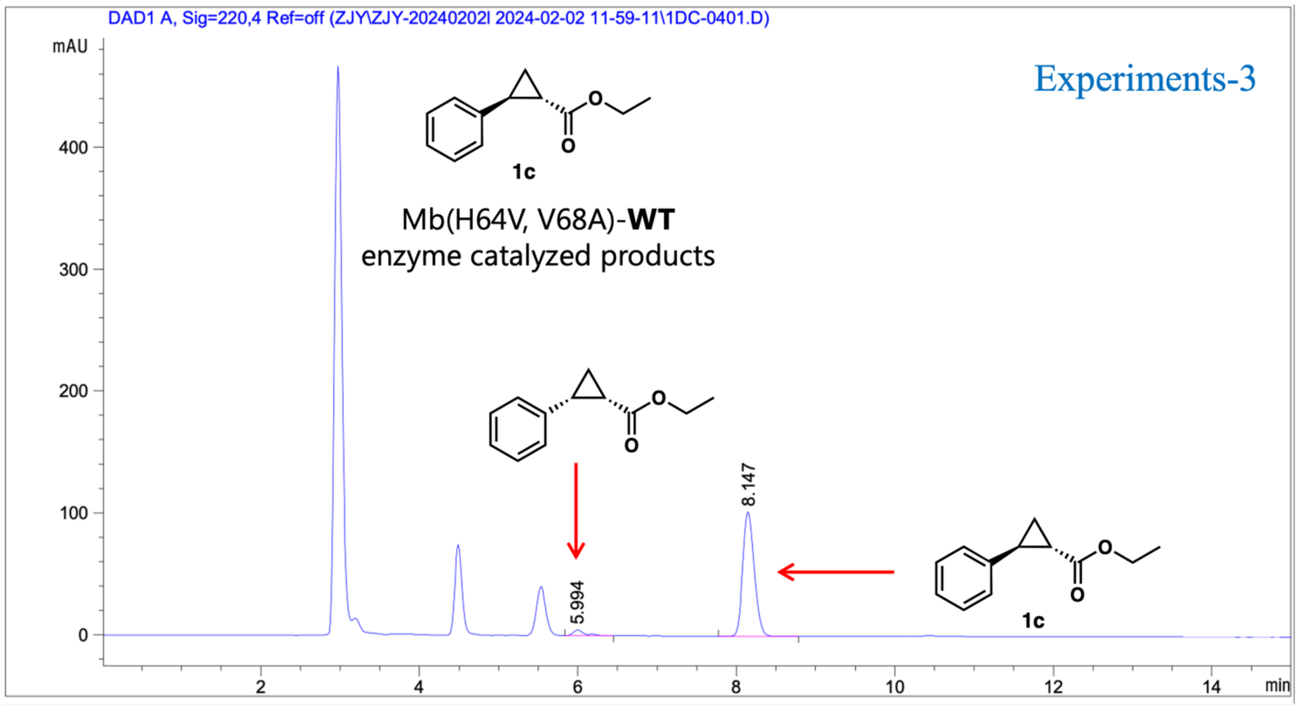

Peak #	RetTime [min]	Type	Width [min]	Area [mAU*s]	Height [mAU]	Area %
1	5.994	VB	0.1552	50.31539	4.76453	4.6843
2	8.147	BB	0.1569	1023.82318	102.17113	95.3157

Supplementary Figure 42: chiral HPLC spectrum for **1c**(Mb*-δMeH catalyzed product).

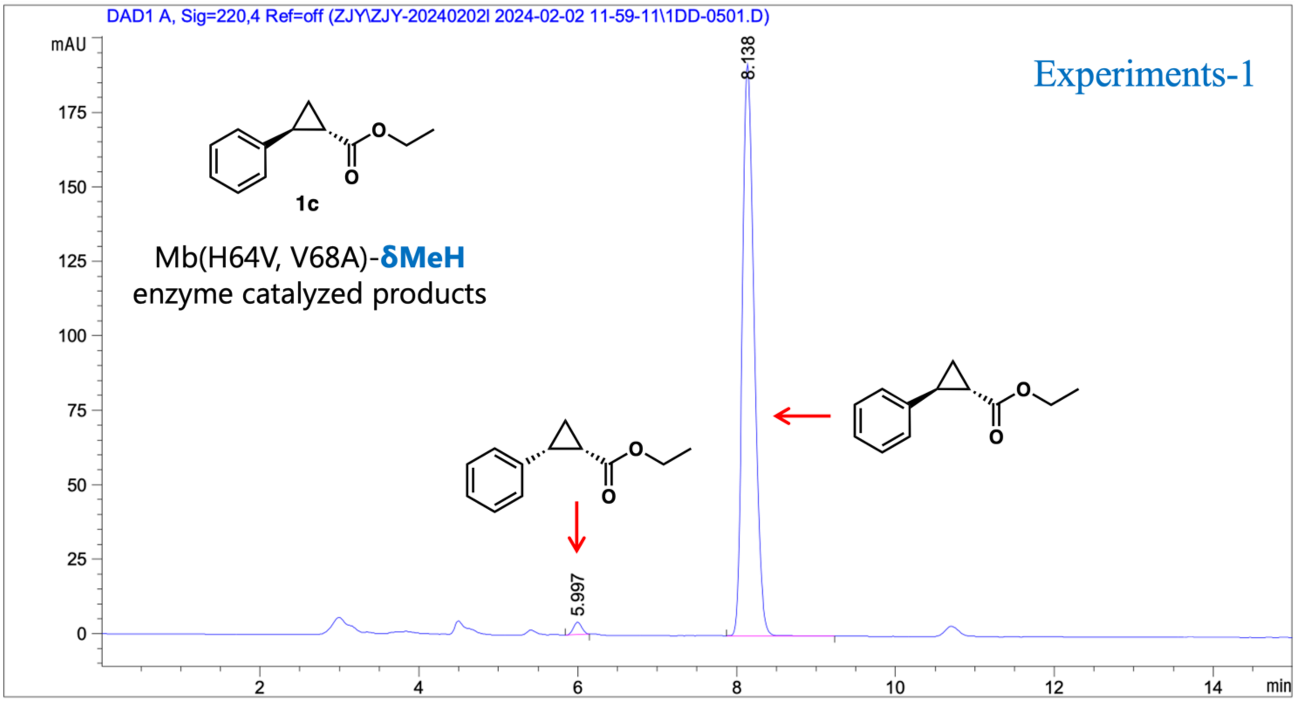

Peak #	RetTime [min]	Type	Width [min]	Area [mAU*s]	Height [mAU]	Area %
1	5.997	BB	0.1111	29.36757	4.12502	1.4611
2	8.138	BB	0.1603	1980.56921	192.03246	98.5389

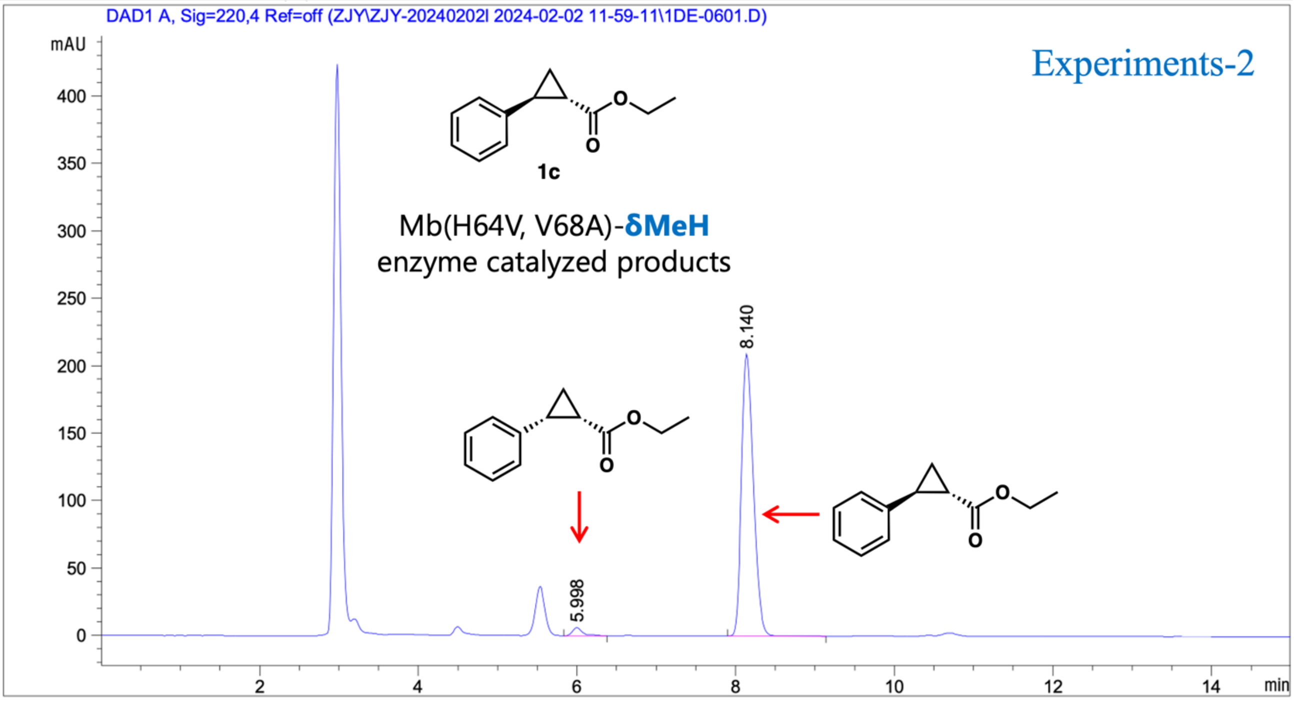

Peak #	RetTime [min]	Type	Width [min]	Area [mAU*s]	Height [mAU]	Area %
1	5.998	BB	0.1326	50.54970	5.86471	2.2783
2	8.140	BB	0.1610	2168.17017	209.04980	97.7217

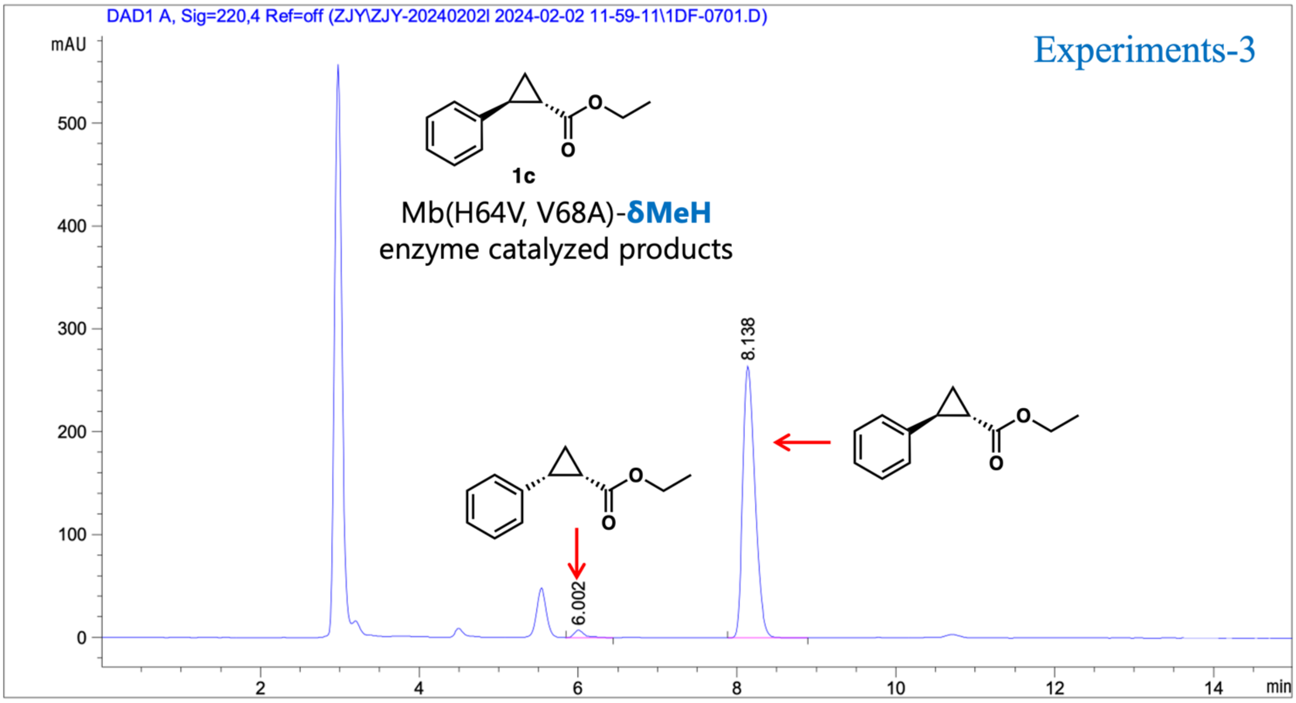

Peak #	RetTime [min]	Type	Width [min]	Area [mAU*s]	Height [mAU]	Area %
1	6.002	VB	0.1320	64.75498	7.26480	2.2579
2	8.138	BB	0.1639	2803.16626	264.05133	97.7421

Supplementary Figure 43: chiral HPLC spectrum for **1c**(Mb*-δMeH catalyzed product).

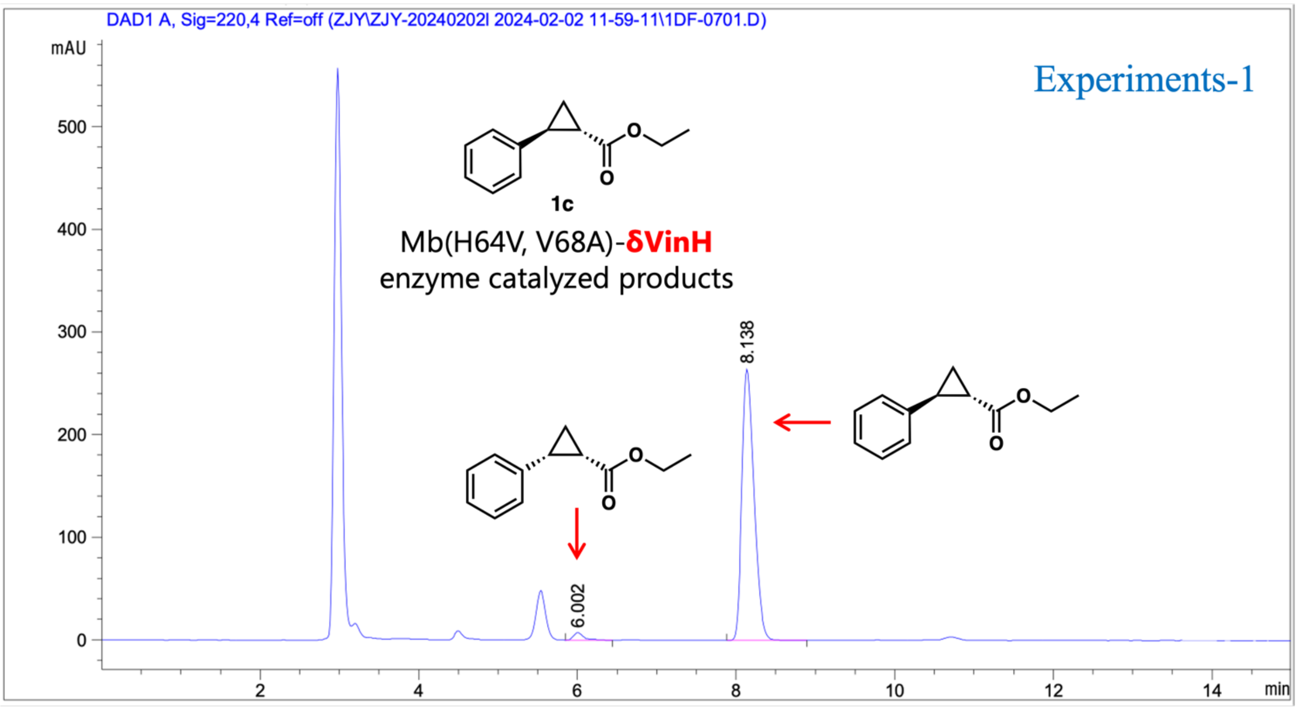

Peak #	RetTime [min]	Type	Width [min]	Area [mAU*s]	Height [mAU]	Area %
1	6.002	VB	0.1320	64.75498	7.26480	2.2579
2	8.138	BB	0.1639	2803.16626	264.05133	97.7421

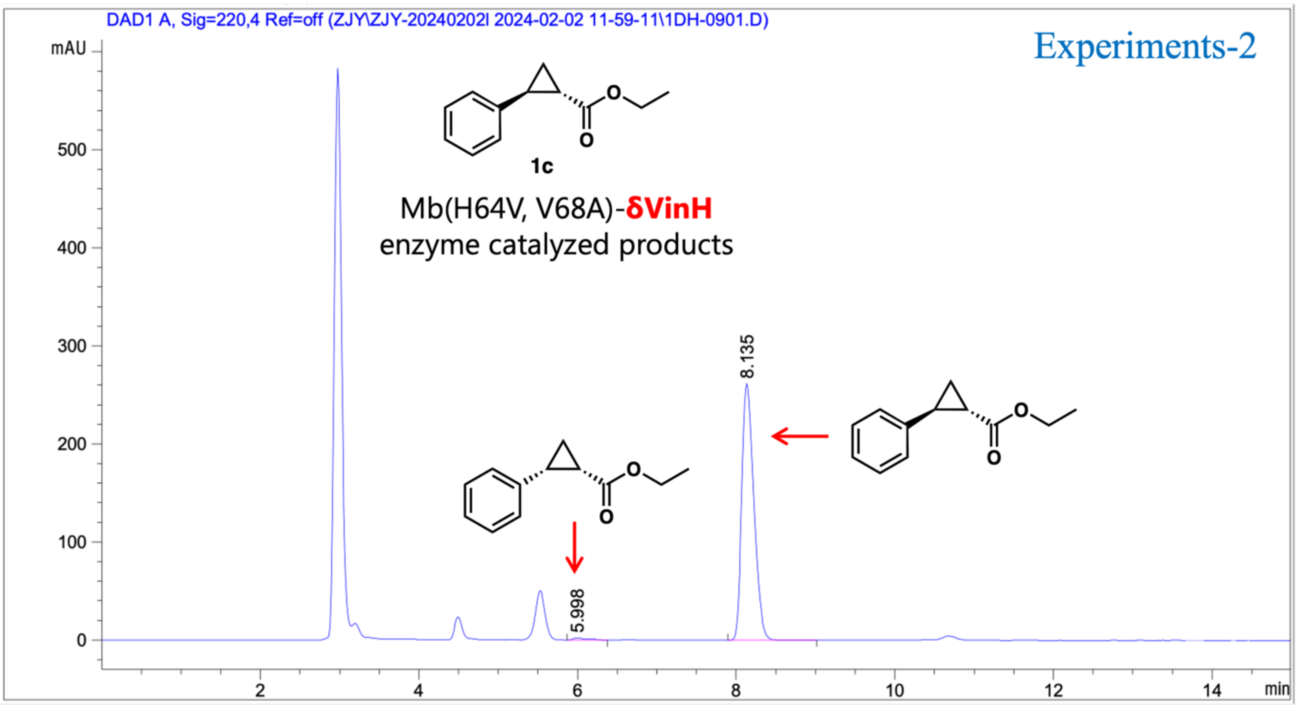

Peak #	RetTime [min]	Type	Width [min]	Area [mAU*s]	Height [mAU]	Area %
1	5.998	VB	0.1704	27.72541	2.33766	0.9914
2	8.135	BB	0.1634	2768.74170	261.71909	99.0086

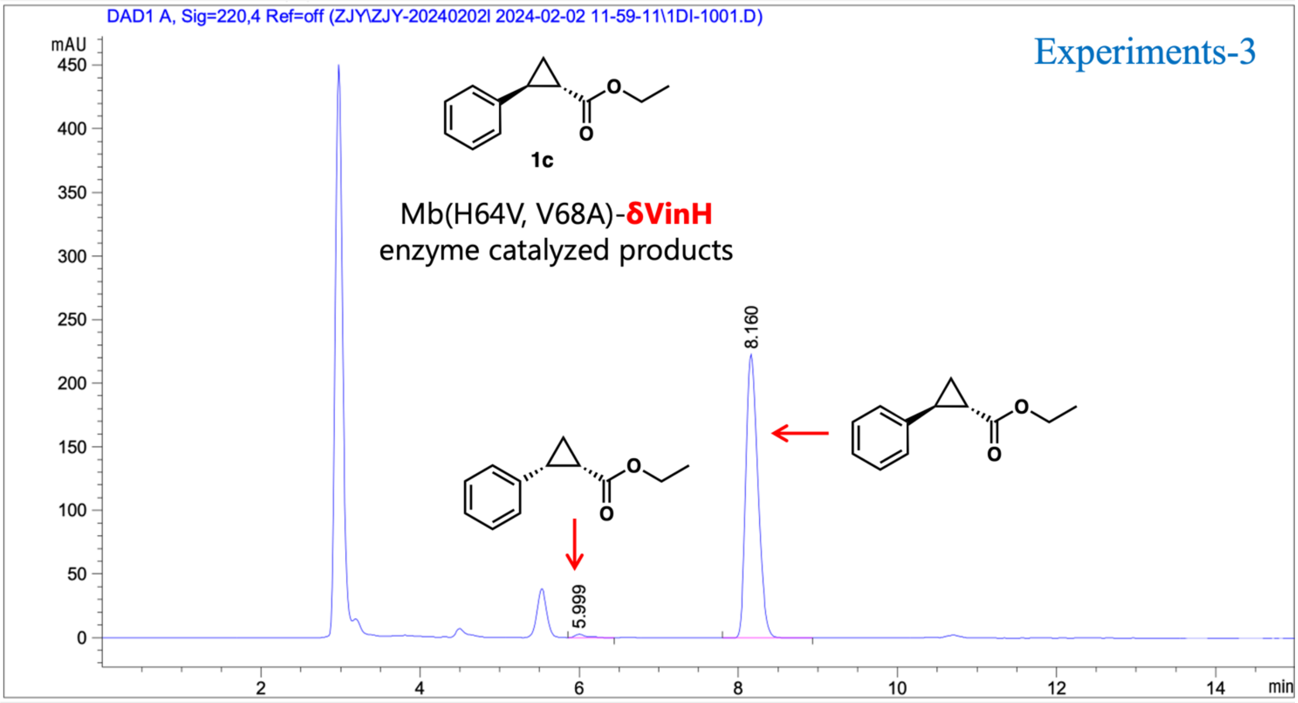

Peak #	RetTime [min]	Type	Width [min]	Area [mAU*s]	Height [mAU]	Area %
1	5.999	VB	0.1490	26.64035	2.65578	1.1272
2	8.160	BB	0.1663	2336.77588	222.84537	98.8728

Supplementary Figure 44: chiral HPLC spectrum for **1c**(Mb*-δVinH catalyzed product).

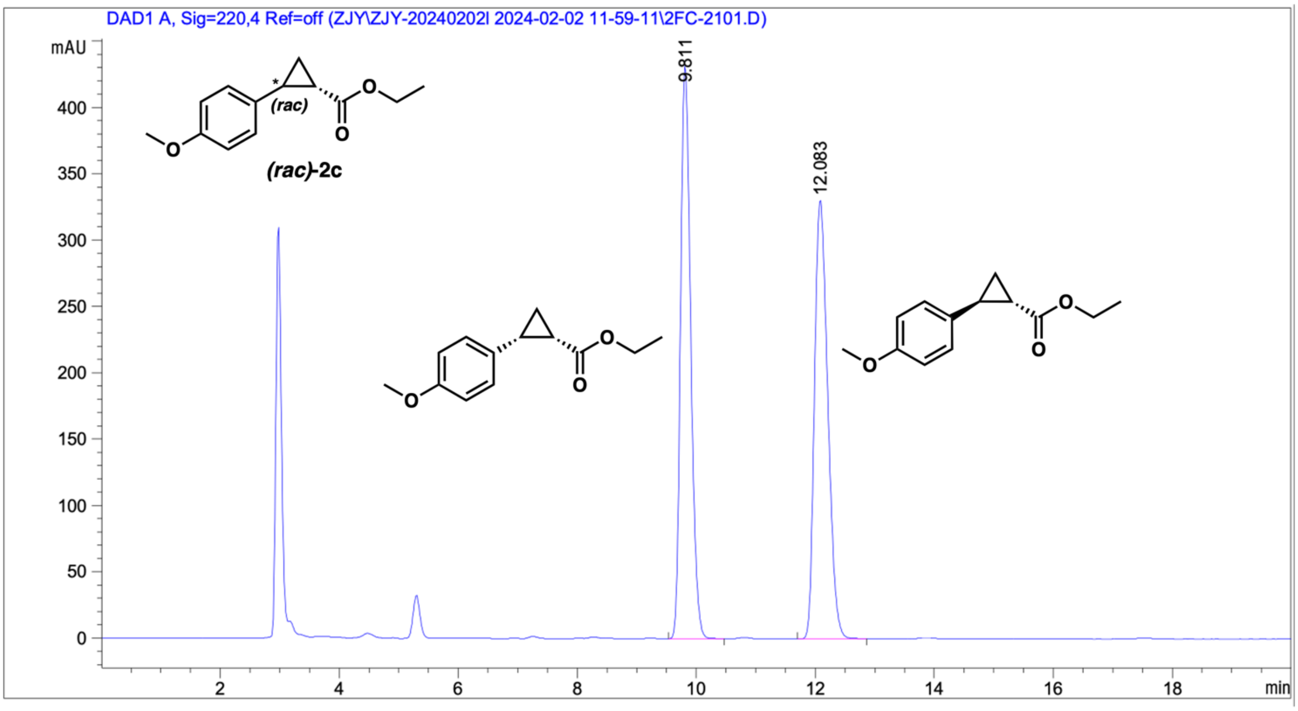

Peak #	RetTime [min]	Type	Width [min]	Area [mAU*s]	Height [mAU]	Area %
1	9.811	BB	0.1821	5104.73193	431.11008	49.9294
2	12.083	BB	0.2447	5119.17578	330.47058	50.0706

Supplementary Figure 45: chiral HPLC spectrum for ***(rac)*-2c**.

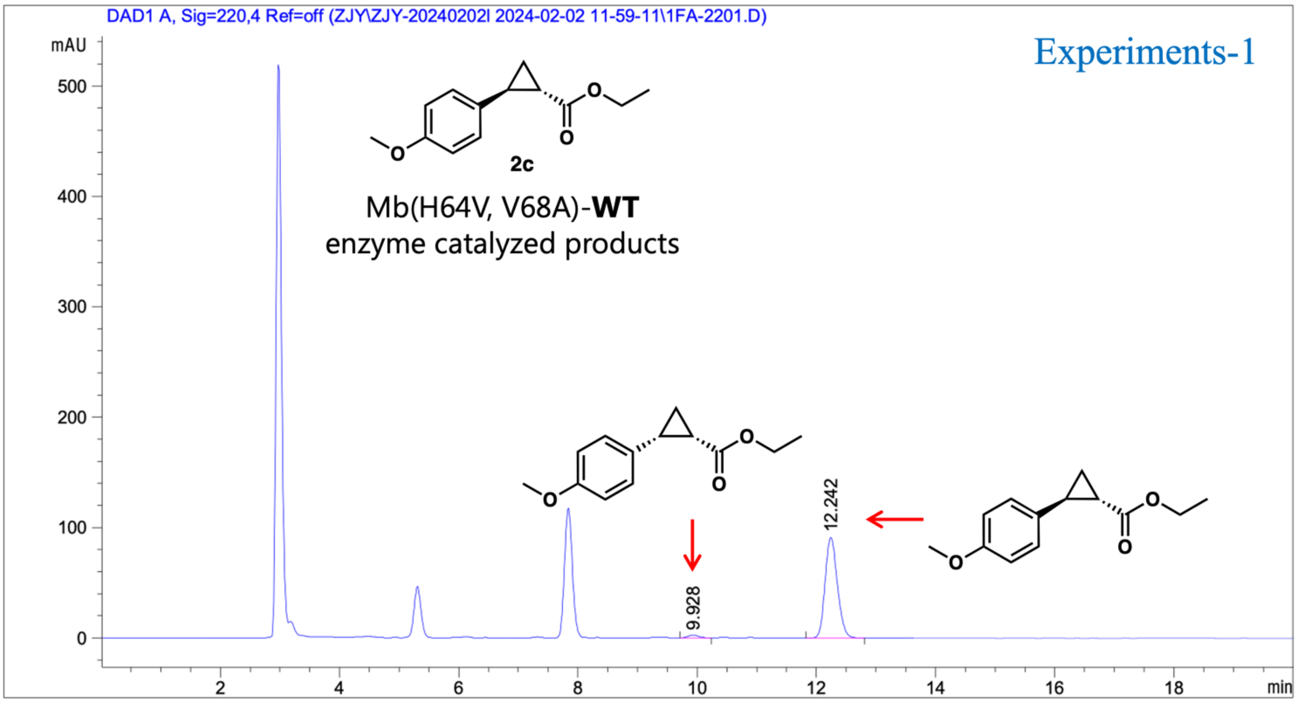

Peak #	RetTime [min]	Type	Width [min]	Area [mAU*s]	Height [mAU]	Area %
1	9.928	BB	0.1811	30.86967	2.62635	2.3174
2	12.242	BB	0.2225	1301.21118	91.18253	97.6826

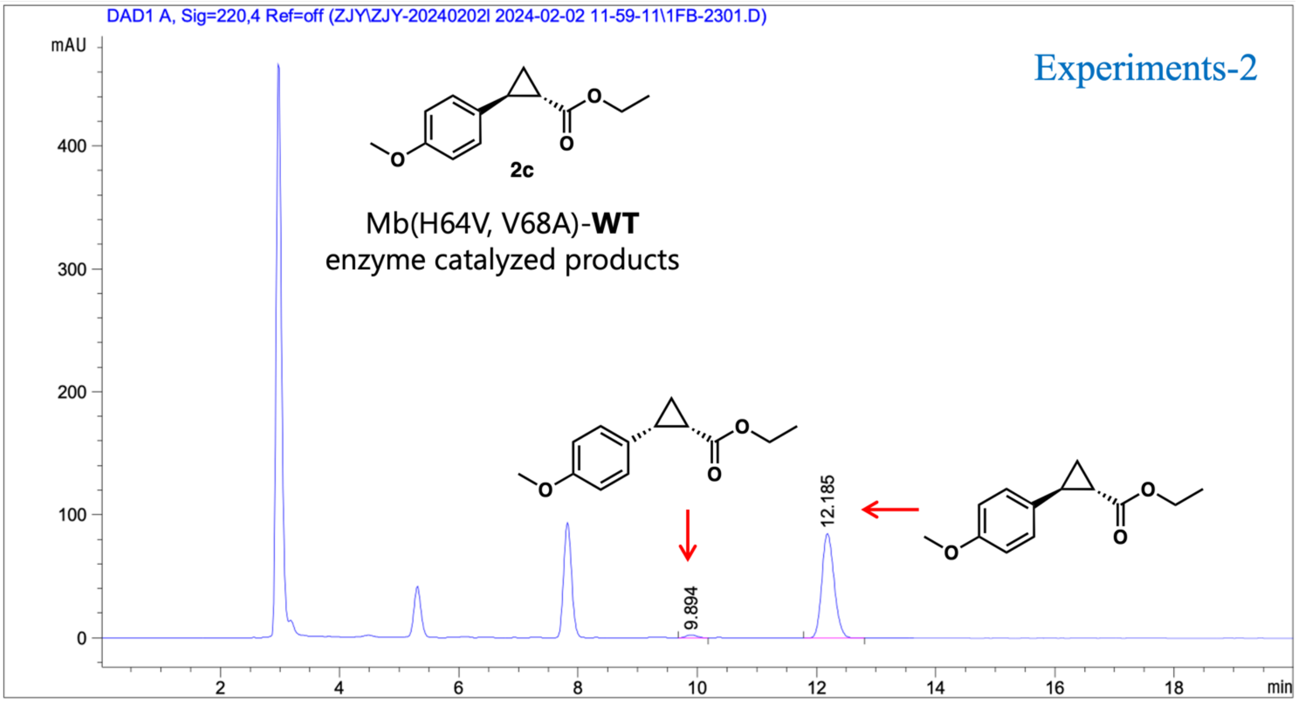

Peak #	RetTime [min]	Type	Width [min]	Area [mAU*s]	Height [mAU]	Area %
1	9.894	BB	0.1912	29.45900	2.39959	2.3616
2	12.185	BB	0.2231	1217.98157	85.01524	97.6384

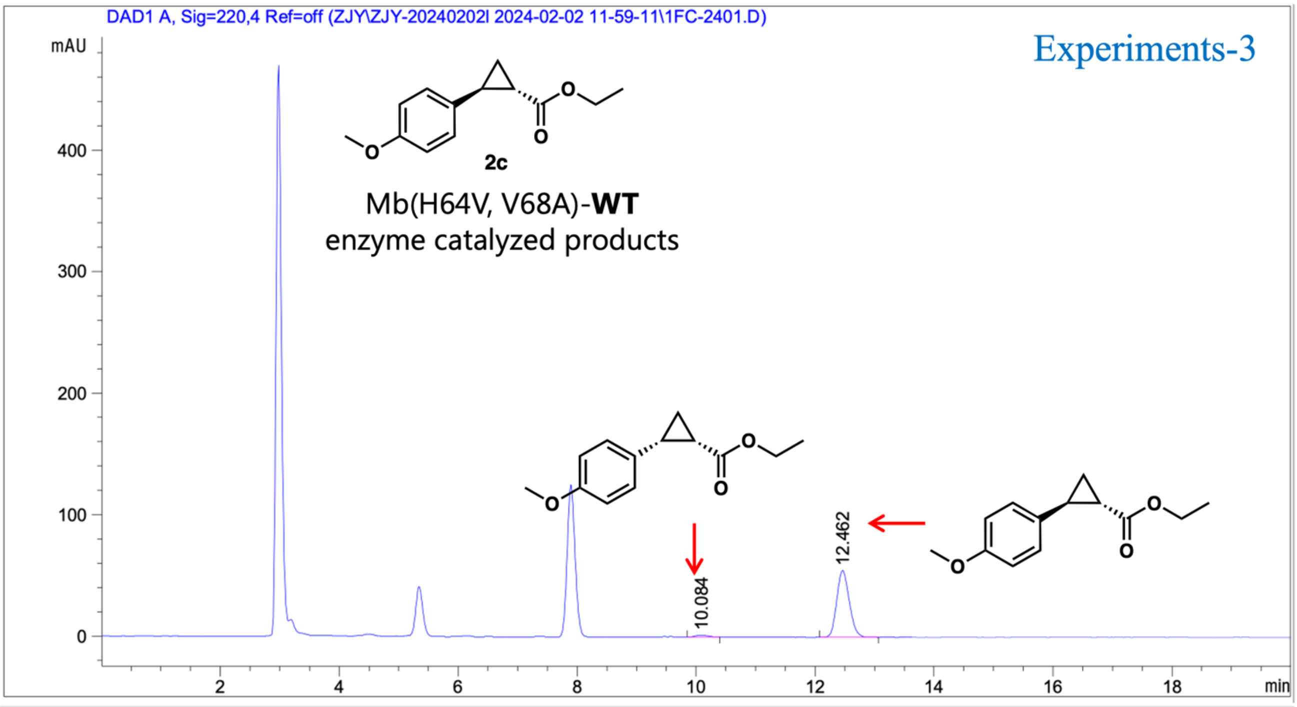

Peak #	RetTime [min]	Type	Width [min]	Area [mAU*s]	Height [mAU]	Area %
1	10.084	BB	0.2231	22.46895	1.60682	2.7336
2	12.462	BB	0.2257	799.48639	54.94935	97.2664

Supplementary Figure 46: chiral HPLC spectrum for **2c**(Mb*-WT catalyzed product).

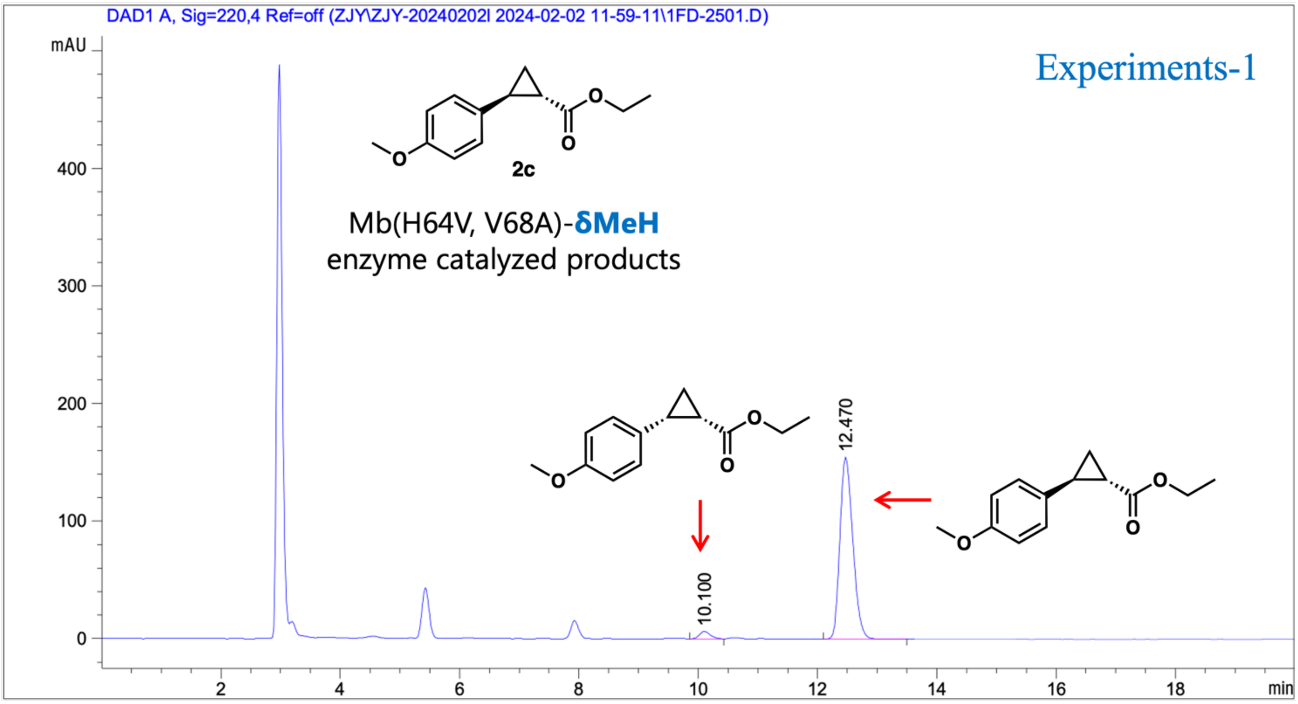

Peak #	RetTime [min]	Type	Width [min]	Area [mAU*s]	Height [mAU]	Area %
1	10.100	BV	0.1944	78.81776	6.28301	3.2896
2	12.470	BB	0.2310	2317.17480	154.44563	96.7104

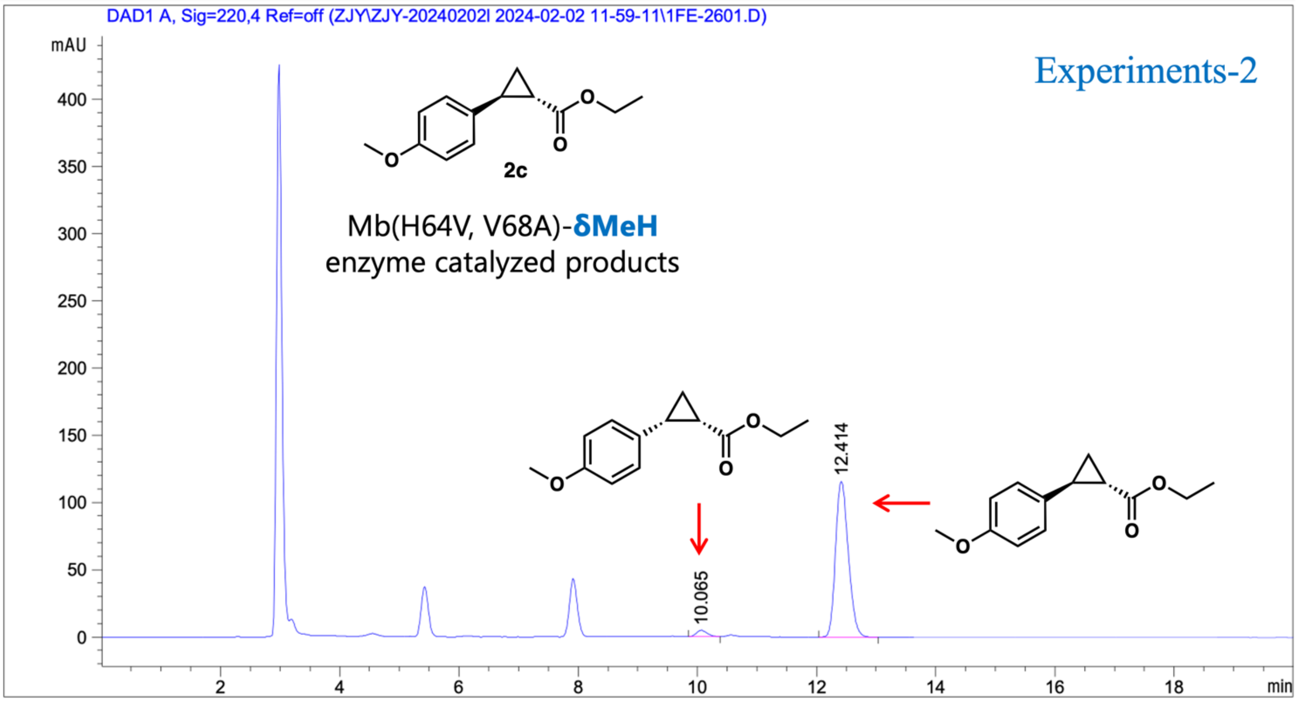

Peak #	RetTime [min]	Type	Width [min]	Area [mAU*s]	Height [mAU]	Area %
1	10.065	BV	0.1893	58.61205	4.70679	3.2835
2	12.414	BB	0.2297	1726.40991	115.98012	96.7165

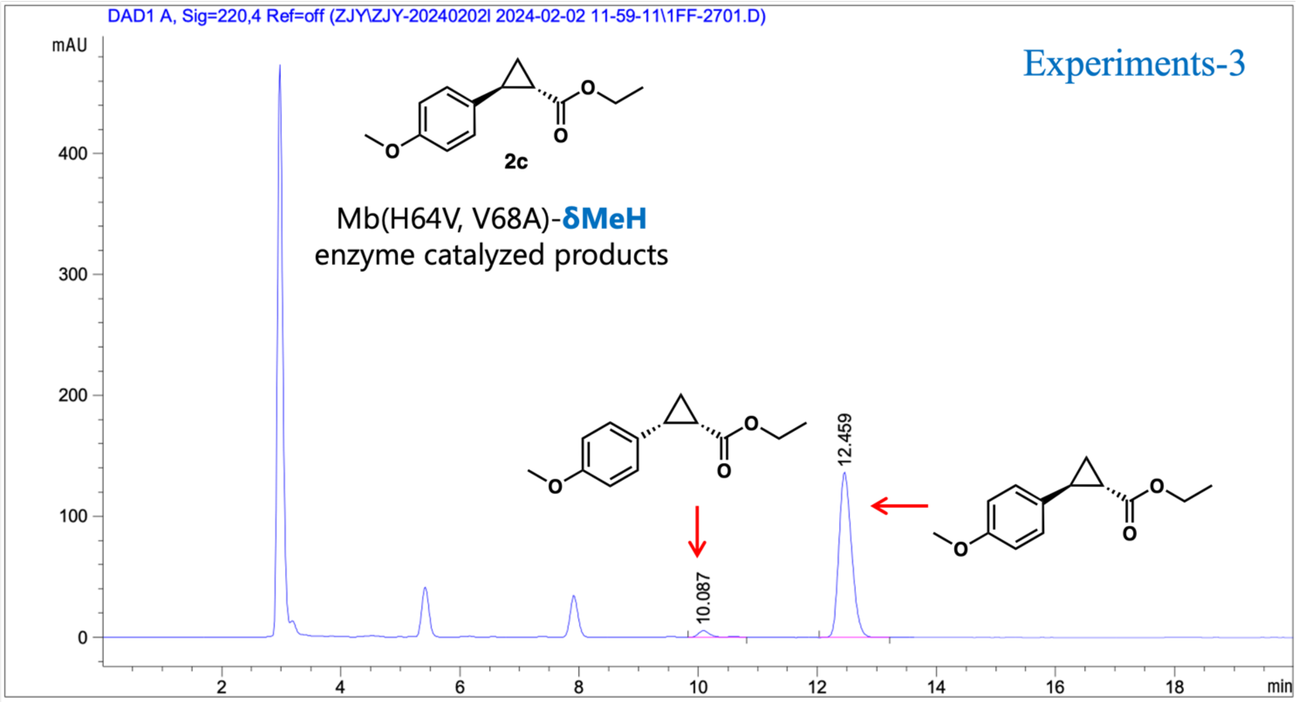

Peak #	RetTime [min]	Type	Width [min]	Area [mAU*s]	Height [mAU]	Area %
1	10.087	BV R	0.2099	78.21690	5.49987	3.7397
2	12.459	BB	0.2281	2013.31604	136.51485	96.2603

Supplementary Figure 47: chiral HPLC spectrum for **1c**(Mb*-δMeH catalyzed product).

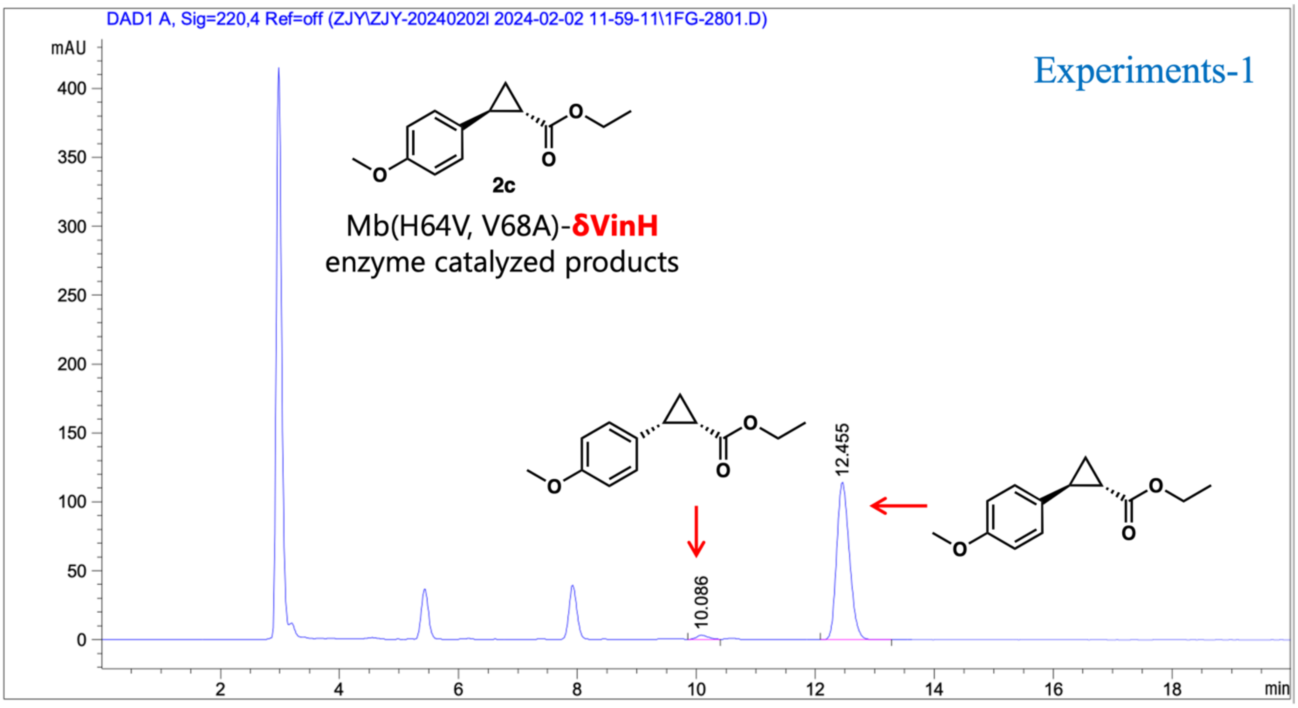

Peak #	RetTime [min]	Type	Width [min]	Area [mAU*s]	Height [mAU]	Area %
1	10.086	BB	0.2046	38.07755	2.91062	2.1971
2	12.455	BB	0.2330	1695.02661	114.30685	97.8029

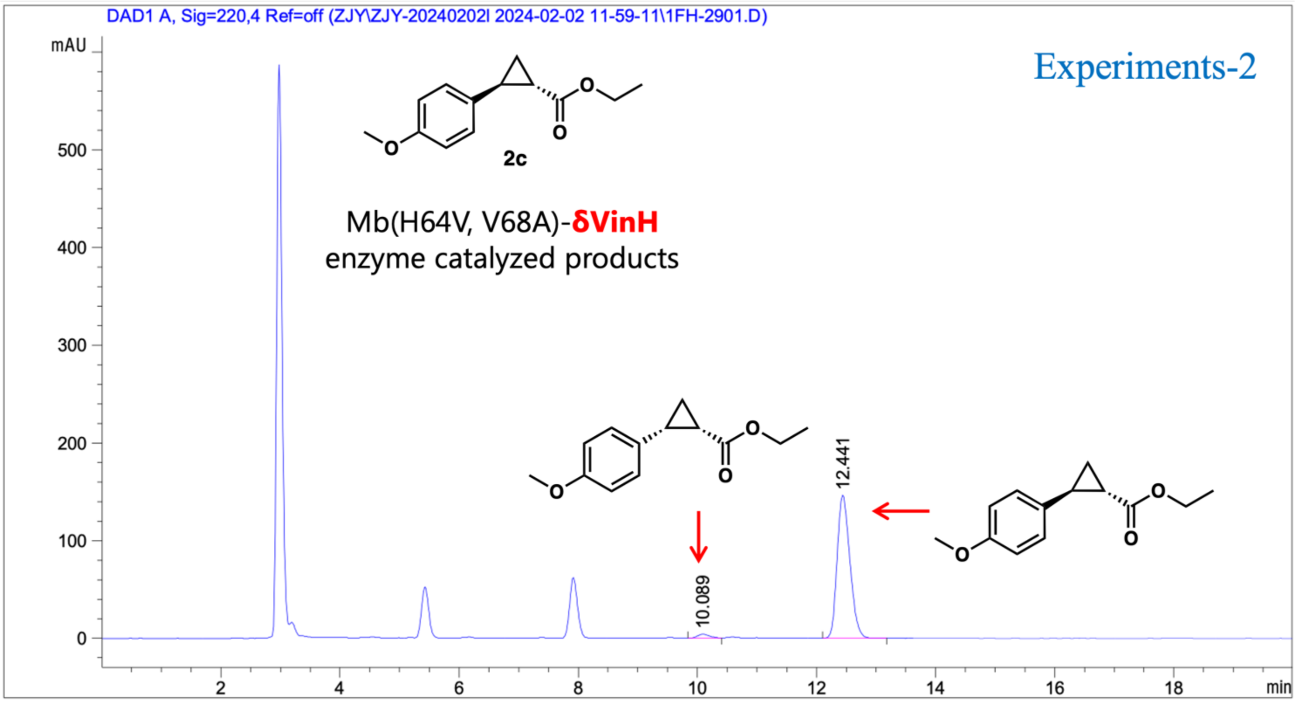

Peak #	RetTime [min]	Type	Width [min]	Area [mAU*s]	Height [mAU]	Area %
1	10.089	BV	0.2020	52.57850	3.88321	2.3605
2	12.441	BB	0.2296	2174.85425	146.17529	97.6395

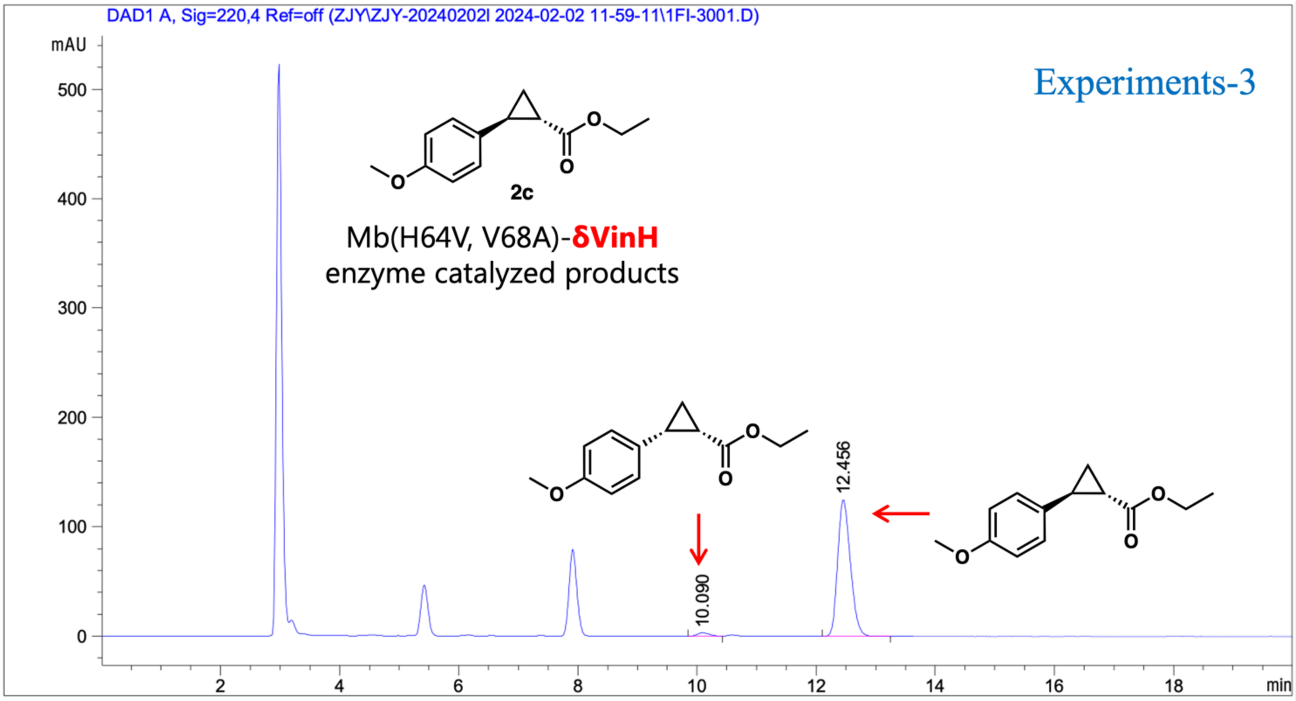

Peak #	RetTime [min]	Type	Width [min]	Area [mAU*s]	Height [mAU]	Area %
1	10.090	BV	0.2110	43.64253	3.12372	2.3018
2	12.456	BB	0.2291	1852.40991	124.85703	97.6982

Supplementary Figure 48: chiral HPLC spectrum for **2c**(Mb*-δVinH catalyzed product).

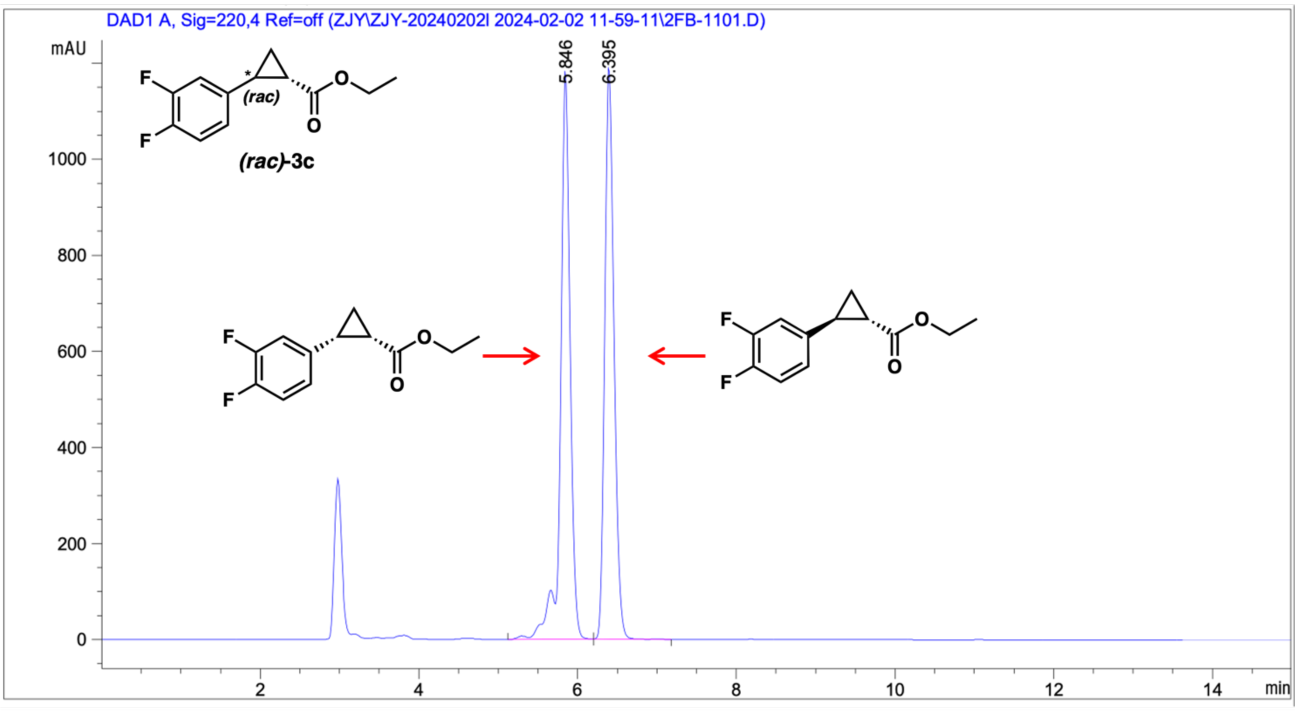

Peak #	RetTime [min]	Type	Width [min]	Area [mAU*s]	Height [mAU]	Area %
1	5.846	VB R	0.1199	1.02089e4	1184.69202	50.7906
2	6.395	BV R	0.1286	9891.04883	1193.99414	49.2094

Supplementary Figure 49: chiral HPLC spectrum for ***(rac)*-3c**.

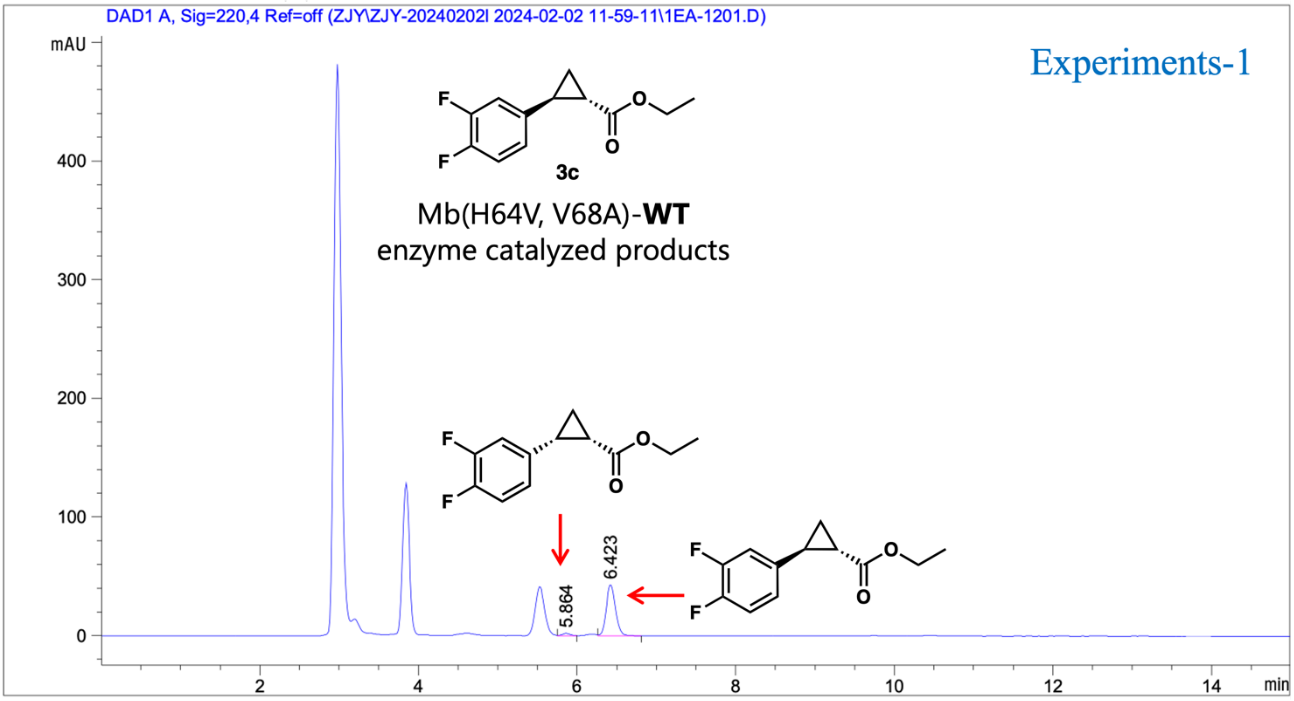

Peak #	RetTime [min]	Type	Width [min]	Area [mAU*s]	Height [mAU]	Area %
1	5.864	FM R	0.1293	15.73533	2.02899	4.1965
2	6.423	FM R	0.1375	359.23148	43.55051	95.8035

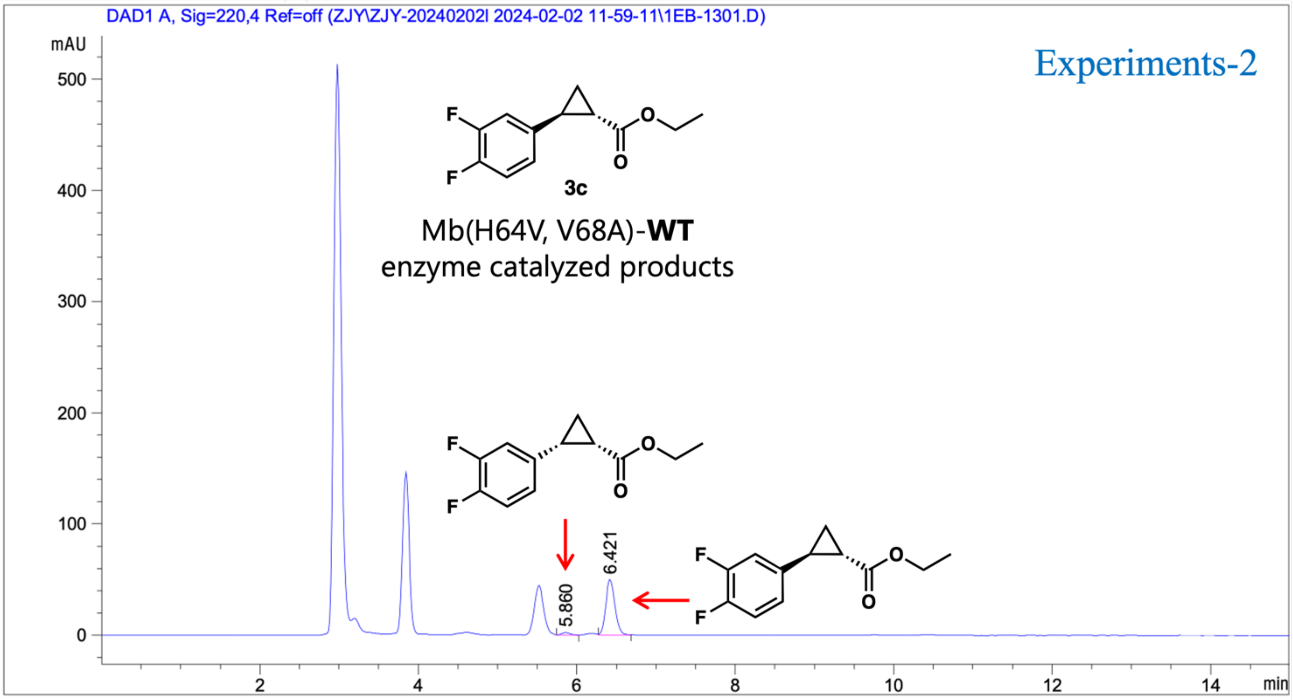

Peak #	RetTime [min]	Type	Width [min]	Area [mAU*s]	Height [mAU]	Area %
1	5.860	FM R	0.1359	18.68946	2.29235	4.4046
2	6.421	FM R	0.1346	405.62570	50.23064	95.5954

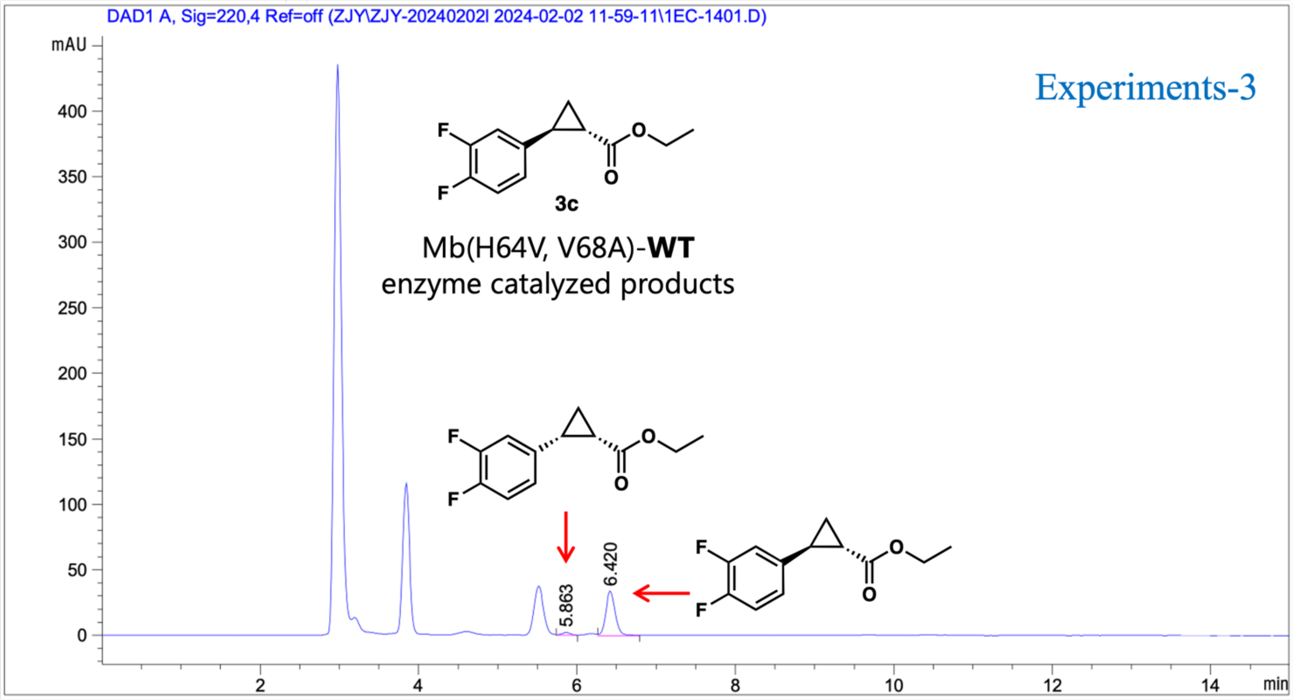

Peak #	RetTime [min]	Type	Width [min]	Area [mAU*s]	Height [mAU]	Area %
1	5.863	FM R	0.1307	15.77081	2.01102	5.1980
2	6.420	FM R	0.1394	287.63278	34.39794	94.8020

Supplementary Figure 50: chiral HPLC spectrum for **3c**(Mb*-WT catalyzed product).

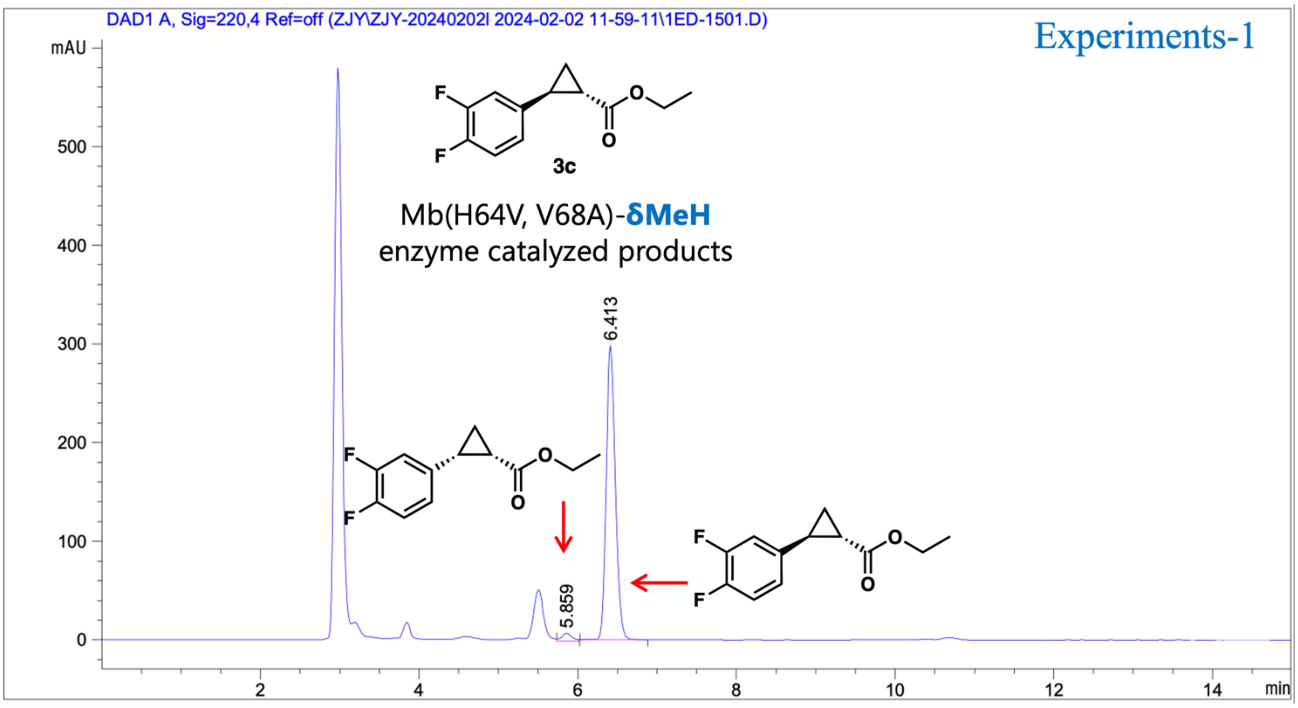

Peak #	RetTime [min]	Type	Width [min]	Area [mAU*s]	Height [mAU]	Area %
1	5.859	FM R	0.1489	70.69524	7.91060	2.8467
2	6.413	BB	0.1265	2412.72388	297.82269	97.1533

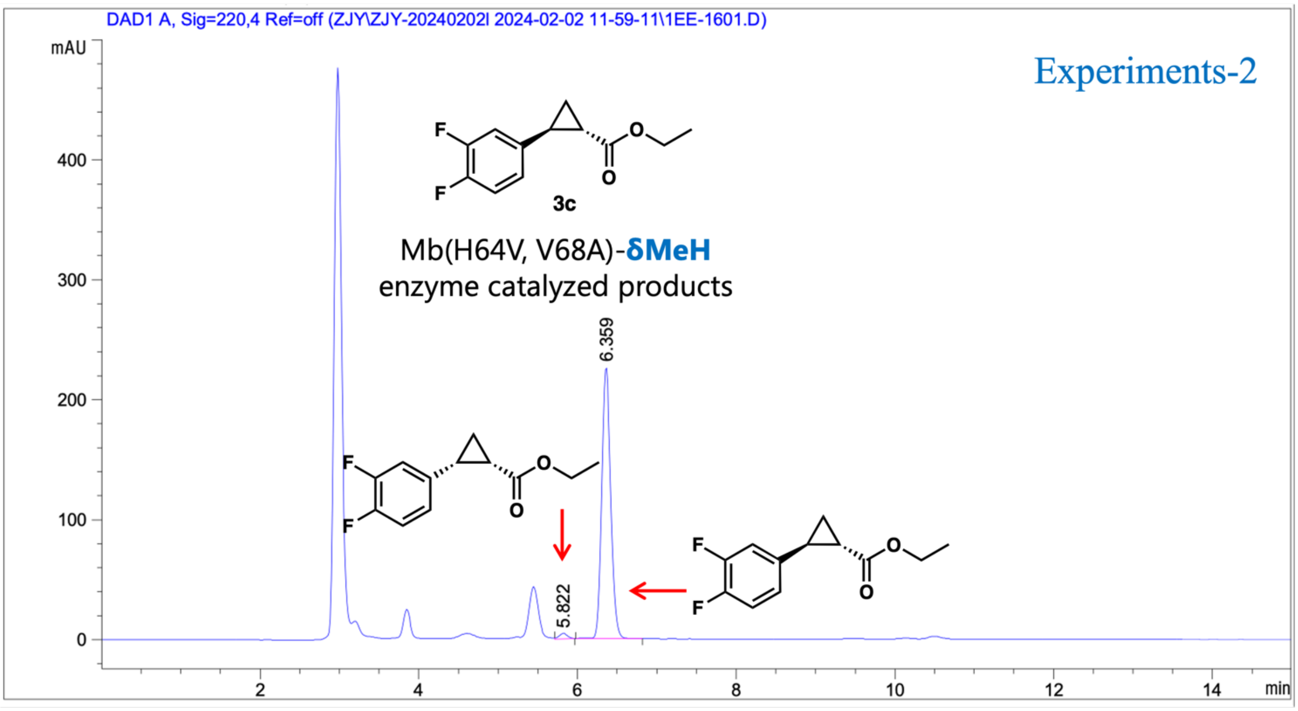

Peak #	RetTime [min]	Type	Width [min]	Area [mAU*s]	Height [mAU]	Area %
1	5.822	MM R	0.1231	34.25836	4.63669	1.8515
2	6.359	VB	0.1253	1816.07996	226.97520	98.1485

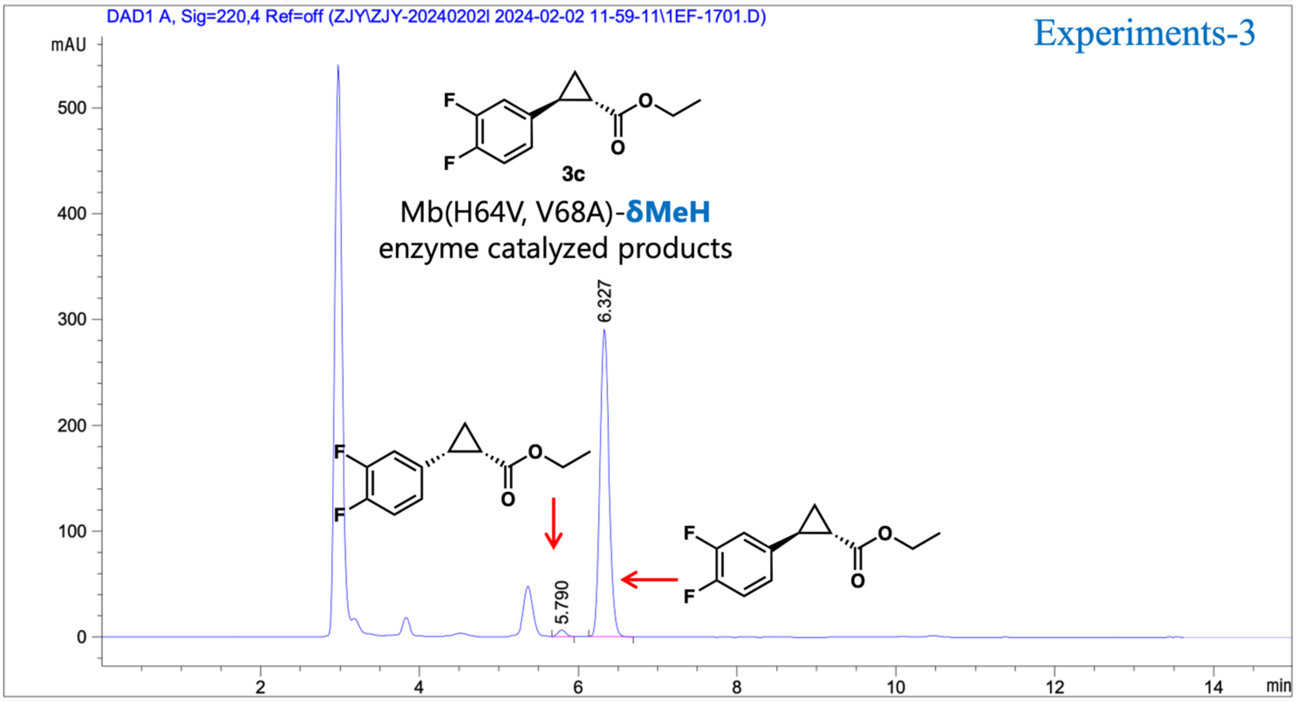

Peak #	RetTime [min]	Type	Width [min]	Area [mAU*s]	Height [mAU]	Area %
1	5.790	VB	0.1106	44.77601	6.32438	1.8917
2	6.327	BB	0.1250	2322.15186	291.36935	98.1083

Supplementary Figure 51: chiral HPLC spectrum for **3c**(Mb*-δMeH catalyzed product).

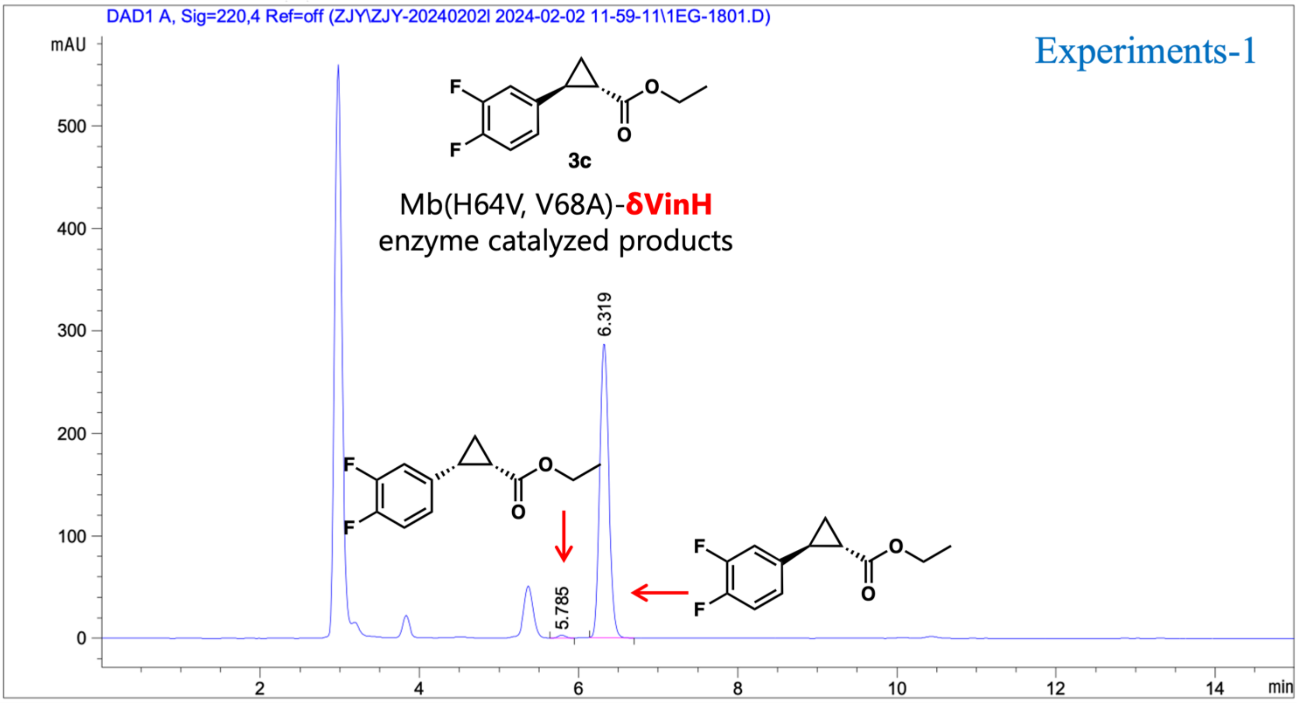

Peak #	RetTime [min]	Type	Width [min]	Area [mAU*s]	Height [mAU]	Area %
1	5.785	MM R	0.1272	22.56845	2.95628	0.9730
2	6.319	MM R	0.1324	2296.98755	289.08282	99.0270

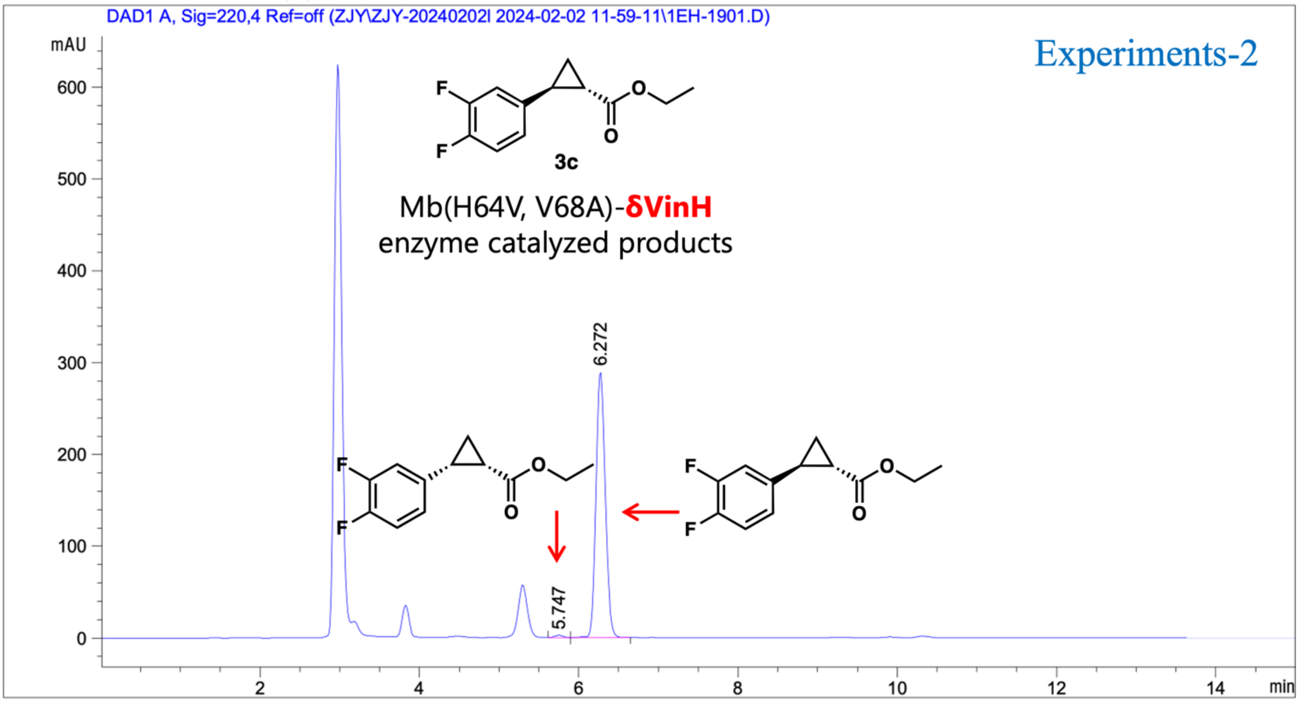

Peak #	RetTime [min]	Type	Width [min]	Area [mAU*s]	Height [mAU]	Area %
1	5.747	VV	0.1123	18.35921	2.53950	0.7894
2	6.272	VB	0.1248	2307.26440	289.97260	99.2106

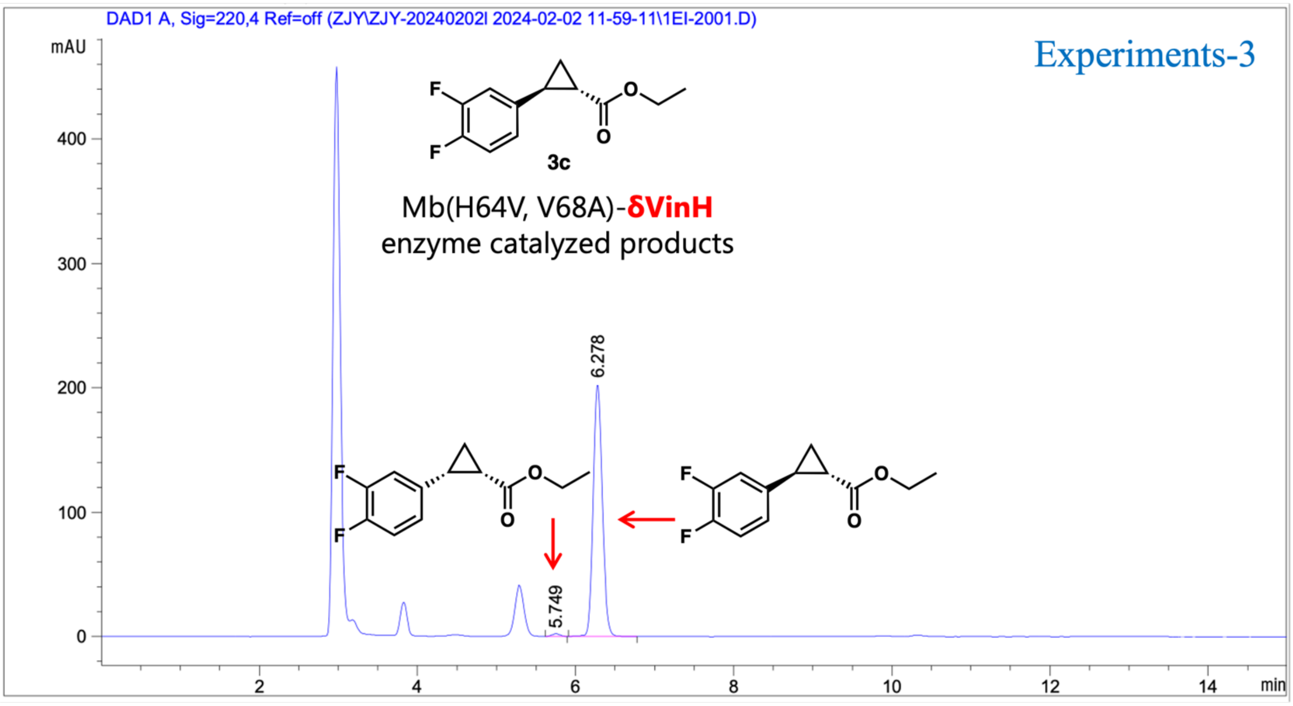

Peak #	RetTime [min]	Type	Width [min]	Area [mAU*s]	Height [mAU]	Area %
1	5.749	VB	0.1095	14.08630	2.01677	0.8676
2	6.278	BB	0.1244	1609.44141	203.26932	99.1324

Supplementary Figure 52: chiral HPLC spectrum for **3c**(Mb*-δVinH catalyzed product).

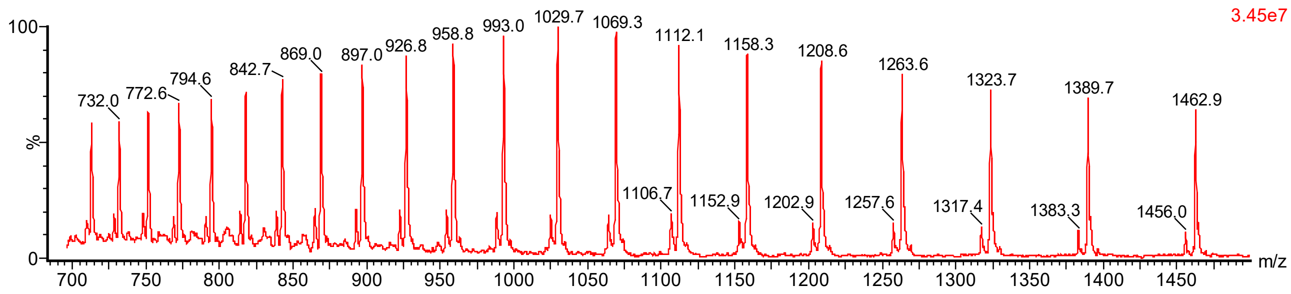

Supplementary Figure 53: MS result before deconvolution: GFP-D190-δVinH.

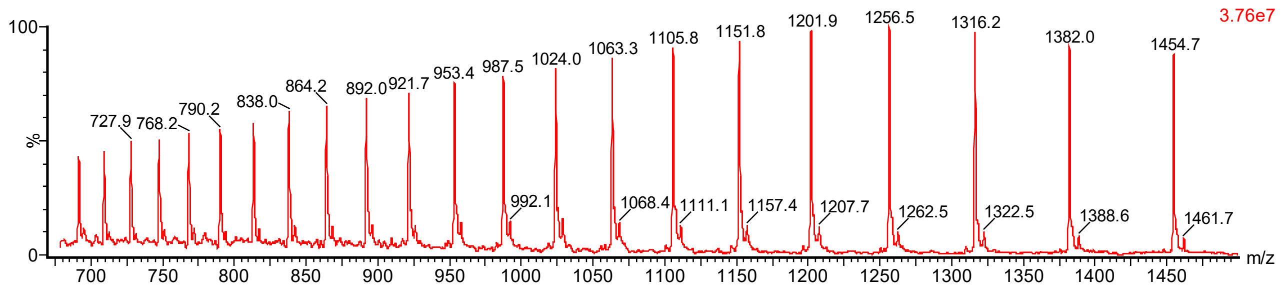

Supplementary Figure 54: MS result before deconvolution: OE1.3-H23-δVinH.

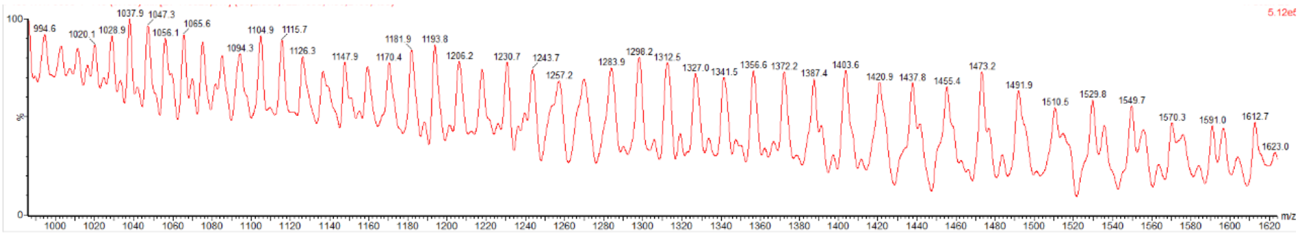

Supplementary Figure 55: MS result before deconvolution: P450-H400-δVinH.

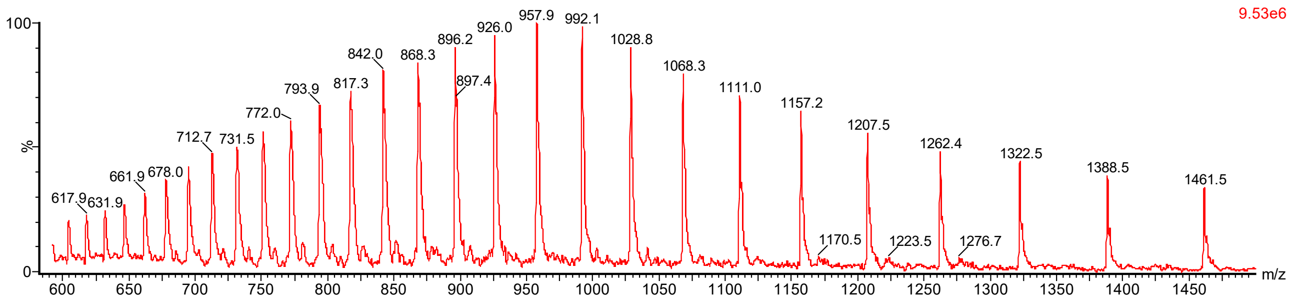

Supplementary Figure 56: MS result before deconvolution: APEX-H163-δVinH.

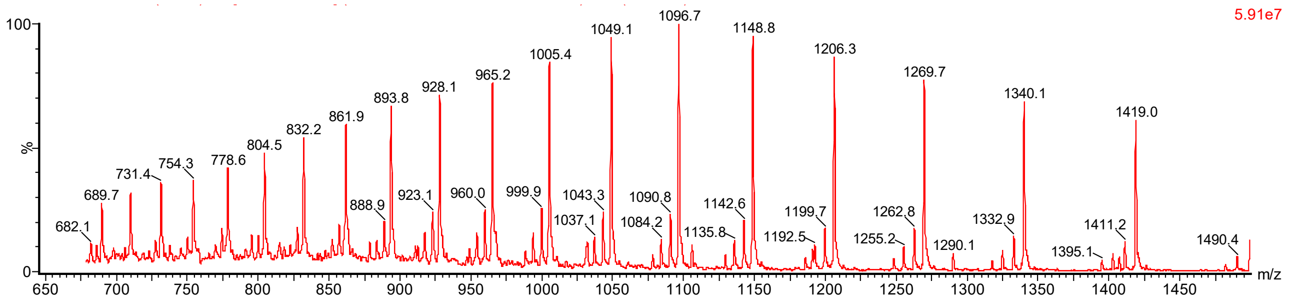

Supplementary Figure 57 MS result before deconvolution: dnHEM1.2-δVinH.

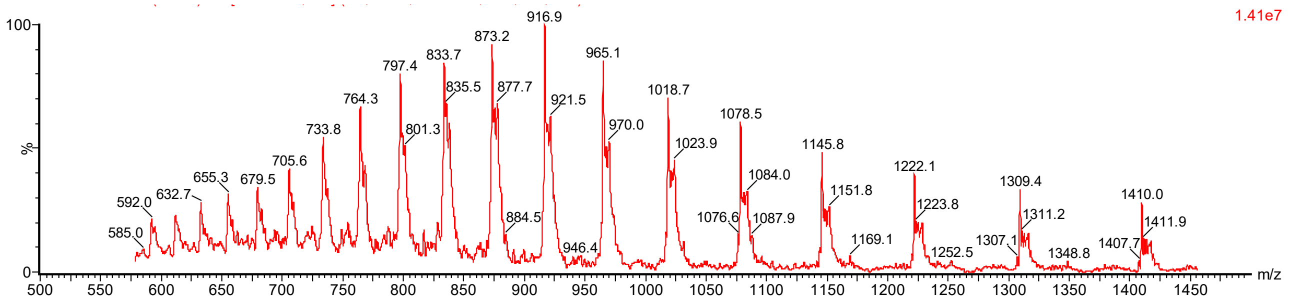

Supplementary Figure 58: MS result before deconvolution: Mb-H93-δVinH.

Supplementary Tables (1-6)

Supplementary Table 1: Summary of the extinction coefficients of the different myoglobin variants.

Variants	Wavelength Soret band in nm	ε(Soret) in mM^{-1} cm^{-1}
Mb*-WT	412	135.12±1.30
Mb*-H93-δMe-H	412	134.93±6.57
Mb*-H93-δVin-H	412	130.22±2.56

Supplementary Table 2: Heme loading efficiencies of Mb* variants.

Name	Heme loading in %
Mb*-WT	46.1±2.2
Mb*-H93-δVin-H	40.7±2.6
Mb*-H93-δMe-H	35.7±3.1

Supplementary Table 3: Summary of enantiomeric excess (ee) percent for Mb variants catalyzed cyclopropanation of styrene

	Mb variants	ee %
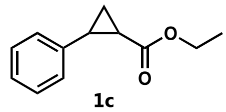 1c	Mb(H64V, V68A)-WT	90±1.1% ee
	Mb(H64V, V68A)-H93-δVin-H	96±0.9% ee
	Mb(H64V, V68A)-H93-δMe-H	97±1.4% ee
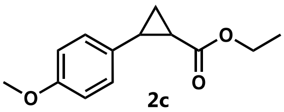 2c	Mb(H64V, V68A)-WT	95±0.5% ee
	Mb(H64V, V68A)-H93-δVin-H	93±0.5% ee
	Mb(H64V, V68A)-H93-δMe-H	95±0.2% ee
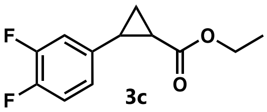 3c	Mb(H64V, V68A)-WT	91±1.1% ee
	Mb(H64V, V68A)-H93-δVin-H	95±1.1% ee
	Mb(H64V, V68A)-H93-δMe-H	98±0.2% ee

Supplementary Table 4: The cycloaddition reaction catalyzed by Mb(H64V, V68A)-H93-δVinH.

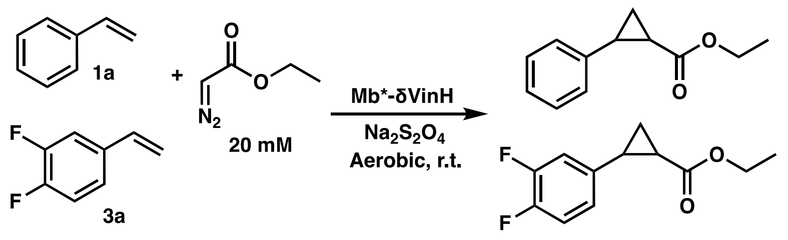

Entry	Substrate (mM)	$Na_2S_2O_4$ (mM)	Cat. (mol%)	Time (min)	Conversion (%)
1	1a	10	0.1	5	77.1
2	1a	20	0.1	5	76.1
3	1a	50	0.1	5	72.0
4	1a	100	0.1	5	78.4
5	1a	10	0.05	5	55.2
6	1a	10	0.02	5	26.6
7	3a	10	0.1	60	79.5
8	3a	10	0.1	30	76.8
9	3a	10	0.1	5	78.5

Supplementary Table 5: Cartesian coordinates (Å) for the DFT-optimized structures:

Imidazole

C -0.64590000 0.06870000 0.00000000

C 0.21600000 1.13840000 0.00000000

C 1.47130000 -0.60140000 0.00000000

N 0.17930000 -1.03860000 -0.00010000

H -0.12220000 -2.00150000 0.00050000

H 2.30960000 -1.28060000 -0.00020000

N 1.53270000 0.71370000 0.00000000

C -2.13360000 -0.02310000 0.00000000

H -2.50510000 -0.55190000 0.88300000

H -2.56510000 0.97840000 -0.00020000

H -2.50490000 -0.55220000 -0.88290000

H -0.04210000 2.18660000 0.00010000

N-methylimidazole

C 0.28743300 -0.70240400 0.00014100

C -0.98837500 -1.21471500 -0.00008400

C -1.22384400 0.91402300 0.00005600

N 0.11968800 0.67167100 0.00025600

H -1.61918600 1.91857500 0.00017100

N -1.92591100 -0.20087200 -0.00011500

C 1.62579500 -1.35707100 -0.00000100

H 2.21381900 -1.08457900 0.88219700

H 1.50270700 -2.44053500 0.00013400

H 2.21343500 -1.08478700 -0.88254100

H -1.27531300 -2.25563200 -0.00008400

C 1.17840600 1.66984300 -0.00013700

H 1.80352000 1.56679800 0.88834600

H 1.80274100 1.56691300 -0.88918700

H 0.72534400 2.65960400 0.00013400

N-vinyl-imidazole

C 0.91528800 0.53788800 -0.00528600

C 1.82970000 -0.48030700 -0.03476100

C -0.08281500 -1.45058700 0.04248600

H -0.87740000 -2.17717100 0.09241700

N 1.20022900 -1.71393000 -0.00179000

C 1.08628400 2.01751000 -0.02440900

H 0.67704900 2.49003700 0.87379100

H 2.14960300 2.25393300 -0.07030600

H 0.60167600 2.47545800 -0.89175800

H 2.90448400 -0.39276500 -0.07536100

C -1.57615300 0.55643200 0.07459500

C -2.76045900 -0.02895400 -0.08088500

H -1.49577900 1.62122800 0.24789800

H -3.65838300 0.56967800 -0.01539700

H -2.87815400 -1.08805500 -0.27017400

N -0.32796700 -0.09524600 0.04156800

Supplementary Table 6: DNA sequence and protein sequence in this paper

The incorporation sites of UAA are highlighted in yellow.

**1**	**chPylRS-IPYE-δMe-H** “IPYE” mutations (V31I, T56P, H62Y, and A100E) and fixed mutation (Y349F) were labeled in blue.
**DNA sequence**	ATGGATAAGAAGCCGCTGGATGTTCTGATCTCTGCGACCGGTCTGTGGATGTCCCGTA<br>CCGGCACGCTGCACAAGATCAAGCACTATGAGATTTCTCGTTCTAAAATCTACATCGA<br>AATGGCGTGTGGTGACCATCTGGTTGTGAACAACTCTCGTTCTTGTCGTCCCGCACGT<br>GCATTCCGTTATCATAAATACCGTAAAACCTGCAAACGTTGTCGTGTTTCTGACGAAG<br>ATATCAACAACTTCCTGACCCGTTCTACCGAAGGCAAAACCTCTGTTAAAGTTAAAG<br>TTGTTTCTGAGCCGAAAGTGAAAAAAGCGATGCCGAAATCTGTTTCTCGTGCGCCGA<br>AACCGCTGGAAAATCCGGTTTCTGCGAAAGCGTCTACCGACACCTCTCGTTCTGTTC<br>CGTCTCCGGCGAAATCTACCCCGAACTCTCCGGTTCCGACCTCTGCAAGCGCCCCAG<br>CTCTGACTAAATCCCAGACGGACCGTCTGGAGGTGCTGCTGAACCCAAAGGATGAA<br>ATCTCTCTGAACAGCGGCAAGCCTTTCCGTGAGCTGGAAAGCGAGCTGCTGTCTCGT<br>CGTAAAAAGGATCTGCAACAGATCTACGCTGAGGAACGCGAGAACTATCTGGGTAAG<br>CTGGAGCGCGAAATTACTCGCTTCTTCGTGGATCGCGGTTTCCTGGAGATCAAATCTC<br>CGATTCTGATTCCGCTGGAATACATTGAACGTATGGGCATCGATAATGATACCGAACT<br>GTCTAAACAGATCTTCCGTGTGGATAAAAACTTCTGTCTGCGTCCGATGCTGGCCCCG<br>AACATCTTCAACTATGGTCGTAAACTGGACCGTGCCCTGCCGGACCCGATCAAAATTT<br>TCGAGATCGGTCCTTGCTACCGTAAAGAGTCCGACGGTAAAGAGCACCTGGAAGAAT<br>TCACCATGCTGAACTTCTTCCAGATGGGTAGCGGTTGCACGCGTGAAAACCTGGAAT<br>CCATTATCACCGACTTCCTGAATCACCTGGGTATCGATTTCAAAATTGTTGGTGACAG<br>CTGTATGGTGTTTGGCGATACGCTGGATGTTATGCACGGCGATCTGGAGCTGTCTTCC<br>GCAGTAGTGGGCCCAATCCCGCTGGATCGTGAGTGGGGTATCGACAAACCTTGGATC<br>GGTGCGGGTTTTGGTCTGGAGCGTCTGCTGAAAGTAAAACACGACTTCAAGAACATC<br>AAACGTGCTGCACGTTCCGAGTCCTATTACAATGGTATTTCTACTAACCTGTAA
**Protein Sequence**	MDKKPLDVLISATGLWMSRTGTLHKIKHYEISRSKIYIEMACGDHLVVNNSRSCRPARAF<br>RYHKYRKTCKRCRVSDEDINNFLTRSTEGKTSVKVKVVSEPKVKKAMPKSVSRAPKPLE<br>NPVSAKASTDTSRSVPSPAKSTPNSPVPTSASAPALTKSQTDRLEVLLNPKDEISLNSGKP<br>FRELESELLSRRKKDLQQIYAEERENYLGKLEREITRFFVDRGFLEIKSPILIPLEYIERMGI<br>DNDTELSKQIFRVDKNFCLRPMLAPNIFNYGRKLDRALPDPIKIFEIGPCYRKESDGKEHL<br>EEFTMLNFFQMGSGCTRENLESIITDFLNHLGIDFKIVGDSCMVFGDTLDVMHGDLELSS<br>AVVGPIPLDREWGIDKPWIGAGFGLERLLKVKHDFKNIKRAARSESYYNGISTNL*

2	**chPylRS-IPYE-δVin-H** "IPYE" mutations (V31I, T56P, H62Y, and A100E) and fixed mutation (Y349F) were labeled in blue, and the mutations selected from the evolution were labeled in red.
**DNA sequence**	ATGGATAAGAAGCCGCTGGATGTTCTGATCTCTGCGACCGGTCTGTGGATGTCCCGTA<br>CCGGCACGCTGCACAAGATCAAGCACTATGAGATTTCTCGTTCTAAAATCTACATCGA<br>AATGGCGTGTGGTGACCATCTGGTTGTGAACAACTCTCGTTCTTGTCGTCCCGCACG<br>TGCATTCCGTTATCATAAATACCGTAAAACCTGCAAACGTTGTCGTGTTTCTGACGAA<br>GATATCAACAACTTCCTGACCCGTTCTACCGAAGGCAAAACCTCTGTTAAAGTTAAA<br>GTTGTTTCTGAGCCGAAAGTGAAAAAAGCGATGCCGAAATCTGTTTCTCGTGCGCCG<br>AAACCGCTGGAAAATCCGGTTTCTGCGAAAGCGTCTACCGACACCTCTCGTTCTGTT<br>CCGTCTCCGGCGAAATCTACCCCGAACTCTCCGGTTCCGACCTCTGCAAGCGCCCCA<br>GCTCTGACTAAATCCCAGACGGACCGTCTGGAGGTGCTGCTGAACCCAAAGGATGA<br>AATCTCTCTGAACAGCGGCAAGCCTTTCCGTGAGCTGGAAAGCGAGCTGCTGTCTCG<br>TCGTAAAAAGGATCTGCAACAGATCTACGCTGAGGAACGCGAGAACTATCTGGGTAA<br>GCTGGAGCGCGAAATTACTCGCTTCTTCGTGGATCGCGGTTTCCTGGAGATCAAATCT<br>CCGATTCTGATTCCGCTGGAATACATTGAACGTATGGGCATCGATAATGATACCGAAC<br>TGTCTAAACAGATCTTCCGTGTGGATAAAAACTTCTGTCTGCGTCCGATGCTGGCCCC<br>GAACATGTTGAACTATCTTCGTAAACTGGACCGTGCCCTGCCGGACCCGATCAAAAT<br>TTTCGAGATCGGTCCTTGCTACCGTAAAGAGTCCGACGGTAAAGAGCACCTGGAAGA<br>ATTCACCATGCTGGATTTCAAGCAGATGGGTAGCGGTTGCACGCGTGAAAACCTGGA<br>ATCCATTATCACCGACTTCCTGAATCACCTGGGTATCGATTTCAAAATTGTTGGTGACA<br>GCTGTATGGTGTTTGGCGATACGCTGGATGTTATGCACGGCGATCTGGAGCTGTCTTC<br>CGCAGTAGTGGGCCCAATCCCGCTGGATCGTGAGTGGGGTATCGACAAACCTTGGAT<br>CGGTGCGGGTTTTGGTCTGGAGCGTCTGCTGAAAGTAAAACACGACTTCAAGAACA<br>TCAAACGTGCTGCACGTTCCGAGTCCTATTACAATGGTATTTCTACTAACCTGTAA
**Protein Sequence**	MDKKPLDVLISATGLWMSRTGTLHKIKHYEISRSKIYIEMACGDHLVVNNSRSCRPARAF<br>RYHKYRKTCKRCRVSDEDINNFLTRSTEGKTSVKVKVVSEPKVKKAMPKSVSRAPKPLE<br>NPVSAKASTDTSRSVPSPAKSTPNSPVPTSASAPALTKSQTDRLEVLLNPKDEISLNSGKP<br>FRELESELLSRRKKDLQQIYAEERENYLGKLEREITRFFVDRGFLEIKSPILIPLEYIERMGI<br>DNDTELSKQIFRVDKNFCLRPMLAPNMLNYLRKLDRALPDPIKIFEIGPCYRKESDGKEH<br>LEEFTMLDFKQMGSGCTRENLESIITDFLNHLGIDFKIVGDSCMVFGDTLDVMHGDLEL<br>SSAVVGPIPLDREWGIDKPWIGAGFGLERLLKVKHDFKNIKRAARSESYYNGISTNL*

3	**Mm-PylRS-δMe-H(hit-2) in eukaryotic expression vector:**
**DNA sequence**	ATGGATAAAAAACCACTAAACACTCTGATATCTGCAACCGGGCTCTGGATGTCCAG<br>GACCGGAACAATTCATAAAATAAAACACCACGAAGTCTCTCGAAGCAAAATCTATA<br>TTGAAATGGCATGCGGAGACCACCTTGTTGTAAACAACTCCAGGAGCAGCAGGAC<br>TGCAAGAGCGCTCAGGCACCACAAATACAGGAAGACCTGCAAACGCTGCAGGGTT<br>TCGGATGAGGATCTCAATAAGTTCCTCACAAAGGCAAACGAAGACCAGACAAGCG<br>TAAAAGTCAAGGTCGTTTCTGCCCCTACCAGAACGAAAAAGGCAATGCCAAAATC<br>CGTTGCGAGAGCCCCGAAACCTCTTGAGAATACAGAAGCGGCACAGGCTCAACCT<br>TCTGGATCTAAATTTTCACCTGCGATACCGGTTTCCACCCAAGAGTCAGTTTCTGTC<br>CCGGCATCTGTTTCAACATCAATATCAAGCATTTCTACAGGAGCAACTGCATCCGCA<br>CTGGTAAAAGGGAATACGAACCCCATTACATCCATGTCTGCCCCTGTTCAGGCAAG<br>TGCCCCCGCACTTACGAAGAGCCAGACTGACAGGCTTGAAGTCCTGTTAAACCCA<br>AAAGATGAGATTTCCCTGAATTCCGGCAAGCCTTTCAGGGAGCTTGAGTCCGAATT<br>GCTCTCTCGCAGAAAAAAAGACCTGCAGCAGATCTACGCGGAAGAAAGGGAGAAT<br>TATCTGGGGAAACTCGAGCGTGAAATTACCAGGTTCTTTGTGGACAGGGGTTTTCT<br>GGAAATAAAATCCCCGATCCTGATCCCTCTTGAGTATATCGAAAGGATGGGCATTGA<br>TAATGATACCGAACTTTCAAAACAGATCTTCAGGGTTGACAAGAACTTCTGCCTGA<br>GACCCATGCTTGCTCCAAACATCTTCAACTACGGTCGCAAGCTTGACAGGGCCCTG<br>CCTGATCCAATAAAAATTTTTGAAATAGGCCCATGCTACAGAAAAGAGTCCGACGG<br>CAAAGAACACCTCGAAGAGTTTACCATGCTGAACTTCTTCCAGATGGGATCGGGAT<br>GCACACGGGAAAATCTTGAAAGCATAATTACGGACTTCCTGAACCACCTGGGAATT<br>GATTTCAAGATCGTAGGCGATTCCTGCATGGTCTTCGGGGATACCCTTGATGTAATG<br>CACGGAGACCTGGAACTTTCCTCTGCAGTAGTCGGACCCATACCGCTTGACCGGGA<br>ATGGGGTATTGATAAACCCTGGATAGGGGCAGGTTTCGGGCTCGAACGCCTTCTAA<br>AGGTTAAACACGACTTTAAAAATATCAAGAGAGCTGCAAGGTCCGAGTCTTACTAT<br>AACGGGATTTCTACCAACCTGTAA
**Protein Sequence**	MDKKPLNTLISATGLWMSRTGTIHKIKHHEVSRSKIYIEMACGDHLVVNNSRSSRTARA<br>LRHHKYRKTCKRCRVSDEDLNKFLTKANEDQTSVKVKVVSAPTRTKKAMPKSVARA<br>PKPLENTEAAQAQPSGSKFSPAIPVSTQESVSVPASVSTSISSISTGATASALVKGNTNPIT<br>SMSAPVQASAPALTKSQTDRLEVLLNPKDEISLNSGKPFRELESELLSRRKKDLQQIYA<br>EERENYLGKLEREITRFFVDRGFLEIKSPILIPLEYIERMGIDNDTELSKQIFRVDKNFCL<br>RPMLAPNIFNYGRKLDRALPDPIKIFEIGPCYRKESDGKEHLEEFTMLNFFQMGSGCTR<br>ENLESIITDFLNHLGIDFKIVGDSCMVFGDTLDVMHGDLELSSAVVGPIPLDREWGIDK<br>PWIGAGFGLERLLKVKHDFKNIKRAARSESYYNGISTNL*

4	**Mm-PylRS-δVin-H (hit-3) in eukaryotic expression vector:**
**DNA sequence**	ATGGATAAAAAACCACTAAACACTCTGATATCTGCAACCGGGCTCTGGATGTCCAG GACCGGAACAATTCATAAAATAAAACACCACGAAGTCTCTCGAAGCAAAATCTATA TTGAAATGGCATGCGGAGACCACCTTGTTGTAAACAACTCCAGGAGCAGCAGGAC TGCAAGAGCGCTCAGGCACCACAAATACAGGAAGACCTGCAAACGCTGCAGGGTT TCGGATGAGGATCTCAATAAGTTCCTCACAAAGGCAAACGAAGACCAGACAAGCG TAAAAGTCAAGGTCGTTTCTGCCCCTACCAGAACGAAAAAGGCAATGCCAAAATC CGTTGCGAGAGCCCCGAAACCTCTTGAGAATACAGAAGCGGCACAGGCTCAACCT TCTGGATCTAAATTTTCACCTGCGATACCGGTTTCCACCCAAGAGTCAGTTTCTGTC CCGGCATCTGTTTCAACATCAATATCAAGCATTTCTACAGGAGCAACTGCATCCGCA CTGGTAAAAGGGAATACGAACCCCATTACATCCATGTCTGCCCCTGTTCAGGCAAG TGCCCCCGCACTTACGAAGAGCCAGACTGACAGGCTTGAAGTCCTGTTAAACCCA AAAGATGAGATTTCCCTGAATTCCGGCAAGCCTTTCAGGGAGCTTGAGTCCGAATT GCTCTCTCGCAGAAAAAAAGACCTGCAGCAGATCTACGCGGAAGAAAGGGAGAAT TATCTGGGGAAACTCGAGCGTGAAATTACCAGGTTCTTTGTGGACAGGGGTTTTCT GGAAATAAAATCCCCGATCCTGATCCCTCTTGAGTATATCGAAAGGATGGGCATTGA TAATGATACCGAACTTTCAAAACAGATCTTCAGGGTTGACAAGAACTTCTGCCTGA GACCCATGCTTGCTCCAAACATGCTGAACTACCTGCGCAAGCTTGACAGGGCCCTG CCTGATCCAATAAAAATTTTTGAAATAGGCCCATGCTACAGAAAAGAGTCCGACGG CAAAGAACACCTCGAAGAGTTTACCATGCTGGACTTCAAGCAGATGGGATCGGGA TGCACACGGGAAAATCTTGAAAGCATAATTACGGACTTCCTGAACCACCTGGGAAT TGATTTCAAGATCGTAGGCGATTCCTGCATGGTCTTCGGGGATACCCTTGATGTAAT GCACGGAGACCTGGAACTTTCCTCTGCAGTAGTCGGACCCATACCGCTTGACCGGG AATGGGGTATTGATAAACCCTGGATAGGGGCAGGTTTCGGGCTCGAACGCCTTCTA AAGGTTAAACACGACTTTAAAAATATCAAGAGAGCTGCAAGGTCCGAGTCTTACTA TAACGGGATTTCTACCAACCTGTAA
**Protein Sequence**	MDKKPLNTLISATGLWMSRTGTIHKIKHHEVSRSKIYIEMACGDHLVVNNSRSSRTARA LRHHKYRKTCKRCRVSDEDLNKFLTKANEDQTSVKVKVVSAPTRTKKAMPKSVARA PKPLENTEAAQAQPSGSKFSPAIPVSTQESVSVPASVSTSISSISTGATASALVKGNTNPIT SMSAPVQASAPALTKSQTDRLEVLLNPKDEISLNSGKPFRELESELLSRRKKDLQQIYA EERENYLGKLEREITRFFVDRGFLEIKSPILIPLEYIERMGIDNDTELSKQIFRVDKNFCL RPMLAPNMLNYLRKLDRALPDPIKIFEIGPCYRKESDGKEHLEEFTMLDFKQMGSGCT RENLESIITDFLNHLGIDFKIVGDSCMVFGDTLDVMHGDLELSSAVVGPIPLDREWGID KPWIGAGFGLERLLKVKHDFKNIKRAARSESYYNGISTNL*

**5**	**GFP-HisTag in prokaryotic expression vector**
**DNA sequence**	ATGGGTAAAGGAGAAGAACTTTTCACTGGAGTTGTCCCAATTCTTGTTGAATTAGAT<br>GGTGATGTTAATGGGCACAAATTTTCTGTCAGTGGAGAGGGTGAAGGTGATGCAAC<br>ATACGGAAAACTTACCCTTAAATTTATTTGCACTACTGGAAAACTACCTGTTCCATGG<br>CCAACACTTGTCACTACTTTCTCTTATGGTGTTCAATGCTTTTCCCGTTATCCGGATCA<br>CATGAAACGGCATGACTTTTTCAAGAGTGCCATGCCCGAAGGTTATGTACAGGAACG<br>CACTATATCTTTCAAAGATGACGGGAACTACAAGACGCGTGCTGAAGTCAAGTTTGA<br>AGGTGATACCCTTGTTAATCGTATCGAGTTAAAAGGTATTGATTTTAAAGAAGATGGA<br>AACATTCTCGGACACAAACTCGAGTACAACTATAACTCACACAATGTATACATCACG<br>GCAGACAAACAAAAGAATGGAATCAAAGCTAACTTCAAAATTCGCCACAACATTGA<br>AGATGGATCCGTTCAACTAGCAGACCATTATCAACAAAATACTCCAATTGGCGATGG<br>CCCTGTCCTTTTACCAGACAACCATTACCTGTCGACACAATCTGCCCTTTCGAAAGAT<br>CCCAACGAAAAGCGTGACCACATGGTCCTTCTTGAGTTTGTAACTGCTGCTGGGATT<br>ACACATGGCATGGATGAACTCTACAAAGGGCCCCATCATCACCATCACCATTGA
**Protein Sequence**	MGKGEELFTGVVPILVELDGDVNGHKFSVSGEGEGDATYGKLTLKFICTTGKLPVPWPT<br>LVTTFSYGVQCFSRYPDHMKRHDFFKSAMPEGYVQERTISFKDDGNYKTRAEVKFEGD<br>TLVNRIELKGIDFKEDGNILGHKLEYNYNSHNVYITADKQKNGIKANFKIRHNIEDGSVQ<br>LADHYQQNTPIGDGPVLLPDNHYLSTQSALSKDPNEKRDHMVLLEFVTAAGITHGMDE<br>LYKGPHHHHHH*

**6**	**GFP-FLAG in eukaryotic expression vector:**
**DNA sequence**	ATGGTGAGCAAGGGCGAGGAGCTGTTCACCGGGGTGGTGCCCATCCTGGTCGAGCT<br>GGACGGCGACGTAAACGGCCACAAGTTCAGCGTGTCCGGCGAGGGCGAGGGCGAT<br>GCCACCTATGGCAAGCTGACCCTGAAGTTCATCTGCACCACCGGCAAGCTGCCCGTG<br>CCCTGGCCCACCCTCGTGACCACCCTGACCTACGGCGTGCAGTGCTTCAGCCGCTAC<br>CCCGACCACATGAAGCAGCACGACTTCTTCAAGTCCGCCATGCCCGAAGGCTACGT<br>CCAGGAGCGCACCATCTTCTTCAAGGACGACGGCAACTACAAGACCCGCGCCGAGG<br>TGAAGTTCGAGGGCGACACCCTGGTGAACCGCATCGAGCTGAAGGGCATCGACTTC<br>AAGGAGGACGGCAACATCCTGGGGCACAAGCTGGAGTACAACTACAACAGCCACA<br>ACGTCTATATCATGGCCGACAAGCAGAAGAACGGCATCAAGGTGAACTTCAAGATCC<br>GCCACAACATCGAGGACGGCAGCGTGCAGCTCGCCGACCACTACCAGCAGAACAC<br>CCCCATCGGCGACGGCCCCGTGCTGCTGCCCGACAACCACTACCTGAGCACCCAGT<br>CCGCCCTGAGCAAAGACCCCAACGAGAAGCGCGATCACATGGTCCTGCTGGAGTTC<br>GTGACCGCCGCCGGGATCACTCTCGGCATGGACGAGCTGTACAaGGATTACAAGgAT<br>GACGACGATAAGTAA
**Protein Sequence**	MVSKGEELFTGVVPILVELDGDVNGHKFSVSGEGEGDATYGKLTLKFICTTGKLPVPWP<br>TLVTTLTYGVQCFSRYPDHMKQHDFFKSAMPEGYVQERTIFFKDDGNYKTRAEVKFEG<br>DTLVNRIELKGIDFKEDGNILGHKLEYNYNSHNVYIMADKQKNGIKVNFKIRHNIEDGS<br>VQLADHYQQNTPIGDGPVLLPDNHYLSTQSALSKDPNEKRDHMVLLEFVTAAGITLGM<br>DELYKDYKDDDDK*

7	**Myoglobin-6×His in prokaryotic expression vector:**
**DNA sequence**	ATGGTTCTGTCTGAAGGTGAATGGCAGCTGGTTCTGCATGTTTGGGCTAAAGTTGAA<br>GCTGACGTCGCTGGTCATGGTCAGGACATCTTGATTCGACTGTTCAAATCTCATCCG<br>GAAACTCTGGAAAAATTCGATCGTTTCAAACATCTGAAAACTGAAGCTGAAATGAA<br>AGCTTCTGAAGATCTGAAAAAAGTGGGTGTTACCGCGTTAACTGCCCTAGGTGCTAT<br>CCTTAAGAAAAAAGGGCATCATGAAGCTGAGCTCAAACCGCTTGCACAATCGCATG<br>CTACTAAACATAAGATCCCGATCAAATACCTGGAATTCATCTCTGAAGCGATCATCCA<br>TGTTCTGCATTCTAGACATCCAGGTGACTTCGGTGCTGACGCTCAGGGTGCTATGAA<br>CAAAGCTCTGGAGCTGTTCCGTAAAGATATCGCTGCTAAGTACAAAGAACTGGGTTA<br>CCAGGGTGGCTCGGGACATCATCACCATCACCATTGA
**Protein Sequence**	MVLSEGEWQLVLHVWAKVEADVAGHGQDILIRLFKSHPETLEKFDRFKHLKTEAEMK<br>ASEDLKKVGVTALTALGAILKKKGHHEAELKPLAQSHATKHKIPIKYLEFISEAIIHVLHS<br>RHPGDFGADAQGAMNKALELFRKDIAAKYKELGYQGGSGHHHHHH*

8	**APEX2-6×His in prokaryotic expression vector:**
**DNA sequence**	ATGGGTAAATCTTACCCGACCGTTTCTGCGGACTACCAGGACGCGGTTGAAAAAGC<br>GAAAAAAAAACTGCGTGGTTTCATCGCGGAAAAACGTTGCGCGCCGCTGATGCTGC<br>GTCTGGCGTTCCACTCTGCGGGTACCTTCGACAAAGGTACCAAAACCGGTGGTCCG<br>TTCGGTACCATCAAACACCCGGCGGAACTGGCGCACTCTGCGAACAACGGTCTGGA<br>CATCGCGGTTCGTCTGCTGGAACCGCTGAAAGCGGAATTCCCGATCCTGTCTTACGC<br>GGACTTCTACCAGCTGGCGGGTGTTGTTGCGGTTGAAGTTACCGGTGGTCCGAAAG<br>TTCCGTTCCACCCGGGTCGTGAAGACAAACCGGAACCGCCGCCGGAAGGTCGTCTG<br>CCGGACCCGACCAAAGGTTCTGACCACCTGCGTGACGTTTTCGGTAAAGCGATGGG<br>TCTGACCGACCAGGACATCGTTGCGCTGTCTGGTGGTCATACCATCGGTGCGGCGCA<br>CAAAGAACGTTCTGGTTTCGAAGGTCCGTGGACCTCTAACCCGCTGATCTTCGACAA<br>CTCTTACTTCACCGAACTGCTGTCTGGTGAAAAAGAAGGTCTGCTGCAGCTGCCGTC<br>TGACAAAGCGCTGCTGTCTGACCCGGTTTTCCGTCCGCTGGTTGACAAATACGCGGC<br>GGACGAAGACGCGTTCTTCGCGGACTACGCGGAAGCGCACCAGAAACTGTCTGAAC<br>TGGGTTTCGCGGACGCGCATCATCATCATCATCATTGA
**Protein Sequence**	MGKSYPTVSADYQDAVEKAKKKLRGFIAEKRCAPLMLRLAFHSAGTFDKGTKTGGPF<br>GTIKHPAELAHSANNGLDIAVRLLEPLKAEFPILSYADFYQLAGVVAVEVTGGPKVPFHP<br>GREDKPEPPPEGRLPDPTKGSDHLRDVFGKAMGLTDQDIVALSGGHTIGAAHKERSGFE<br>GPWTSNPLIFDNSYFTELLSGEKEGLLQLPSDKALLSDPVFRPLVDKYAADEDAFFADYA<br>EAHQKLSELGFADAHHHHHH*

9	dnHEM1.2-6×His in prokaryotic expression vector:
DNA sequence	ATGGTGAGCCTGGATCAGGCGATTGATATTCTGGTGGTGGCGGCGAAACTGGGCACC<br>ACCGTGGAAGAAGCGGTGAAACGCGCGCTGTGGCTGAAAACCAAATTAGGCGTGTC<br>GTTGGACCAGGCGCTGCGTATTCTGAGCGATGCCGCCAATACCGGCACGACGGTTGA<br>AGAGGCCGTTAAACGTGCACTGAAACTGAAGACGAAGCTCGGTGTTTCGTTAGAGG<br>CGGCGCTGGCGATTTTAAGCGCAGCCGCGCAGCTGGGTACTACTGTGGAGGAGGCG<br>GTTAAGCGCGCGTTGAAATTGAAAACGAAGTTGGGCGTGGATCTGGAAACCGCGGC<br>CTTAGCGTTGTTGACCGCAGCCAAGTTAGGTACGACCGTTGAGGAAGCAGTTAAGC<br>GCGCCCTGAAGTTAAAGACCAAGTTGGGTGTGAGCTTGATTGAGGCACTGCATATTC<br>TGCTGACTGCCGCGGTGTTAGGCACTACCGTCGAAGAGGCGGTGTATCGCGCCTTGA<br>AGTTGAAAACTAAATTGGGGGTTAGTCTGCTGCAGGCGGCTGCCATCTTGCTTTTAG<br>CAGCCCGCCTGGGGACTACGGTGGAGGAGGCCGTAAAGCGTGCCTTAAAATTAAAA<br>ACCAAATTGGGTGGGGGCAGCGGTGGCAGCCATCATTGGGGCTCGGGCTCGCATCA<br>TCACCACCATCATTGA
Protein Sequence	MVSLDQAIDILVVAAKLGTTVEEAVKRALWLKTKLGVSLDQALRILSDAANTGTTVEE<br>AVKRALKLKTKLGVSLEAALAILSAAAQLGTTVEEAVKRALKLKTKLGVDLETAALAL<br>LTAAKLGTTVEEAVKRALKLKTKLGVSLIEALHILLTAAVLGTTVEEAVYRALKLKTKL<br>GVSLLQAAAILLLAARLGTTVEEAVKRALKLKTKLGGGSGGSHHWGSGSHHHHHH*

10	OE1.3-StrepTagII in prokaryotic expression vector:
DNA sequence	ATGATTCGTGCGGTATTCTTTGATAGCCCGGGTACTCTGAATAGCGTTGAAGGTCATG<br>CTAAAATGCATCTGAAAATTATGGAGGAAGTGCTGGGTGACTATCCGCTGAACCCGA<br>AAACCCTTCTTGACGAATACAATAAACTGACCCGCGAAGCGTTCTCTAACTATGCGG<br>GCAAACCGTATCGCGGTCTGCGTGATATCCTGGAAGAAGTAATGCGTAAACTGGCGG<br>AAAAGTACGGTTTCAAATACCCTGAAAACTTCTGGGAAATCTCCCTGCGCATGTCTC<br>AACGCTACGGCGAGCTGTACCCGGAAGTGGTGGAAGTACTGAAATCTCTGAAAGGT<br>AAATATCACGTTGGCATGATCACCGATTCGGGTACCGAGCAGGCCATGGCATTCCTG<br>GACGCACTGGGCATCAAAGACCTGTTCGATTCCATCACCACGTCTGAAGAAGCTGG<br>TTTCTTTAAACCGCACCCACGCATCTTCGAACTGGCTCTGAAGAAAGCCGGCGTTAA<br>AGGCGAGGAAGCAGTGTACGTTGGTGACAACCCGGTCAAAGACTGTGGTGGTTCTA<br>AGAACCTGGGTATGACTAGCATCCTGCTGGATCGTAAAGGTGAGAAACGTGAATTCT<br>GGGATAAGTGCGACTTTATCGTCTCCGACCTGCGCGAAGTTATTAAGATTGTTGACG<br>AACTGAACGGTCAGGGCTCTTGGAGTCACCCACAGTTTGAGAAATGA
Protein Sequence	MIRAVFFDSPGTLNSVEGHAKMHLKIMEEVLGDYPLNPKTLLDEYNKLTREAFSNYAG<br>KPYRGLRDILEEVMRKLAEKYGFKYPENFWEISLRMSQRYGELYPEVVEVLKSLKGKY<br>HVGMITDSGTEQAMAFLDALGIKDLFDSITTSEEAGFFKPHPRIFELALKKAGVKGEEA<br>VYVGDNPVKDCGGSKNLGMTSILLDRKGEKREFWDKCDFIVSDLREVIKIVDELNGQG<br>SWSHPQFEK*

11	**P450-BM3-HStar-8×His in prokaryotic expression vector:**
**DNA sequence**	ATGACCATCAAAGAAATGCCGCAGCCGAAAACCTTCGGTGAACTGAAAAACCTGCC<br>GCTGCTGAACACCGACAAACCGGTTCAGGCGCTGATGAAAATCGCGGACGAACTGG<br>GTGAAATCTTCAAATTCGAAGCGCCGGGTCGTGTTACCCGTTACCTGTCTTCTCAGC<br>GTCTGATCAAAGAAGCGTGCGACGAATCTCGTTTCGACAAAAACCTGTCTCAGGCG<br>CTGAAATTCATGCGTGACTTCGCGGGTGACGGTCTGTTCACCTCTTGGACCCACGAA<br>AAAAACTGGAAAAAAGCGCACAACATCCTGCTGCCGTCTTTCTCTCAGCAGGCGAT<br>GAAAGGTTACCACGCGATGATGGTTGACATCGCGGTTCAGCTGGTTCAGAAATGGG<br>AACGTCTGAACGCGGACGAACACATCGAAGTTCCGGAAGACATGACCCGTCTGACC<br>CTGGACACCATCGGTCTGTGCGGTTTCAACTACCGTTTCAACTCTTTCTACCGTGACC<br>AGCCGCACCCGTTCATCACCTCTATGGTTCGTGCGGTTGACGAAGCGATGAACAAAC<br>TGCAGCGTGCGAACCCGGACGACCCGGCGTACGACGAAAACAAACGTCAGTTCCA<br>GGAAGACATCAAAGTTATGAACGACCTGGTTGACAAAATCATCGCGGACCGTAAAG<br>CGTCTGGTGAACAGTCTGACGACCTGCTGACCCACATGCTGAACGGTAAAGACCCG<br>GAAACCGGTGAACCGCTGGACGACGAAAACATCCGTTACCAGATCATCACCTTCCT<br>GATCGCGGGTCACGAAGCGACCTCTGGTCTGCTGTCTTTCGCGCTGTACTTCCTGGT<br>TAAAAACCCGCACGTTCTGCAGAAAGCGGCGGAAGAAGCGGCGCGTGTTCTGGTTG<br>ACCCGGTTCCGTCTTACAAACAGGTTAAACAGCTGAAATACGTTGGTATGGTTCTGA<br>ACGAAGCGCTGCGTCTGTGGCCGACCGCGCCGGCGTTCTCTCTGTACGCGAAAGAA<br>GACACCGTTCTGGGTGGTGAATACCCGCTGGAAAAAGGTGACGAACTGATGGTTCT<br>GATCCCGCAGCTGCACCGTGACAAAACCATCTGGGGTGACGACGTTGAAGAATTCC<br>GTCCGGAACGTTTCGAAAACCCGTCTGCGATCCCGCAGCACGCGTTCAAACCGTTC<br>GGTAACGGTCAGCGTGCGCATATCGGTCAGCAGTTCGCGCTGCACGAAGCGACCCT<br>GGTTCTGGGTATGATGCTGAAACACTTCGACTTCGAAGACCACACCAACTACGAACT<br>GGACATCAAAGAAACCTGGACCCTGAAACCGGAAGGTTTCGTTGTTAAAGCGAAAT<br>CTAAAAAAATCCCGCTGGGTGGTATCCCGTCTCCGTCTACCGAACAGTCTGCGAAAA<br>AAGTTCGTAAAAAAGCGGAAAACGCGCACAACACCCCGCTGCTGGTTCTGTACGGT<br>TCTAACATGGGTACCGCGGAAGGTACCGCGCGTGACCTGGCGGACATCGCGATGTCT<br>AAAGGTTTCGCGCCGCAGGTTGCGACCCTGGACTCTCACGCGGGTAACCTGCCGCG<br>TGAAGGTGCGGTTCTGATCGTTACCGCGTCTTACAACGGTCACCCGCCGGACAACGC<br>GAAACAGTTCGTTGACTGGCTGGACCAGGCGTCTGCGGACGAAGTTAAAGGTGTTC<br>GTTACTCTGTTTTCGGTTGCGGTGACAAAAACTGGGCGACCACCTACCAGAAAGTTC<br>CGGCGTTCATCGACGAAACCCTGGCGGCGAAAGGTGCGGAAAACATCGCGGACCGT<br>GGTGAAGCGGACGCGTCTGACGACTTCGAAGGTACCTACGAAGAATGGCGTGAACA<br>CATGTGGTCTGACGTTGCGGCGTACTTCAACCTGGACATCGAAAACTCTGAAGACA<br>ACAAATCTACCCTGTCTCTGCAGTTCGTTGACTCTGCGGCGGACATGCCGCTGGCGA<br>AAATGCACGGTGCGTTCTCTACCAACGTTGTTGCGTCTAAAGAACTGCAGCAGCCG<br>GGTTCTGCGCGTTCTACCCGTCACCTGGAAATCGAACTGCCGAAAGAAGCGTCTTAC<br>CAGGAAGGTGACCACCTGGGTGTTATCCCGCGTAACTACGAAGGTATCGTTAACCGT<br>GTTACCGCGCGTTTCGGTCTGGACGCGTCTCAGCAGATCCGTCTGGAAGCGGAAGA<br>AGAAAAACTGGCGCACCTGCCGCTGGCGAAAACCGTTTCTGTTGAAGAACTGCTGC<br>AGTACGTTGAACTGCAGGACCCGGTTACCCGTACCCAGCTGCGTGCGATGGCGGCG<br>AAAACCGTTTGCCCGCCGCACAAAGTTGAACTGGAAGCGCTGCTGGAAAAACAGG<br>CGTACAAAGAACAGGTTCTGGCGAAACGTCTGACCATGCTGGAACTGCTGGAAAAA<br>TACCCGGCGTGCGAAATGAAATTCTCTGAATTCATCGCGCTGCTGCCGTCTATCCGTC<br>CGCGTTACTACTCTATCTCTTCTTCTCCGCGTGTTGACGAAAAACAGGCGTCTATCAC<br>CGTTTCTGTTGTTTCTGGTGAAGCGTGGTCTGGTTACGGTGAATACAAAGGTATCGC

**DNA sequence**	GTCTAACTACCTGGCGGAACTGCAGGAAGGTGACACCATCACCTGCTTCATCTCTAC<br>CCCGCAGTCTGAATTCACCCTGCCGAAAGACCCGGAAACCCCGCTGATCATGGTTG<br>GTCCGGGTACCGGTGTTGCGCCGTTCCGTGGTTTCGTTCAGGCGCGTAAACAGCTGA<br>AAGAACAGGGTCAGTCTCTGGGTGAAGCGCACCTGTACTTCGGTTGCCGTTCTCCG<br>CACGAAGACTACCTGTACCAGGAAGAACTGGAAAACGCGCAGTCTGAAGGTATCAT<br>CACCCTGCACACCGCGTTCTCTCGTATGCCGAACCAGCCGAAAACCTACGTTCAGCA<br>CGTTATGGAACAGGACGGTAAAAAACTGATCGAACTGCTGGACCAGGGTGCGCACT<br>TCTACATCTGCGGTGACGGTTCTCAGATGGCGCCGGCGGTTGAAGCGACCCTGATGA<br>AATCTTACGCGGACGTTCACCAGGTTTCTGAAGCGGACGCGCGTCTGTGGCTGCAG<br>CAGCTGGAAGAAAAAGGTCGTTACGCGAAAGACGTTTGGGCGGGTCTGGAACATCA<br>TCACCACCATCACCATCATTGA
**Protein Sequence**	MTIKEMPQPKTFGELKNLPLLNTDKPVQALMKIADELGEIFKFEAPGRVTRYLSSQRLIK<br>EACDESRFDKNLSQALKFMRDFAGDGLFTSWTHEKNWKKAHNILLPSFSQQAMKGYH<br>AMMVDIAVQLVQKWERLNADEHIEVPEDMTRLTLDTIGLCGFNYRFNSFYRDQPHPFIT<br>SMVRAVDEAMNKLQRANPDDPAYDENKRQFQEDIKVMNDLVDKIIADRKASGEQSDD<br>LLTHMLNGKDPETGEPLDDENIRYQIITFLIAGHEATSGLLSFALYFLVKNPHVLQKAAEE<br>AARVLVDPVPSYKQVKQLKYVGMVLNEALRLWPTAPAFSLYAKEDTVLGGEYPLEKG<br>DELMVLIPQLHRDKTIWGDDVEEFRPERFENPSAIPQHAFKPFGNGQRAHIGQQFALHE<br>ATLVLGMMLKHFDFEDHTNYELDIKETWTLKPEGFVVKAKSKKIPLGGIPSPSTEQSAK<br>KVRKKAENAHNTPLLVLYGSNMGTAEGTARDLADIAMSKGFAPQVATLDSHAGNLPRE<br>GAVLIVTASYNGHPPDNAKQFVDWLDQASADEVKGVRYSVFGCGDKNWATTYQKVPA<br>FIDETLAAKGAENIADRGEADASDDFEGTYEEWREHMWSDVAAYFNLDIENSEDNKST<br>LSLQFVDSAADMPLAKMHGAFSTNVVASKELQQPGSARSTRHLEIELPKEASYQEGDH<br>LGVIPRNYEGIVNRVTARFGLDASQQIRLEAEEEKLAHLPLAKTVSVEELLQYVELQDP<br>VTRTQLRAMAAKTVCPPHKVELEALLEKQAYKEQVLAKRLTMLELLEKYPACEMKFS<br>EFIALLPSIRPRYYSISSSPRVDEKQASITVSVVSGEAWSGYGEYKGIASNYLAELQEGDT<br>ITCFISTPQSEFTLPKDPETPLIMVGPGTGVAPFRGFVQARKQLKEQGQSLGEAHLYFGC<br>RSPHEDYLYQEELENAQSEGIITLHTAFSRMPNQPKTYVQHVMEQDGKKLIELLDQGAH<br>FYICGDGSQMAPAVEATLMKSYADVHQVSEADARLWLQQLEEKGRYAKDVWAGLEHH<br>HHHHHH*

Supplementary References

1. Yar, M., McGarrigle, E. M. & Aggarwal, V. K. An Annulation Reaction for the Synthesis of Morpholines, Thiomorpholines, and Piperazines from β-Heteroatom Amino Compounds and Vinyl Sulfonium Salts. *Angew. Chem. Int. Ed.* **47**, 3784-3786 (2008).

2. Lavis, L. D., Chao, T.-Y. & Raines, R. T. Synthesis and utility of fluorogenic acetoxymethyl ethers. *Chem. Sci.* **2**, 521-530 (2011).
